# Cell type-specific histone acetylation profiling of Alzheimer’s disease subjects and integration with genetics

**DOI:** 10.3389/fnmol.2022.948456

**Published:** 2023-01-06

**Authors:** Easwaran Ramamurthy, Gwyneth Welch, Jemmie Cheng, Yixin Yuan, Laura Gunsalus, David A. Bennett, Li-Huei Tsai, Andreas R. Pfenning

**Affiliations:** ^1^Computational Biology Department, School of Computer Science, Carnegie Mellon University, Pittsburgh, PA, United States; ^2^Department of Brain and Cognitive Sciences, Picower Institute for Learning and Memory, Massachusetts Institute of Technology, Cambridge, MA, United States; ^3^Rush Alzheimer’s Disease Center, Rush University Medical Center, Chicago, IL, United States; ^4^Broad Institute of MIT and Harvard, Cambridge, MA, United States

**Keywords:** Epigenomics, Alzheimer’s disease, brain cell types, genetics, genomics

## Abstract

We profile genome-wide histone 3 lysine 27 acetylation (H3K27ac) of 3 major brain cell types from hippocampus and dorsolateral prefrontal cortex (dlPFC) of subjects with and without Alzheimer’s Disease (AD). We confirm that single nucleotide polymorphisms (SNPs) associated with late onset AD (LOAD) show a strong tendency to reside in microglia-specific gene regulatory elements. Despite this significant colocalization, we find that microglia harbor more acetylation changes associated with age than with amyloid-β (Aβ) load. In contrast, we detect that an oligodendrocyte-enriched glial (OEG) population contains the majority of differentially acetylated peaks associated with Aβ load. These differential peaks reside near both early onset risk genes (*APP, PSEN1, PSEN2*) and late onset AD risk loci (including *BIN1, PICALM, CLU, ADAM10, ADAMTS4, SORL1, FERMT2*), Aβ processing genes (*BACE1*), as well as genes involved in myelinating and oligodendrocyte development processes. Interestingly, a number of LOAD risk loci associated with differentially acetylated risk genes contain H3K27ac peaks that are specifically enriched in OEG. These findings implicate oligodendrocyte gene regulation as a potential mechanism by which early onset and late onset risk genes mediate their effects, and highlight the deregulation of myelinating processes in AD. More broadly, our dataset serves as a resource for the study of functional effects of genetic variants and cell type specific gene regulation in AD.

## Introduction

Alzheimer’s Disease (AD) is the most common age-related neurodegenerative disorder (Gaugler et al., 2016). The hallmarks of AD pathology are numerous and include neuronal loss, synaptic dysfunction, gliosis, and the accumulation of amyloid-β (Aβ) protein and neurofibrillary tangles (NFT) formed from phosphorylated tau protein (MAPT) (Hardy and Selkoe, 2002). Aβ plaques are formed by differential proteolytic cleavage of the amyloid β precursor protein (*APP*) (Goldgaber et al., 1987; Kang et al., 1987; Robakis et al., 1987; Tanzi et al., 1987) by the α-secretase, β-secretase, and γ-secretase enzymes (De Strooper, 2010). Studies of individuals affected by early onset (<60 years) familial AD (EOAD) have identified causal autosomal dominant mutations primarily in the Aβ processing proteins presenilin-1 (*PSEN1*) and presenilin-2 (*PSEN2*), which are part of the γ-secretase complex (Schellenberg et al., 1993; Levy-Lahad et al., 1995; Rogaev et al., 1995). Causal mutations have also been found in *APP* itself (St George-Hyslop et al., 1987; Goate et al., 1991; Gatz et al., 2006; Goate, 2006). However, EOAD accounts for a minority of AD cases (∼1–6%). Late onset sporadic AD (LOAD) is more frequent and accounts for the majority of AD cases (Bekris et al., 2010).

However, in contrast to EOAD, the genetic risk for LOAD is less clear. Currently, the strongest known genetic risk factor for LOAD is the ε4 allele of Apolipoprotein E (*APOE*) (Corder et al., 1993; Poirier et al., 1993; Saunders et al., 1993; Roses, 1996; Saunders, 2000). More recently, genome wide association studies (GWAS) (Harold et al., 2009; Lambert et al., 2009, 2013; Seshadri et al., 2010; Hollingworth et al., 2011; Naj et al., 2011; Jansen et al., 2019; Kunkle et al., 2019) have identified an additional 28 unique loci harboring genetic variants that increase risk for developing LOAD (Bertram and Tanzi, 2019; Jansen et al., 2019; Kunkle et al., 2019). Notably, only 2% of GWAS-derived AD-associated sentinel genetic variants [primarily single nucleotide polymorphisms (SNPs)] localize to known exons (Kunkle et al., 2019). Since these SNPs do not alter protein-coding sequences, it is difficult to trace their functional importance in disease onset and progression.

To this end, epigenomic studies are revealing that these SNPs likely alter the function of gene regulatory elements in LOAD. Indeed, 26% of these SNPs localize in regions containing promoter histone marks, 69% lie in enhancer states, and 46% lie in DNase I accessible sites (Ward and Kellis, 2012; Kunkle et al., 2019). Furthermore, non-coding SNPs were found at sites of altered histone 3 lysine 27 acetylation (H3K27ac) in the human postmortem AD brain (Marzi et al., 2018; Nativio et al., 2018). H3K27ac is a histone residue associated with transcriptionally active promoters and enhancers (Creyghton et al., 2010). Gene regulatory elements, especially enhancers, are highly context-specific with differing activities across tissues, cell types and environments (Consortium et al., 2015). Therefore, it is likely that different cell types in the brain orchestrate different regulatory programs during AD progression. Indeed, many LOAD risk loci are primarily implicated in immune function, suggesting immune cell types may mediate their effects (Gjoneska et al., 2015; Huang et al., 2017; Lake et al., 2017). Recently, these SNPs were confirmed to reside primarily within microglia-specific enhancers (Nott et al., 2019). Additionally, alterations in H3K27ac in AD vs. non-AD brain tissue is not restricted to AD risk loci, indicating other regulatory mechanisms are altered beyond those associated with AD GWAS SNPs.

Notably, many previous studies of histone acetylation in post-mortem brain samples were performed utilizing whole brain tissue, and not all were performed with tissue from AD patients (Marzi et al., 2018; Klein et al., 2019). This may obscure changes that occur within specific cell populations. We address this gap in knowledge by profiling H3K27ac in individual cell types from AD and non-AD postmortem brain tissue. We utilize fluorescence-activated nuclei sorting (FANS) (Marion-Poll et al., 2014) to purify neuronal, microglial and other glial populations in the dorsolateral prefrontal cortex (dlPFC) and hippocampus. We then perform H3K27ac chromatin immunoprecipitation followed by sequencing (ChIP-seq) to mark putative regulatory elements in these populations. In addition to establishing the first genome-wide H3K27ac profiles in neuronal, microglial, and oligodendrocyte-enriched glial (OEG) populations from AD postmortem brain samples, our cell type-specific approach confirms that GWAS-derived LOAD risk loci are primarily enriched in microglia-specific H3K27ac peaks. Despite the well-established roles of microglial and neuronal processes in the progression of AD, we detect the vast majority of disease-associated H3K27ac dysregulation in the oligodendrocyte-enriched glia population. Interestingly, many of these altered peaks occur near AD risk genes, Aβ processing genes, and myelin-associated genes. Due to the limited size of our cohort however, further validation of these oligodendrocyte H3K27ac alterations are required. Despite this caveat, these findings suggest distinct gene-regulatory mechanisms of AD onset and progression in different brain cell types and highlight specific cell types, loci, and pathways for future study.

## Results

### Fluorescence-activated nuclei sorting and H3K27ac chromatin immunoprecipitation followed by sequencing of dorsolateral prefrontal cortex and hippocampus

We obtained 10 dlPFC and 16 hippocampus samples from 19 participants in the Religious Orders Study and Memory and Aging Project (ROSMAP) (Bennett et al., 2012a,b, 2018) (mean age = 87.84 years, s.d. = 7.75, range = 74.77–101.94). We classified 5 of 10 dlPFC samples and 10 of 16 hippocampus samples as “high Aβ.” These samples were from individuals that displayed high Aβ load across the brain, (mean percentage area occupied by Aβ across 8 brain regions = 7.30, s.d = 4.14, range = 2.31–15.40), high overall neurofibrillary tangle density (mean density of NFT across 8 brain regions = 22.81, s.d. = 13.73, range = 1.80–61.01) and low global cognition scores (mean cogn_global_lv = −2.1, s.d. = 1.46, range = −3.87–0.51). Samples classified as “no Aβ” had 0 Aβ load, mild neurofibrillary tangle load (mean density of NFT across 8 brain regions = 0.84, s.d. = 0.6, range = 0.11–2.05), and were not cognitively impaired (mean cogn_global_lv = −0.3, s.d. = 0.6, range = −1.6–0.27) (Supplementary Table 1 and Supplementary Figure 1). The self-reported sex of 6 of the 10 dlPFC samples was male, and the remaining 4 were female. Of the 16 hippocampus samples, the self-reported sex of 6 was male, and the remaining 10 were female. Our sample sets were controlled for age at death (high Aβ mean age = 88.77 yrs., s.d = 7.0, no Aβ mean age = 86.25 yrs., s.d = 8.04; unpaired *t*-test for difference in means *p*-value = 0.51), years of education (high Aβ mean educ = 20 yrs., s.d = 2.12, no Aβ mean educ = 18.85 yrs., s.d = 2.94, unpaired *t*-test *p* = 0.37), and postmortem interval (high Aβ mean pmi = 9.04 h, s.d. = 6.02, no Aβ mean pmi = 9.87 h, s.d. = 5.49; unpaired *t*-test *p* = 0.78). We provide additional information regarding our sample set in Supplementary Figures 2, 3.

**FIGURE 1:**
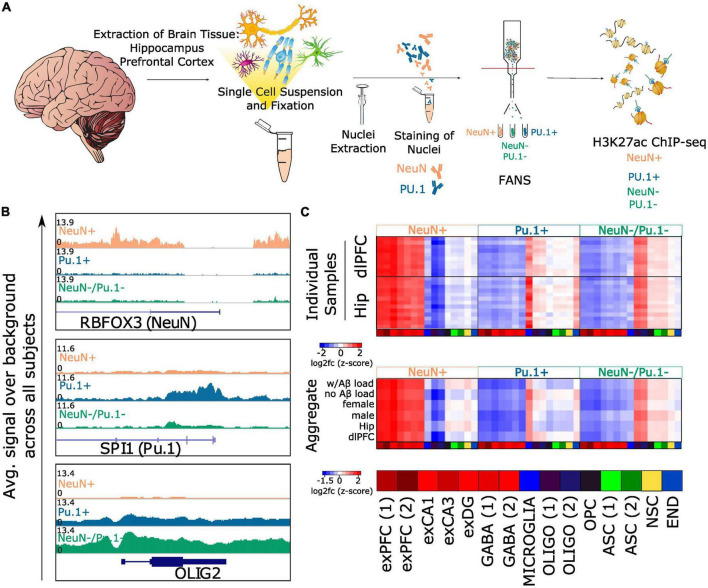
FANS sorting captures neurons, microglia and oligodendrocyte enriched populations from postmortem brain tissue. **(A)** Workflow for sorting nuclei and performing H3K27ac ChIP-seq from postmortem human brain tissue: nuclei were isolated from fresh frozen hippocampus or prefrontal cortex and FANS was performed to collect NeuN+, Pu.1+, and NeuN-/Pu.1- populations. H3K27ac ChIP-seq was performed on each population **(B)**. Genome browser visualization of H3K27ac signal over background (Input) averaged across all profiled samples for the three populations. Loci containing the genes RBFOX3 (NeuN), SPI1 (Pu.1) and OLIG2 (an oligodendrocyte marker) are visualized **(C)**. Top heatmap displaying average H3K27ac enrichment at the promoters of marker genes (<5kb from TSS) from 15 cell type clusters profiled in Habib et al. (2017). Rows represent individual tissue samples. Columns represent the 15 different cell type clusters and are repeated three times to display NeuN+ specificity, Pu.1+ specificity and NeuN-/Pu.1- specificity. Bottom collapsed version of the top heatmap created by averaging the log2fc values for groups of samples defined by Aβ load, sex and brain region. Habib et al. (2017) cell type cluster abbreviations are defined here: exPFC, glutamatergic neurons from the PFC; GABA, GABAergic interneurons; exCA1/3, pyramidal neurons from the hippocampus CA region; exDG, granule neurons from the hippocampus dentate gyrus region; ASC, astrocytes; MICROGLIA, microglia; OLIGO, oligodendrocytes; OPC, oligodendrocyte precursor cells; NSC, neuronal stem cells; END, endothelial cells.

For each brain tissue sample, we used FANS to collect three different cell populations. First, we used NeuN as a neuronal biomarker to capture mature neuronal nuclei. NeuN has been successfully used as a biomarker for mature postmitotic neurons in previous cell type-specific chromatin analyses (Fullard et al., 2018; Girdhar et al., 2018; Nott et al., 2019). We then used the transcription factor Pu.1 as a microglial biomarker to isolate microglial nuclei from the NeuN- population. Pu.1 is responsible for the expression of genes that drive myeloid differentiation, and has been previously used as a biomarker for microglia for cell type-specific chromatin analysis (Nott et al., 2019). Nuclei that did not stain positively for NeuN and Pu.1 were also collected as a putative glia-enriched population. An equal number of nuclei (400,000) were sorted for each cell population (NeuN+, Pu.1+, and NeuN-/Pu.1-) (Figure 1A and Supplementary Figures 4–6) (Marion-Poll et al., 2014). We performed H3K27ac ChIP sequencing on the chromatin isolated from each sorted cell population in duplicate. We assessed sample quality by calling regions of H3K27ac enrichment (peaks) for each individual sequencing sample and computing quality metrics based on standard ENCODE guidelines (Landt et al., 2012). We detected an average of 91,614 (s.d = 21,197, range = 50,662–149,681) peaks per sample. These peaks overlapped with a large fraction of total sequencing reads (mean FRiP = 0.256, s.d. = 0.136, range = 0.047–0.567), comparable to previous high quality H3K27ac profiles (Consortium et al., 2015). We further curated these samples based on normalized strand cross correlation (NSC) and relative strand cross correlation (RSC) measures to ensure that we retained the highest quality sequencing samples for all downstream analyses (Methods, Supplementary Figure 1). Based on these parameters, 20 out of a total of 159 libraries were excluded from our analysis. Information regarding read depth, duplicate reads, and other quality control information can be found for each library in Supplementary Table 2.

Across both brain region and each cell population, we used ENCODE recommended approaches (Landt et al., 2012) to call a consensus H3K27ac peak set consisting of 352,012 peaks, covering 17.7% of the hg19 genome. This peak set represented the combined H3K27ac profile of all three sorted cell populations in the dlPFC and hippocampus of individuals with and without Aβ pathology (Methods, Supplementary Table 3). We found that a majority of peaks were either intronic (59.2%) or intergenic (31.7%) and the remaining few lay in annotated exons, promoter-TSS sites, transcription termination sites, and 5′ and 3′ untranslated regions (UTRs). To look for brain enrichment, we intersected this peak set against H3K27ac peaks from 98 tissues/cell types in the Roadmap Epigenomics dataset (Consortium et al., 2015). As expected, we found that brain tissues/cell types ranked the highest in terms of jaccard index (Quinlan and Hall, 2010) for intersection with our peak set compared to other non-brain tissues/cell types (Supplementary Table 4). We computed read counts at these peaks for every sample and performed principal components analysis (PCA) using normalized read counts from the differential analysis software package DESeq2 (Love et al., 2014) to identify major sources of variation. We observed separation primarily based on cell population. For example, principal component 1 separated NeuN+ samples from NeuN- samples and explained 53% of the total variance (Supplementary Figure 7). We also applied variance partition analysis (Hoffman and Schadt, 2016; Hoffman and Roussos, 2021) which again showed that cell type population is the biggest contributor to variance in H3K27ac levels at each peak (Supplementary Figure 8). Following cell type population, we found that brain region and subject ID contribute to the most variance. Additionally, intersecting peaks from different brain regions, and cell types in a pairwise manner, we found that the peaks for each cell type displayed stronger intersections with peaks for the same cell type in the other brain region (Supplementary Table 5). This concordance in cell type between brain regions also suggests successful sorting. We note that DLPFC Pu.1+ peaks showed some overlap with hippocampus NeuN-/Pu.1- peaks which is likely due to cleaner microglia sorting in the hippocampus samples (Supplementary Table 5). We then used DESeq2 to contrast each cell type against the other two cell types to identify cell type-specific H3K27ac peaks (q < 0.05; number NeuN+-specific peaks = 160,321, Pu.1+-specific peaks = 121,558, NeuN-/Pu.1- specific peaks = 122,441).

### Active promoters and enhancers in neurons, microglia and oligodendrocyte enriched glia

To assess the efficacy of the sorting, we generated genome browser tracks of H3K27ac signal for each cell population by averaging signal across all individuals. We visualized these genome browser tracks near genes encoding the proteins used as biomarkers for neurons and microglia—*RBFOX3* which encodes NeuN, and *SPI1* which encodes Pu.1 (Figure 1B). As expected, we observed hyperacetylation near the *RBFOX3* gene in NeuN+ samples relative to Pu.1+ and NeuN-/Pu.1- samples (log2fc of peak closest to RBFOX3 promoter = 1.99, FDR q = 4.88e-107), and hyperacetylation near the SPI1 gene in Pu.1+ samples relative to NeuN+ and NeuN-/Pu.1- samples (log2fc of peak closest to SPI1 promoter = 1.8, FDR q = 8.22e-58). This suggests our sorting successfully enriched for the intended cell populations. Interestingly, compared to NeuN+ and Pu.1+ samples, we observed hyperacetylation in the NeuN-/Pu.1- samples near genes that are highly expressed in oligodendrocytes, such as *OLIG2* (log2fc of peak closest to OLIG2 promoter = 1.23, FDR q = 3.8e-52). This suggested NeuN-/Pu.1- samples were enriched for oligodendrocytes.

To further assess sorting efficacy and to better identify the cell types captured in the NeuN-/Pu.1- population, we compared our H3K27ac ChIP-seq data with an independent single nucleus gene expression (snRNA-seq) dataset from the prefrontal cortex and hippocampus of non-diseased individuals (Habib et al., 2017). As expected, the NeuN+ samples displayed significant hyperacetylation at peaks annotated to nearby genes defined to be markers of excitatory neuron clusters from prefrontal cortex (average log2fc of H3K27ac peaks annotated to excitatory PFC cluster 1 marker genes = 0.89, FDR q = 7.6e-207, average log2fc excitatory PFC cluster 2 = 0.87, FDR q = 4.7e-95), hippocampus (avg. log2fc excitatory CA1 cluster = 0.94, FDR q = 1.37e-175, avg. log_2_fc excitatory CA3 cluster = 0.75, FDR q = 4.39e-83), and dentate gyrus (avg. log2fc excitatory DG cluster = 0.65, FDR q = 7.76e-38), and also GABAergic neuron clusters (avg. log2fc GABA cluster 1 = 0.66, FDR q = 1.8e-30, avg. log2fc GABA cluster 2 = 0.66, FDR q = 4.75e-34) (Supplementary Figure 9B). Similarly, the Pu.1+ samples displayed significant hyperacetylation on average at peaks annotated to genes significantly upregulated in microglia (avg. log2fc microglia cluster = 0.64, FDR q = 1.01e-24). Strikingly, the NeuN-/Pu.1- samples displayed significant hyperacetylation at peaks annotated to genes enriched in oligodendrocyte clusters (avg. log2fc oligodendrocyte cluster 1 = 0.65, FDR q = 3.57e-61, avg. log2fc oligodendrocyte cluster 2 = 0.58, FDR q = 4.76e-28), but not any of the other cell type clusters queried, confirming oligodendrocyte enrichment.

Since AD pathology, brain region, and sex could potentially influence sample quality and sorting efficacy, we repeated this analysis separately for (i) samples with and without Aβ, (ii) samples from dlPFC and hippocampus, (iii) male and female samples, and (iv) each sample individually. In each of these analyses, we observed neuronal enrichment in NeuN+ samples, microglial enrichment in Pu.1+ samples, and oligodendrocyte enrichment in NeuN-/Pu.1- samples (Figure 1C and Supplementary Figure 9C). Since enhancers are known to have long range effects and may not necessarily regulate their nearest genes, we also restricted the analysis to peaks proximal to gene transcription start sites (TSS) (<5 kilobases) and observed the same results (Figure 1C and Supplementary Figure 9A). In addition, intersection of our cell type-specific peak sets with single nucleus ATAC-seq (snATAC-seq) data of non-diseased adult human brain (Corces et al., 2020) revealed similar cell type enrichments (Supplementary Table 6). Therefore, we conclude that the NeuN+ population successfully captures neurons, the Pu.1+ population successfully captures microglia, and the NeuN-/Pu.1- population is highly enriched for oligodendrocytes. We termed the NeuN-/Pu.1- population “oligodendrocyte enriched glia” (OEG).

Together, our peak annotations represent the first genome-wide maps of H3K27ac in microglia, neurons, and OEG from AD hippocampus and dlPFC. These annotations will enable a better understanding of the gene regulatory activity within the profiled cell types in many different contexts, not limited to AD. In the next sections, we utilize these annotations to understand cell type-specific epigenomic mechanisms in AD. First, we compare these annotations with GWAS data to annotate LOAD associated SNPs to the cell types and regulatory elements they may potentially disrupt. Second, we perform differential acetylation analysis in each sex, brain region, and cell type to identify AD-associated variations in H3K27ac. Third, we identify H3K27ac differences associated with age in each cell type.

### Interpreting cell-type specificity and potential disruptions of non-coding Alzheimer’s disease associated variants

**FIGURE 2:**
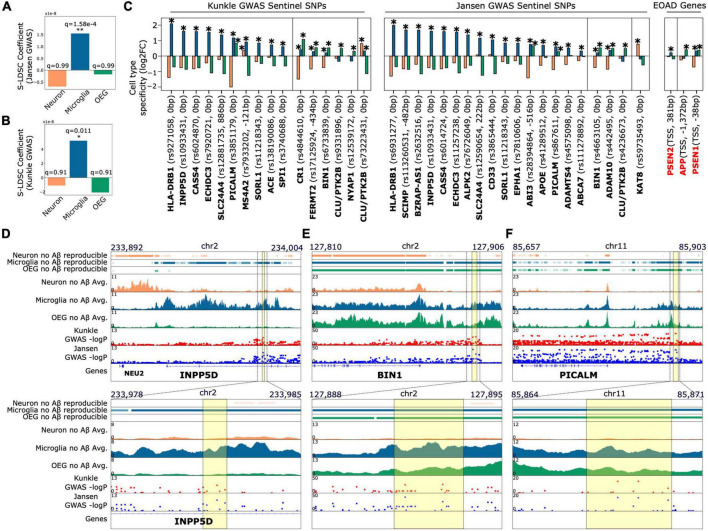
AD associated SNPs derived from GWAS prefer to colocalize with peaks enriched in the microglial population relative to peaks enriched in the OEG and neuronal populations **(A,B)**. Results of stratified LD score regression from two AD GWAS studies (Jansen et al., 2019; Kunkle et al., 2019) and cell type-specific H3K27ac peaks. Plots show the estimated LD score regression coefficient for the three peak sets. Benjamini Hochberg FDR corrected q-values across the three tests for enrichment are indicated above each bar **(C)**. Cell type enrichment of peaks annotated to sentinel SNPs at AD risk loci identified by Jansen et al. (2019) and Kunkle et al. (2019). Plots show fold change (log2-transformed) of H3K27ac signal for each population against the other two populations for (i) in black: peaks closest to the sentinel SNP at each locus associated with AD from GWAS, and (ii) in red: promoter peaks of early onset AD risk genes (*APP*, *PSEN1*, *PSEN2*). *Indicates DeSeq2 FDR q < 0.05 for the cell type-specific contrast. Sentinel SNPs that introduce missense mutations in proteins or SNPs where the closest H3K27ac peak is annotated > 1kb away are not included. This restriction was to ensure the analysis comprised only of SNPs that likely have functional effects on promoters or enhancer activity **(D–F)** top: Genome browser tracks of (i) reproducible peaks in each cell type for subjects without Aβ load, (ii) average H3K27ac signal in subjects without Aβ load for each cell type, and (iii) Manhattan plots of Jansen et al. (2019) and Kunkle et al. (2019) genetic variants. Plots are focused at loci where sentinel non-coding SNPs overlap with peaks enriched in non-neuronal cell types (d. *INPP5D*, e. *BIN1*, f. *PICALM*); bottom: zoomed in versions of the genome browser tracks displayed on top. *INPP5D* locus: the sentinel SNP rs10933431 overlaps with a peak that is enriched only in the microglial population; *BIN1* locus: the top two AD-associated SNPs based on GWAS *p*-value (rs4663105 and rs6733839) overlap with peaks enriched in both the microglial and OEG populations; *PICALM* locus: the top two SNPs (rs10792832 and rs3851179) also overlap with non-neuronal peaks. Regions of overlap are highlighted with a yellow box.

Overall, our H3K27ac peak annotations improve the interpretation of the functional effects of non-coding LOAD-associated SNPs. We point out specific examples such as the locus containing the *INPP5D* gene, where the sentinel SNP rs10933431 (GWAS *p*-values = 8.9e-10, 2.5e-07) overlaps a peak that is acetylated only in microglia but not neurons and OEG (log2fc Neuron = −0.75; log2fc Microglia = 1.61, q = 6.62e-81; log2fc OEG = −0.86) (Figure 2D). This suggests that rs10933431 may alter regulatory function in microglia and potentially other immune cell types. Future studies on the functional effect of this SNP should include culture or model systems that can capture phenotypes of these cell types. Secondly, at the locus containing the *BIN1* gene, which displays the second largest genome wide AD association behind the *APOE* locus, two sentinel SNPs, rs4663105 (GWAS *p*-values = 3.37e-44, 2.16e-26) and rs6733839 (GWAS *p*-values = 1.28e-29, 4.02e-28), overlap with a peak significantly enriched in both microglia and OEG, but not neurons (log2fc Neuron = −0.74; log2fc Microglia = 0.21, q = 2.66e-3; log2fc OEG = 0.53, *q* = 2.11e-15) (Figure 2E). This suggests that studies to test the effects of rs6733839 or rs4663105 on *BIN1* expression should be conducted in oligodendrocytes in addition to cells of the myeloid lineage (Nott et al., 2019). Similarly, at the locus containing *PICALM*, one sentinel SNP, rs10792832 (GWAS *p*-values = 7.36e-18, 7.55e-16) and another SNP in tight linkage, rs3851179 (GWAS *p*-values = 2.02e-17, 5.81e-16) overlap with microglia and OEG H3K27ac peaks (log2fc Neuron = −2.0; log2fc Microglia = 1.17, FDR *q* = 4.09e-41; log2fc OEG = 0.83, FDR *q* = 7.34e-22) (Figure 2F). These examples highlight the utility of our data as a resource for informing future studies of non-coding SNPs associated with traits that include, but are not limited to AD.

### Genome wide association studies derived common single nucleotide polymorphisms associated with late onset sporadic Alzheimer’s disease risk preferentially colocalize with microglial H3K27ac

We performed partitioned heritability analysis by stratified LD score regression (Bulik-Sullivan et al., 2015; Finucane et al., 2015, 2018) (S-LDSC) to estimate the genome wide strength of colocalization between cell type-specific H3K27ac peaks and AD SNP heritability. We assessed AD SNP heritability from two large AD GWAS meta analyses (Jansen et al., 2019; total observed scale heritability = 0.0095; s.e. = 0.0021, and Kunkle et al., 2019, total observed scale heritability = 0.0534; s.e = 0.01). We found that microglia-specific peaks displayed a statistically significant preference for colocalization with AD associated SNPs (Figures 2A,B; Jansen et al., 2019, GWAS coefficient = 1.6e-08, FDR *q* = 1.58e-4, observed scale heritability = 0.0035, s.e. = 0.0027; Kunkle et al., 2019, GWAS coefficient = 1.94e-08, FDR *q* = 0.011, observed scale heritability = 1.64e-02, s.e. = 0.012) relative to neuronal and OEG-specific peaks. Since choice of computational method can influence these assessments, we repeated the analysis with an independent method that utilizes a permutation test (Consortium et al., 2015; Gjoneska et al., 2015). We again observed that AD SNP heritability has a strong preference for colocalization with microglia-specific peaks (Supplementary Figure 10; Kunkle log2FC = +0.39, FDR *q* = 3e-6, Jansen log2FC = +0.33, FDR *q* = 3e-6). Additionally, we conducted S-LDSC analysis with all acetylated peaks in each cell type (as opposed to the cell type-specific peaks derived from DESeq2 cell type contrasts). These were obtained by running the ENCODE ChIP-seq pipeline to call reproducible peaks (Li et al., 2011) across all control (no Aβ) samples for each of the 3 cell type populations (number reproducible NeuN+ peaks = 215,929, reproducible Pu.1+ peaks = 174,123, reproducible NeuN-/Pu.1- peaks = 154,562). Only control subjects were used in this analysis to avoid including or excluding peaks associated with AD. This S-LDSC analysis with reproducible peaks yielded similar results (Jansen et al., 2019, GWAS coefficient for microglia = 1.05e-8, FDR *q* = 7.9e-4, observed scale heritability = 0.01, s.e. = 0.0031; Kunkle et al., 2019, GWAS coefficient for microglia = 4.28e-8, FDR *q* = 0.019, observed scale heritability = 7.15e-2, s.e. = 0.0166). These findings agree with previous analyses conducted on myeloid cells (Gjoneska et al., 2015; Huang et al., 2017; Keren-Shaul et al., 2017), reinforcing the hypothesis that myeloid cell gene regulation strongly influences AD predisposition.

We note that neuron-specific peaks overlap with a lower number (log2fc from permutation test approach = −0.38) of GWAS derived AD associated SNPs compared to microglia and OEG-specific peaks (Supplementary Figure 10 and Figure 2C). This finding is consistent with previous analyses conducted on bulk brain tissue histone modification profiles (Consortium et al., 2015; Gjoneska et al., 2015) and open chromatin (Gosselin et al., 2017; Lake et al., 2017), where signals from neuronal regulatory elements are dominant. Since biases in GWAS sampling and neuronal sample quality could potentially influence the results of these analyses, we performed a positive control S-LDSC analysis to partition Schizophrenia SNP heritability (total observed scale heritability = 0.4103, s.e. = 0.0186) (Consortium Swg of the PG, 2014) across each set of cell type-specific peaks. As expected, only neuron-specific peaks displayed significant colocalization (Supplementary Figure 11; coefficient = 1.5e-07, FDR *q* = 4.2e-8, observed scale heritability = 4.84e-2, s.e. = 0.0254). This agrees with previous findings regarding neuron-specific open chromatin in Schizophrenia (Fullard et al., 2017, 2018), and therefore confirms that our analysis is robust to biases in GWAS sampling and cell type sample quality.

Lastly, we annotated all non-coding sentinel SNPs identified in Jansen et al. (2019) and Kunkle et al. (2019) that may influence gene regulatory activity at promoters and enhancers (<1 kb distance between SNP and nearest peak, and SNP is not a missense mutation in a protein) to nearby H3K27ac peaks (Figure 2C and Table 1). For each variant, this enables the identification of potential cell types in which they may alter gene regulatory activity. As expected, at a majority of GWAS derived risk loci, the sentinel SNPs directly overlapped with H3K27ac peaks that are significantly enriched in microglia (avg. log2fc Microglia = 0.81; avg. log2fc OEG = −0.34; avg. log2fc Neuron = −0.47). Interestingly, sentinel SNPs at 8 loci, including those containing genes *BIN1*, *CLU*, *ADAM10*, *NYAP1*, and *CR1*, directly overlap with H3K27ac peaks that are significantly enriched in OEG (avg. log2fc OEG = 0.35; avg. log2fc Microglia = 0.06; avg. log2fc Neuron = −0.414). Only 2 sentinel SNPs, near *CLU* (log2fc Neuron = 0.80, FDR *q* = 1.94e-31; log2fc Microglia = 0.33, FDR *q* = 4.21e-6; log2fc OEG = −1.14) and *KAT8* (log2fc Neuron = 0.77, *q* = 4.72e-13; log2fc Microglia = −0.19; log2fc OEG = −0.58), overlapped with peaks that are significantly enriched in neurons. In addition to GWAS derived risk loci, we were interested to see if H3K27ac peaks associated with familial AD genes displayed any cell type-specific enrichment. Interestingly, the peaks closest to the TSS of *APP* (log2fc Neuron = −0.25; log2fc Microglia = −0.15; log2fc OEG = 0.41, *q* = 5.23e-10) and *PSEN1* (log2fc Neuron = −0.71; log2fc Microglia = 0.33, FDR *q* = 2.59e-6; log2fc OEG = 0.38, *q* = 1.59e-8) displayed significant enrichment in OEG, whereas the peak closest to the TSS of *PSEN2* (log2fc Neuron = −0.03; log2fc Microglia = 0.22, *q* = 7.7e-4; log2fc OEG = −0.18) displayed significant enrichment in microglia.

**TABLE 1:** Cell type specificity of peaks closest to sentinel SNPs at previously identified genome-wide significant loci from two different GWAS.

Gene locus	CHR	Lead SNP	BP	*P*-value (overall)	GWAS study	Closest peak distance	Peak start	Peak end	log2FC (Neuron vs. other two)	log2FC (microglia vs. other two)	log2FC (OEG vs. other two)
APOE	chr19	rs41289512	45351516	5.79E-276	Jansen	0	45347500	45352552	–0.599051558	0.702466985	–0.103402899
BIN1	chr2	rs6733839	127892810	2.10E-44	Kunkle	0	127824833	127857093	–0.744462694	0.211415139	0.533064808
BIN1	chr2	rs4663105	127891427	3.38E-44	Jansen	0	127824833	127857093	–0.744462694	0.211415139	0.533064808
PICALM	chr11	rs3851179	85868640	6.00E-25	Kunkle	0	85865576	85869702	–2.003772477	1.175357699	0.828425616
CR1	chr1	rs4844610	207802552	3.60E-24	Kunkle	0	207800766	207803241	–1.496538907	0.411639699	1.084907206
CLU/PTK2B	chr8	rs9331896	27467686	4.60E-24	Kunkle	0	27464855	27473351	–0.146423136	–0.342092698	0.488530519
CLU/PTK2B	chr8	rs4236673	27464929	2.61E-19	Jansen	0	27464855	27473351	–0.146423136	–0.342092698	0.488530519
PICALM	chr11	rs867611	85776544	2.19E-18	Jansen	0	85773701	85782052	–0.878318768	0.59296884	0.285364043
TREM2	chr6	rs75932628	41129252	2.70E-15	Kunkle	0	41123195	41131918	–1.3555633	2.011559515	–0.655985632
CLU/PTK2B	chr8	rs73223431	27219987	6.30E-14	Kunkle	0	27216401	27226944	0.804019973	0.334689571	–1.138697236
SPI1	chr11	rs3740688	47380340	5.40E-13	Kunkle	0	47379638	47380774	0.036871997	0.607042966	–0.643907383
SORL1	chr11	rs11218343	121435587	2.90E-12	Kunkle	0	121434568	121439029	–0.360132993	0.8441546	–0.484011059
SORL1	chr11	rs11218343	121435587	1.09E-11	Jansen	0	121434568	121439029	–0.360132993	0.8441546	–0.484011059
HLA-DRB1	chr6	rs9271058	32575406	1.40E-11	Kunkle	0	32575148	32577842	–1.386968687	2.093033774	–0.706057504
EPHA1	chr7	rs7810606	143108158	3.59E-11	Jansen	0	143106905	143108283	–0.303851955	0.835592345	–0.531732978
ABCA7	chr19	rs111278892	1039323	7.93E-11	Jansen	0	1038908	1043234	–0.296373211	0.332547828	–0.036162888
HLA-DRB1	chr6	rs6931277	32583357	8.41E-11	Jansen	0	32582269	32584446	–1.301789562	1.992085151	–0.690289688
ADAMTS4	chr1	rs4575098	161155392	2.05E-10	Jansen	0	161148711	161156367	–0.549341105	0.511794299	0.037559007
CASS4	chr20	rs6014724	54998544	6.56E-10	Jansen	0	54994953	54999360	–0.82514278	1.586379997	–0.761227739
NYAP1	chr7	rs12539172	100091795	9.30E-10	Kunkle	0	100085037	100088295	0.016179468	–0.325104173	0.308936027
ADAM10	chr15	rs442495	59022615	1.31E-09	Jansen	0	59020947	59024537	–0.854287247	0.363816841	0.49048051
ECHDC3	chr10	rs7920721	11720308	2.30E-09	Kunkle	0	11714504	11720725	–0.443367527	1.535382419	–1.092004912
INPP5D	chr2	rs10933431	233981912	3.40E-09	Kunkle	0	233976966	233984034	–0.751579648	1.610757377	–0.859166938
CD33	chr19	rs3865444	51727962	6.34E-09	Jansen	0	51727712	51728032	0.09666484	1.050643064	–1.147304253
ACE	chr17	rs138190086	61538148	7.50E-09	Kunkle	0	61538112	61538580	–0.090757218	0.725122365	–0.634360388
ECHDC3	chr10	rs11257238	11717397	1.26E-08	Jansen	0	11714504	11720725	–0.443367527	1.535382419	–1.092004912
ALPK2	chr18	rs76726049	56189459	3.30E-08	Jansen	0	56193436	56194075	–0.772160797	1.372526359	–0.600361544
APH1B	chr15	rs117618017	63569902	3.35E-08	Jansen	0	63567370	63571899	–0.182293607	0.063658874	0.118647308
CASS4	chr20	rs6024870	54997568	3.50E-08	Kunkle	0	54994953	54999360	–0.82514278	1.586379997	–0.761227739
KAT8	chr16	rs59735493	31133100	3.98E-08	Jansen	0	31132717	31133297	0.769923745	–0.192635059	–0.577283985
BZRAP-AS1	chr17	rs2632516	56409089	9.66E-07	Jansen	0	56409045	56411269	–0.534067259	1.685675299	–1.151598432
INPP5D	chr2	rs10933431	233981912	8.92E+10	Jansen	0	233976966	233984034	–0.751579648	1.610757377	–0.859166938
ZCWPW1	chr7	rs1859788	99971834	2.22E-15	Jansen	74	99971908	99972977	–0.587916605	1.520171864	–0.932247137
MS4A2	chr11	rs7933202	59936926	1.90E-19	Kunkle	-121	59936521	59936805	0.349562941	0.908767826	–1.258327617
SLC24A4	chr14	rs12590654	92938855	1.65E-10	Jansen	222	92939077	92940753	0.076299566	1.166870187	–1.243161989
APOE	chr19	rs429358	45411941		Kunkle	276	45412217	45412618	–0.685919297	0.70439705	–0.018472744
FERMT2	chr14	rs17125924	53391680	1.40E-09	Kunkle	-434	53390146	53391246	–0.835642458	0.288762577	0.546886874
SCIMP	chr17	rs113260531	5138980	9.16E-10	Jansen	-482	5135305	5138498	–0.82789803	1.691000283	–0.863092835
ABI3	chr17	rs28394864	47450775	1.87E-08	Jansen	-516	47449704	47450259	–1.416013571	0.750716835	0.665301832
SLC24A4	chr14	rs12881735	92932828	7.40E-09	Kunkle	886	92933714	92934206	–0.230512734	1.371875261	–1.141358708
EPHA1	chr7	rs10808026	143099133	1.30E-10	Kunkle	3136	143102269	143103054	–0.214105144	0.150370138	0.063740851
HESX1	chr3	rs184384746	57226150	1.24E+08	Jansen	4299	57230449	57230985	0.803339853	0.102926026	–0.906261534
ABCA7	chr19	rs3752246	1056492	3.10E-16	Kunkle	7116	1063608	1065445	–0.661452848	1.248270475	–0.586810045
CR1	chr1	rs2093760	207786828	1.10E-18	Jansen	-1966	207784517	207784862	–1.415642369	0.445194153	0.970451535
CD2AP	chr6	rs9473117	47431284	1.20E-10	Kunkle	-1984	47428686	47429300	–1.493938259	1.868936054	–0.37499374
MS4A6A	chr11	rs2081545	59958380	1.55E-15	Jansen	-2258	59955591	59956122	–1.745958809	2.242252117	–0.496290856
CD2AP	chr6	rs9381563	47432637	2.52E-10	Jansen	-3337	47428686	47429300	–1.493938259	1.868936054	–0.37499374
CNTNAP2	chr7	rs114360492	145950029	2.10E-09	Jansen	-17047	145931797	145932982	1.397706583	–0.880179139	–0.517521709
HS3ST1	chr4	rs7657553	11723235	0.051	Jansen	-18278	11704552	11704957	0.998279239	–0.566853392	–0.431422538
TREM2	chr6	rs187370608	40942196	1.45E-16	Jansen	21690	40963886	40965725	0.626190634	–0.352810161	–0.273372262
CLNK	chr4	rs6448453	11026028	1.93E-09	Jansen	-39655	10984912	10986373	–0.7967249	1.351333931	–0.554603351

### Aβ associated acetylation differences in oligodendrocyte enriched glia

**FIGURE 3:**
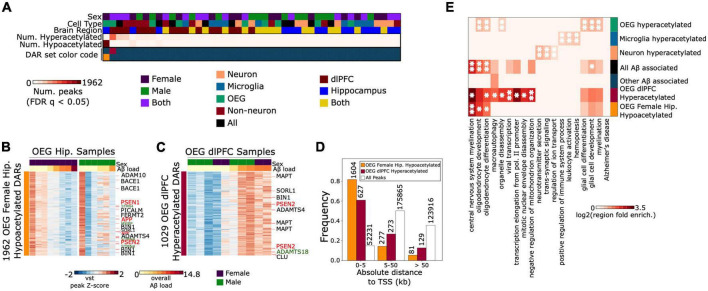
OEG display the strongest acetylation differences associated with Aβ pathology, including peaks annotated to genes associated with EOAD and LOAD risk **(A)**. Heatmap displaying number of significantly hyperacetylated (log2fc > 0, FDR q < 0.05) and significantly hypoacetylated peaks (log2fc < 0, FDR q < 0.05) from each brain region, sex, and cell type-specific contrast **(B)** left: Heatmap of normalized acetylation levels at 1962 H3K27ac peaks that were significantly hypoacetylated in AD female hippocampus OEG samples. Rows represent the 1,962 DARs and columns represent hippocampal OEG samples. Aβ load for each sample is indicated at the top of the heatmap. Right: A heatmap of the 1,962 peaks in male hippocampal OEG samples is included for comparison. DARs annotated to EOAD and LOAD risk genes are labeled in red and black, respectively. Peaks near *STMN4* and *MYRF* are annotated in green **(C)**. Heatmap of the 1,029 H3K27ac peaks that were significantly hyperacetylated in AD dlPFC OEG samples. Peaks annotated to EOAD and LOAD risk genes are labeled in red and black, respectively. The *ADAMTS18* promoter-proximal peak is annotated in green **(D)**. Distance to TSS distribution of (i) 1,962 OEG female hippocampus hypoacetylated DARs, (ii) 1,029 OEG dlPFC hyperacetylated DARs and (iii) the full consensus set of peaks **(E)**. Enrichment heatmap of top gene ontology terms for 6 peak sets (1) 1,962 OEG female hippocampus hypoacetylated DARs (2) 1,029 OEG dlPFC hyperacetylated DARs (3) all other Aβ associated DARs (4) neuron, (5) microglia, and (6) OEG cell type-specific hyperacetylated peaks. Color intensity represents hypergeometric fold enrichment in number of peaks over background (full consensus peak set), *indicates FDR *q* < 0.05, ^**^indicates FDR *q* < 0.01.

To characterize the cell type-specific H3K27ac changes associated with Aβ pathology, we performed a series of differential analyses using DESeq2 (Love et al., 2014). Aβ vs. no-Aβ contrasts were performed for each brain region, sex, and cell type. All contrasts performed, results of contrasts, and number of samples included in each contrast, are described in Supplementary Table 7. When combining all Aβ-associated DARs from these contrasts, we identified a total of 3,598 Aβ-associated differentially acetylated regions (DARs) (FDR *q* < 0.05) (Supplementary Table 7 and Figure 3A). Due to the strong enrichment of microglial H3K27ac near AD risk loci, we expected this set to be dominated by peaks with robust acetylation differences between the Aβ vs. non-Aβ microglia samples. Unexpectedly, however, Aβ-associated DARs in microglia formed a minority of all DARs (85 peaks total, 2.4%). In contrast, OEG were associated with the majority of DARs (2,991 peaks total, 80.3%). This included a set of 1,962 hypoacetylated DARs identified from the female-specific hippocampus OEG contrast, and another set of 1,029 hyperacetylated DARs identified from the dlPFC OEG contrast including both sexes. Both of these DAR sets displayed progressive trends in acetylation when treating Aβ load as a continuous variable (Supplementary Figures 12B, 13B). Furthermore, in a *post hoc* analysis, we tested for correlations with other variables such as age at death, postmortem interval, years of education, and RSC (Supplementary Figures 12, 13). None of the DARs were correlated with age, years of education, or pmi (FDR *q* < 0.05). 469 of 1,962 (23.9%) OEG female-specific hippocampus DARs were correlated with RSC (FDR *q* < 0.05), and 975 out of 1,029 (94.3%) OEG dlPFC DARs were correlated with RSC (FDR *q* < 0.05). However, log2fc effect sizes for Aβ when covarying RSC and Aβ (**RSC + Aβ**) remained correlated with log2fc values from the original model (**Aβ** only) [Supplementary Table 8; Pearson’s r for 1,962 OEG female hippocampus DARs = 0.73, *p* = 1.5e-323; Pearson’s r for 1,029 OEG dlPFC DARs = 0.77, *p* = 1.5e-207)]. Additionally, this differential analysis was performed on a curated peak set that had already passed NSC and RSC quality control thresholds, ensuring the exclusion of low-quality reads (Methods). In the next sections, we describe these two DAR sets in more detail.

### Hypoacetylation in hippocampal oligodendrocyte enriched glia and corresponding gene expression changes in positively sorted oligodendrocytes

We identified 1,962 hypoacetylated DARs in female Aβ hippocampus OEG samples, 81.7% of which were peaks proximal to TSS (< 5 kb) (hypergeometric test *p*-value = 0, Figure 3D), suggesting strong links with promoter activity and gene transcription. To identify the biological pathways associated with this DAR set, we performed gene ontology enrichment analysis using Genomic Region Enrichment and Annotation Tool (GREAT) (McLean et al., 2010). This analysis revealed an enrichment for central nervous system myelination (region fold enrichment = 3.95, FDR *q* = 8.8e-3), oligodendrocyte development (region fold enrichment = 2.88, FDR *q* = 1.7e-2), and oligodendrocyte differentiation (region fold enrichment = 2.22, FDR *q* = 2.4e-2) (Supplementary Table 9 and Figure 3E). We also observed hypoacetylation near genes in the KEGG Alzheimer’s Disease pathway, including those encoding the five mitochondrial complexes that regulate oxidative phosphorylation (Supplementary Figure 14). To confirm that we were not simply enriching for oligodendrocyte signal in this analysis, we conducted secondary gene ontology enrichment analysis with oligodendrocyte-specific backgrounds. The two new backgrounds we used were peaks that were specifically enriched in hippocampal OEG relative to the other 2 populations, and separately, peaks that were specifically enriched in OEG relative to the other 2 populations across both profiled brain regions (Methods). Enrichment for central nervous system myelination (hippocampus background *p* = 2.3e-4, region fold enrichment = 2.55; both background *p* = 5.89e-5, region fold enrichment = 2.84), oligodendrocyte development (hippocampus background *p* = 6.13e-3, region fold enrichment = 1.72; both background *p* = 1.83e-3, region fold enrichment = 1.89), and oligodendrocyte differentiation (hippocampus background *p* = 2.5e-2, region fold enrichment = 1.4; both background *p* = 5.99e-3, region fold enrichment = 1.54) were still detectable.

We found the strongest hypoacetylation in this DAR set at a peak annotated to the *STMN4* gene (log2FC = −1.12, FDR *q* = 1e-6) which is preferentially expressed in brain tissue (Fagerberg et al., 2014) and has known functions in neuron projection development and microtubule polymerization (Gaudet et al., 2011). Notably, multiple other peaks near the *STMN4* gene, including a peak at the *STMN4* promoter, displayed significant hypoacetylation (log2fc = −0.57, FDR *q* = 0.019). *MYRF*, a transcription factor which directly activates myelination (Bujalka et al., 2013) and has been previously linked to LOAD risk (Vardarajan et al., 2018), also displayed strong promoter hypoacetylation (log2FC = −0.48, FDR *q* = 0.03). Aβ is known to be toxic to oligodendrocytes, affecting basic processes such as myelination (Desai et al., 2010). Neuroinflammation associated with neurodegeneration is also known to disrupt myelin (Peferoen et al., 2014). Thus, these hypoacetylated peaks may reflect myelinating processes dysregulated in AD.

This hypoacetylated DAR set also included peaks at the promoters of *APP* (log2fc = −0.38, FDR *q* = 0.045), *PSEN1* (log2fc = −0.41, FDR *q* = 0.046), and *PSEN2* (log2fc = −0.42, FDR *q* = 0.049). Additionally, the promoters of genes involved in all three secretase complexes including α-secretase (*ADAM10*), (log2fc = −0.44, FDR *q* = 0.037), β-secretase (*BACE1*), (log2fc = −0.49, FDR *q* = 0.032), and γ-secretase (*PSEN1*, *PSEN2*) were hypoacetylated. Further, the protein-protein interaction (PPI) networks (Szklarczyk et al., 2021) generated from genes annotated to this DAR set revealed clusters associated with amyloid processing (Supplementary Figure 15).

While the majority of Aβ species are produced by neurons, oligodendrocytes have also previously been shown to produce Aβ (Skaper et al., 2009). Interestingly, *BACE1* is also known to regulate myelination (Hu et al., 2006), suggesting hypoacetylation of these EOAD risk genes may be tied to the dysregulated oligodendrocyte processes which were also observed in hippocampal OEG. The oligodendrocyte-specific function of these risk genes must be further interrogated to interpret these results further.

**FIGURE 4:**
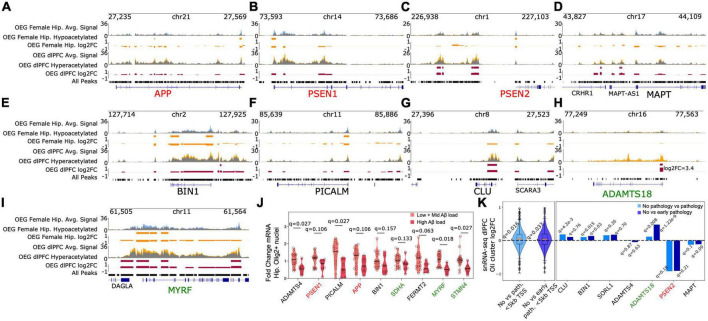
EOAD and LOAD risk genes exhibit epigenomic and transcriptomic perturbations in oligodendrocytes **(A–I)**. Genome browser tracks displaying average H3K27ac signal in OEG samples from subjects with and without Aβ load (yellow and blue tracks, respectively). Regions displayed include EOAD and LOAD risk loci, as well as differentially acetylated regions near *ADAMTS18* and *MYRF* **(J)** RT-qPCR of select genes annotated to DARs identified in AD OEG female hippocampus. Panel shows violin plots of gene expression measured by RT-qPCR in hippocampal Olig2+ nuclei collected from an independent cohort of AD and non-AD subjects. q-values for differential expression between high Aβ and low+mid Aβ subjects are indicated on top for each gene. Correction was applied across the 9 tests **(K)** left panel: comparison with existing snRNA-seq from AD dlPFC (Mathys et al., 2019) reveals an average increase in gene expression near hyperacetylated regions in OEG dlPFC. Violin plots depict log2fc values from differential expression analysis between AD and non-AD subjects in oligodendrocytes (Mathys et al., 2019). These log2fc values are derived from 500 genes annotated to the OEG dlPFC hyperacetylated DARs that reside in putative promoters (<5kb from TSS). Log2fc violin plots are shown for two different contrasts performed in Mathys, Valderrain et al.: no pathology vs. pathology and no pathology vs. early pathology. *Q*-values from *t*-tests (null hypothesis: mean log2fc = 0, alternate hypothesis: mean log2fc > 0) are reported for the two violin plots. Correction was applied across the two tests. Right panel: Specific genes associated with OEG dlPFC hyperacetylated DARs display increased transcription in AD. Individual log2fc values are shown. TSS distance cutoffs were not used for this right panel. FDR *q*-values from the differential expression analysis for each gene are also provided for both contrasts.

In addition to EOAD risk genes, the promoters of several LOAD risk genes were also hypoacetylated. This included *BIN1* (log2fc = −0.46, FDR *q* = 0.026), *PICALM* (log_2_fc = −0.40, FDR *q* = 0.034), *ADAMTS4* (log2fc = −0.46, FDR q = 0.038), *ADAM10*, and *FERMT2* (log2fc = −0.47, FDR *q* = 0.03) (Supplementary Table 8, Supplementary Figure 16, and Figures 3B, 4A–I). Notably, the sentinel SNPs associated with many of these genes (*BIN1*, *PICALM, ADAMTS4*, *FERMT2*) were located in H3K27ac peaks significantly enriched in OEGs relative to other cell types (Figure 2B). This raises the possibility that these SNPs could alter oligodendrocyte Aβ production or the oligodendroglial response to Aβ, which as previously mentioned is toxic to oligodendrocytes.

Previous bulk epigenomic profiling has shown H3K27ac deregulation at both EOAD and LOAD risk loci, suggesting shared pathogenic mechanisms between the two forms of AD (Marzi et al., 2018). Deregulation of H3K27ac at EOAD and LOAD risk genes in our OEG data suggests that oligodendrocytes are implicated in these shared mechanisms. However, we note that the sample size used for this analysis was limited (female-specific, Aβ *n* = 5, no- Aβ *n* = 3). Therefore, additional experimentation supporting these findings are required before they can be interpreted further. Additionally, we did not observe significant colocalization of this DAR set with GWAS signal relative to the full consensus set of peaks in an S-LDSC analysis (Jansen coefficient = 3.9e-08, *p* = 0.198, observed scale heritability = 9.51e-4, s.e. = 0.0009; Kunkle coefficient = 1.11e-07, *p* = 0.29, observed scale heritability = 0.0051, s.e. = 0.0036).

To determine if these acetylation changes were associated with altered transcription, we performed quantitative RT-PCR (RT-qPCR) on RNA isolated from positively sorted human hippocampal Olig2+ oligodendrocyte nuclei (Supplementary Figures 17, 18). These hippocampal samples (12 with Aβ load, 12 without Aβ load) were from the same ROSMAP cohort as those used for H3K27ac ChIP sequencing, and were matched for Aβ load between male and female subjects (female Aβ load mean = 10.2, s.d. = 4.21, male Aβ load mean = 9.87, s.d. = 4.41) (Methods, Figure 4J and Supplementary Figure 19). To isolate oligodendrocyte nuclei, we used our FANS protocol (Methods). We chose 9 genes to measure with qRT-PCR. Of these genes, *ADAMTS4* (Hedge’s *g* = 1.346, *q* = 0.027), and *PICALM* (*g* = 1.235, *q* = 0.027) displayed significant decreases in transcript levels when comparing low (Aβ load mean = 0.04, s.d. = 0.09) and mid-Aβ load subjects (Aβ load mean = 6.98, s.d. = 1.27) against high Aβ load subjects (Aβ load mean = 13.09, s.d. = 3.29), in agreement with the direction of acetylation differences. *STMN4* and *MYRF* also displayed significantly reduced transcription (*g* = 1.228, *q* = 0.027; *g* = 1.567, *q* = 0.018). However, the other genes tested were no longer significant when corrected for multiple testing, indicating future additional validation is needed.

These DARs were not significantly hypoacetylated in male Aβ hippocampal OEG H3K27ac samples. However, we note that Aβ load differed between male and female hippocampal H3K27ac samples, with females having higher Aβ load than males (female Aβ load mean = 8.45, s.d. = 4.91, male Aβ load mean = 4.31, s.d. = 1.83). This, along with the modest sample size, may account for the lack of coherence across sexes in detected acetylation changes. However, differences in transcription were detectable across both male and female samples in RT-qPCR analysis. Additionally, modeling of OEG hippocampus H3K27ac in DESeq2 using an interaction term between sex and Aβ load retrieved very few differential peaks for the interaction term (number of DARs = 3), of which, only one was part of the set of 1962 originally identified DARs. In total, this suggests that the acetylation and transcriptional differences are unlikely to be sex-specific, although this must be interrogated further in a larger cohort.

We also found that these DARs are enriched for a large number transcription factor binding motifs. Most interestingly, multiple members of the Sox family of transcription factors which are involved in central nervous system myelination (Wittstatt et al., 2019) are enriched. Sox factors share lot of similarity in their binding motifs so it is unclear which factor may be the master regulator that leads to these observed differences. Strikingly, we found that peaks annotated to SOX2, SOX4, and SOX5 genes are part of this DAR set suggesting that one or more of these three genes may serve as master regulators which lead to these hippocampus OEG changes. A full set of motif enrichments is available in our data upload.

### Hyperacetylation in dorsolateral prefrontal cortex oligodendrocyte enriched glia and corresponding gene expression changes in single nucleus gene expression profiled oligodendrocytes

We identified an additional DAR set in dlPFC Aβ OEG samples of both sexes. 49.1% of these DARs were distal to TSS, suggesting they may play a role in enhancer-mediated gene regulation. Gene ontology enrichment analysis of this DAR set revealed a similar enrichment for central nervous system myelination (region fold enrichment = 5.15, FDR *q* = 1.03e-2), and oligodendrocyte differentiation (region fold enrichment = 2.61, FDR q = 2.4e-2) (Figure 3E and Supplementary Table 9). In addition, we detected enrichment for negative regulation of mitochondrion organization (region fold enrichment = 3.32, FDR *q* = 6.9e-3), macroautophagy (region fold enrichment = 2.4, FDR *q* = 1.01e-2) and viral transcription (region fold enrichment = 2.89, FDR q = 9.27e-3). To confirm that we were not enriching for oligodendrocyte signal, we conducted a secondary analysis with an OEG-specific background derived from both brain regions and an OEG-specific background derived from dlPFC. Enrichment for central nervous system myelination (dlPFC background *p* = 4.46e-5, region fold enrichment = 3.86; both background *p* = 6.86e-5, region fold enrichment = 3.7), and oligodendrocyte differentiation (dlPFC background *p* = 2.75e-3, region fold enrichment = 1.89; both background *p* = 4.6e-3, region fold enrichment = 1.81) was still detectable (Gurses et al., 2016). Interestingly, the protein-protein interaction (PPI) networks (Szklarczyk et al., 2021) generated from genes annotated to this DAR set also revealed clusters associated with central nervous system myelination and oligodendrocyte differentiation (Supplementary Figure 20).

Interestingly, while this DAR set was distinct from the DARs identified in female hippocampus OEG samples, we observed acetylation changes at similar loci (Supplementary Table 8, Figures 3C, 4A–I, and Supplementary Figure 15). This again included significant hyperacetylation at both EOAD and LOAD risk loci, such as four distal intergenic peaks (>5 kb distance from any annotated TSS and not in a gene body) annotated to *PSEN2* (log2fc = 0.54, FDR *q* = 0.037; log2fc = 0.60, FDR *q* = 0.025; log2fc = 0.47, FDR *q* = 0.044; log2fc = 0.51, FDR *q* = 0.044), one distal peak annotated to *BIN1* (log2fc = 0.44, FDR *q* = 0.049), and putative promoter peaks overlapping the TSS of *CLU* (log2fc = 0.49, FDR *q* = 0.04), *ADAMTS4* (log2fc = 0.48, FDR *q* = 0.047), and *SORL1* (log2fc = 0.52, FDR *q* = 0.048). Furthermore, we observed significant hyperacetylation at three distal peaks (>5 kb from TSS) annotated to the *MAPT* gene (log2fc = 0.67, FDR *q* = 0.02; log2fc = 0.39, FDR *q* = 0.048; log2fc = 0.52, FDR *q* = 0.044), which encodes for the tau protein involved in formation of NFTs. We also observed PPI clusters associated with amyloid processing in our analysis (Supplementary Figure 20). Combined with our findings from hippocampal OEG, these results support the hypothesis that oligodendrocytes are implicated in shared EOAD and LOAD pathogenic mechanisms. However, we reiterate that the modest sample size of our cohort requires our findings to be supported by additional independent experimentation. Similar to the hippocampus OEG DAR set, we did not observe significant colocalization with GWAS derived AD-associated SNPs in S-LDSC analysis (Jansen GWAS coefficient = −2.57e-08, *p* = 0.70, observed scale heritability = −7.64e-4, s.e. = 0.0006; Kunkle GWAS coefficient = −1.26e-07, *p* = 0.74, observed scale heritability = −0.0027, s.e. = 0.0026). Therefore, SNPs associated with AD are unlikely to alter the regulatory function of these DARs directly.

We tested whether these acetylation differences are associated with differences in transcription by comparing them with a previously published AD dlPFC snRNA-seq dataset (Mathys et al., 2019). On average, genes annotated to these DARs (*n* = 500 genes that were detectable in the snRNA-seq study) displayed higher transcription levels in oligodendrocytes of subjects with AD compared to subjects without AD, in agreement with the direction of acetylation differences (no pathology vs. pathology mean log2fc = 0.036, *t*-test FDR q = 0.016; no pathology vs. early pathology mean log2fc = 0.032, *t*-test FDR *q* = 0.033) (Figure 4K). Also, we observed an enrichment in overlap between the genes annotated to these DARs and the genes that were differentially expressed (FDR q < 0.05) in oligodendrocytes of subjects with AD in the snRNA-seq dataset (no pathology vs. early pathology hypergeometric *p* = 2.8e-4, no pathology vs. pathology *p* = 2.1e-4). Individual genes associated with LOAD risk including *CLU* (log2fc no vs. path = 0.18, FDR *q* = 4.2e-3; log2fc no vs. early path. = 0.1, *q* = 0.76) and *BIN1* (log2fc no vs. path. = 0.1, *q* = 0.015, log2fc no vs. early path. = 0.13, *q* = 0.03) displayed statistically significant upregulation. *SORL1* (log2fc no vs. path. = 0.15, *q* = 0.26; log2fc no vs. early path. = 0.17, *q* = 0.70) displayed upregulation but it was not statistically significant. We note that *PSEN2* (log2fc no vs. path. = −0.92, *q* = 0.18; log2fc no vs. early path. = −0.915, FDR *q* = 0.21) and *MAPT* (log2fc no vs. path. = −0.13, *q* = 0.1; log2fc no vs. early path. = −0.116, *q* = 0.09) displayed reduced transcription with AD pathology, although this was not statistically significant. *ADAMTS18* also displayed a statistically significant increase in transcription in dlPFC oligodendrocytes of AD subjects (log2fc no vs. path. = 0.113, *q* = 0.008; log2fc no vs. early path. = 0.48, *q* = 1.22e-9) suggesting the acetylation differences are correlated with transcriptional differences.

Again, we found that these DARs are enriched for a large number transcription factor binding motifs including multiple members of the Sox family of transcription factors. In this case, however, we found that peaks annotated to SOX8 and SOX10 genes are part of this DAR set suggesting that one of these three genes may serve as master regulators which lead to these dlPFC OEG changes. A full set of motif enrichments is available on our data upload.

Overall, we reveal that pathways associated with both early and late onset AD are perturbed at the epigenomic level in OEG. We show that amyloid processing, central nervous system myelination and oligodendrocyte processes are altered in hippocampus and dlPFC of subjects with amyloid pathology. Furthermore, we find that transcription differences correlated with acetylation differences near AD risk loci, although in the case of the hippocampal gene set, they were not statistically significant for some genes. Together, these DARs indicate oligodendrocyte gene regulation may play a significant role in AD progression. Furthermore, while microglia are considered the primary targets of AD GWAS SNPs, our data highlight the possibility that a subset of loci may also exert their function through oligodendrocytes. Future experiments employing reporter assays or CRISPR Cas9 (Ran et al., 2013) genome editing in oligodendrocytes could explore this possibility.

### Age associated acetylation differences are enriched in the microglial population and correlated with gene expression differences

While microglial H3K27ac displayed strong colocalization with GWAS derived AD-associated SNPs, very few acetylation differences associated with Aβ load were detected. Instead, we found that compared to neuronal and OEG populations, the microglial population (combining all 26 samples from both dlPFC and hippocampus) displayed age-associated acetylation changes. We detected 444 age-associated DARs in microglia (FDR q < 0.05). In contrast, only 9 age-associated DARs were found in OEG, and none were found in neurons (Supplementary Figure 21). This analysis controlled for Aβ load, sex, and brain region differences. Of the 444 microglia DARs, 391 were hypoacetylated with increasing age, and 53 were hyperacetylated with increasing age (FDR *q* < 0.05) (Supplementary Table 10). We mapped these 444 DARs to nearby genes using GREAT (McLean et al., 2010) and identified 2 hypoacetylated peaks annotated to the amyloid precursor protein (*APP*) gene, and 6 hypoacetylated peaks near the *LRRTM3* gene, which is involved in positive regulation of Aβ formation (Supplementary Table 11). We also observed hyperacetylation at 3 distal peaks annotated to the FKBP4 gene, which is involved in tau protein binding and influences neurofibrillary tangle formation. Age-associated microglia DARs did not display significant colocalization with AD SNP heritability in S-LDSC analysis (Jansen GWAS coefficient = −2.17e-08, *p* = 0.71, observed scale heritability = −4.43e-05, s.e. = 9.86e-05, Kunkle GWAS coefficient = −3.86e-7, *p* = 0.98, observed scale heritability = −8.84e-4, s.e. = 0.0004).

Notably, these age-associated acetylation changes are correlated with age-associated transcriptional changes in human microglia profiled in a previous study (Olah et al., 2018). Genes annotated near age-associated hypoacetylated peaks (*n* = 307) in microglia displayed lower transcription in aged individuals (mean age = 94.07, s.d. = 0.95) compared to middle aged individuals (mean age = 53, s.d. = 5.29) (avg. log2fc transcription = −0.69, *p* = 1.2e-10). Similarly, genes annotated near age-associated hyperacetylated peaks (*n* = 50) displayed higher transcription in aged individuals compared to middle age subjects, but this was not statistically significant (avg. log2fc transcription = 0.25, *p* = 0.43). While the age range of our H3K27ac samples are limited (74.77–101.94), we note that microglia alone showed significant age-associated acetylation changes. Compared to other cell types, this may indicate microglia undergo gene regulatory adaptations sensitive to even advanced age. Future studies will have to probe a wider range of ages in order to fully dissect cell type-specific epigenetic responses to age.

## Discussion

We report the first H3K27ac profiles of sorted neurons, microglia, and OEG from both the hippocampus and dlPFC of postmortem human AD brain tissue. We find microglial H3K27ac peaks colocalize with common SNPs associated with LOAD risk, supporting previous findings (Gjoneska et al., 2015; Gosselin et al., 2017; Lake et al., 2017; Nott et al., 2019). While this suggests LOAD risk loci influence AD predisposition and progression through microglial processes, perhaps unexpectedly, comparison of H3K27ac peaks by Aβ load in microglia revealed few differences. Instead, we report H3K27ac is altered significantly with age in microglia, leading us to conclude that amongst the individuals analyzed, microglial H3K27ac is more responsive to advances in age than to Aβ load. Age-associated H3K27ac differences in microglia also correlated with age-associated transcriptional differences identified from a previous study (Olah et al., 2018). We note that heterogeneity within the microglial population in disease has been previously reported (Keren-Shaul et al., 2017; Mathys et al., 2017) and therefore, the possibility of Aβ-associated gene regulatory differences in microglia cannot be excluded based on our study. However, a recent manuscript utilizing both single cell and bulk RNA-seq techniques did not detect any transcriptional differences between microglia from AD and healthy aged individuals (Alsema et al., 2020). Combined, this suggests that the study of age-associated changes in microglia may provide a more promising avenue toward understanding the role of microglia in AD progression.

Interestingly, we find a subset of AD risk loci have significant H3K27ac signal in OEG relative to other cell types. These include risk loci associated with genes *CLU*, *BIN1*, and *PICALM*. Previous multi-scale network analyses have found oligodendrocyte transcript and protein modules are enriched for genes associated with AD risk loci, particularly *BIN1* and *PICALM* (McKenzie et al., 2017; Seyfried et al., 2017). Indeed, *BIN1* is highly expressed in oligodendrocytes, and is associated with white matter tracts in the human brain (De Rossi et al., 2016). Combined, these data suggest oligodendrocytes may play a significant role in the functionality of certain AD risk loci and their associated risk genes, and should be further investigated (Bartzokis, 2011).

We also find that OEGs harbor the largest H3K27ac differences associated with Aβ load, albeit in a region and sex-specific manner. dlPFC and hippocampus oligodendrocyte-enriched populations seem to mount distinct epigenomic signatures in response to AD but converge on similar biological processes. In the hippocampus (restricted to female subjects only, Aβ *n* = 5, no-Aβ *n* = 3), the promoters of genes associated with early and late-onset AD risk displayed hypoacetylation. This included EOAD risk genes *APP*, *PSEN1*, and *PSEN2*, and LOAD risk genes *BIN1*, *PICALM*, *ADAM10*, *ADAMTS4*, *FERMT2*, and *SORL1* (Lambert et al., 2013; Jansen et al., 2019; Kunkle et al., 2019). In addition, core oligodendrocyte processes such as myelination were also found to be significantly hypoacetylated. Sorted hippocampal oligodendrocyte nuclei from an independent cohort of ROSMAP individuals revealed a corresponding trend toward downregulation of the associated transcripts (de Leeuw et al., 2004; Lee et al., 2016). Importantly, previous AD studies demonstrate similar pathways are deregulated at the transcriptomic and proteomic levels in oligodendrocyte-enriched modules, as does a recent single-cell gene expression study (McKenzie et al., 2017; Seyfried et al., 2017; Mathys et al., 2019). Combined with our current findings, this suggests that the oligodendrocyte response to Aβ is an important feature of AD progression and merits further attention. However, the limited size of our dataset poses a caveat to data interpretation, especially because our analysis of the hippocampus is limited to female AD patients. The acetylation differences identified should be confirmed in a larger sex-matched cohort of individuals. In addition, positive selection of astrocytes will be necessary in future studies looking at cell type-specific epigenomic changes in AD since the NeuN-/Pu.1- population is likely a heterogenous mixture of cells.

We also observed an Aβ-associated dysregulation of promoters and enhancers of myelin-processing genes in dlPFC OEG (both sexes, Aβ *n* = 5, no-Aβ *n* = 5). However, these dlPFC DARs were hyperacetylated in AD individuals, including peaks annotated to *PSEN2*, *CLU*, *ADAMTS4*, *BIN1*, and *SORL1*. The DARs in the hippocampus are largely distinct from the DARs in the dlPFC. This disparity between brain regions may reflect oligodendrocyte heterogeneity in response to pathological insults, as well as region-specific differences in cell composition and pathologic severity. Alternatively, it may be associated with compensatory signaling in the prefrontal cortex that has been previously reported in neurodegenerative disorders (Grady et al., 2003). However, it cannot be ruled out that the differences we observe are due to an under-powered dataset. Despite this, it is apparent OEG H3K27ac represents a core feature of epigenetic dysregulation in both hippocampus and dlPFC. Whether these changes are primary drivers of AD pathology or secondary effects however is unclear. For example, numerous studies have shown that Aβ is toxic to oligodendrocytes (Xu et al., 2001; Lee et al., 2004; Nasrabady et al., 2018). As AD progresses, the gradual accumulation of Aβ could account for the deregulation of myelinating genes. Inflammatory microglia could also hinder myelination through the release of proinflammatory factors such as nitrogen species and cytokines, or the through the impaired phagocytosis of myelin debris (Peferoen et al., 2014). Alternatively, the altered acetylation of Aβ processing genes, AD risk genes and basic oligodendrocyte processes could play a role in the initiation of AD pathology. Future research could explore whether these gene regulatory changes are primary or secondary events in disease progression, or a mixture of both. In summary, our data demonstrates cell type-specific epigenomic deregulation occurs in AD, and we specifically highlight oligodendrocyte gene regulation as a target for future AD research.

## Materials and methods

### Source of brain tissue and pathologic data

Biospecimens and data came from autopsied participants in one of two prospective clinical-pathologic cohort studies, the Religious Orders Study or Rush Memory and Aging Project (ROSMAP). Both studies were approved by an Institutional Review Board of Rush University Medical Center. All participants signed an informed consent, an Anatomical Gift Act, and a repository consent to all their data and biospecimens to be repurposed. Details of the studies have been previously reported (Bennett et al., 2018). We collected 26 samples from 19 subjects enrolled in ROSMAP. 7 subjects were sequenced for both hippocampus and dlPFC, 3 subjects were unique to the dlPFC dataset and 9 subjects were unique to the hippocampus dataset. We provide (Supplementary Figures 2, 3, 18) with jitter plots summarizing overall amyloid load (averaged across 8 brain regions), tangles, global cognition score (cogn_global_lv), consensus cognitive diagnosis (cogdx), Braak stage, CERAD score, age at death, years of education and postmortem interval (pmi) for these samples. For more details about these variables, please refer to Supplementary Table 1 or the Rush Alzheimer’s Disease Center (RADC) variables list: https://www.radc.rush.edu/docs/var/variables.htm.

### Fluorescence-activated nuclei sorting

Fresh frozen dlPFC and hippocampus samples were retrieved from -80°C storage and thawed on ice, then disrupted with a handheld homogenizer. Samples were fixed with 1% paraformaldehyde for 10 min at room temperature. Fixation was quenched with glycine for 5 min. Nuclei were isolated by dounce-homogenization followed by filtration through a 70 μM cell strainer (cat no. 21008-952, VWR, Radnor PA). To immunotag cell type specific nuclei, anti-NeuN antibody conjugated to Alexa Fluor 488 (cat no. MAB377X, EMD Millipore, Burlington MA), and anti-PU.1 antibody conjugated to Alexa 647 (cat no. 2240S, Cell Signaling Technology, Danvers MA) were incubated with nuclei at 4°C for 1 h and overnight, respectively. Samples were strained through a 40 μm filter (21008-949, VWR) and stained with the nuclear marker DAPI (D9542, Sigma Aldrich, St. Louis MO) before flow cytometry. First, single nuclei were gated from debris and doublets using DAPI staining. Second, NeuN+ nuclei were gated from NeuN- nuclei. Lastly, NeuN- nuclei were gated as either PU.1+ or PU.1- negative based on average PU.1-647 fluorescence distribution. Fluorescence activated nuclei sorting was performed until 400,000 nuclei were collected for each cell type (NeuN+, Pu.1+, and NeuN-/Pu.1-) using the FACSAria (BD Biosciences, US).

### Chromatin immunoprecipitation

Following sorting, chromatin was fragmented into 200–600 bp fragments using the Diagenode bioruptor. Fragmented samples were split equally into two tubes such that each tube contained an equivalent of chromatin from 200,000 nuclei. All ChIPs were carried out in duplicate. Samples were pre-cleared with BSA-blocked Protein A sepharose beads (cat no. GE17-0780-01, Sigma Aldrich) for 4 h at 4°C. At this point, 1% input was collected and stored at -20°C. Chromatin was incubated with 2 μg of Histone H3 (acetyl K27) antibody (cat no. ab4729, abcam, Cambridge UK) overnight at 4°C. Chromatin fragments bound to the antibody were pulled down with BSA-blocked Protein A sepharose beads for 4 h at 4°C. To reduce non-specific binding, the bead-chromatin complex was washed four times with ice-cold RIPA buffer. Immunotagged chromatin was eluted from beads through shaking at 65°C for 15 min. Both 1% input and ChIP were de-crosslinked overnight in T_50_E_10_S_1_ buffer at 65°C. Reverse crosslinked chromatin was treated with RNase A and Proteinase K. DNA was purified using phenol-chloroform extraction. Following ethanol precipitation, samples were resuspended in 10 mM Tris-HCl buffer and stored at −20°C.

### Chromatin immunoprecipitation followed by sequencing high-throughput sequencing

A portion of the sample was used to assess enrichment for cell-type specific H3K27ac peaks *via* qPCR. If the sample passed qPCR quality control, libraries were generated from the remaining sample. Library generation was performed using the KAPA Hyper Prep Kit (KK8504, Kapa Biosystems). After amplification and quantification, a portion of the library was used for a second qPCR to ensure enrichment of cell-type specific H3K27ac peaks. If the sample passed the second qPCR quality control, it was submitted to the MIT BioMicro Center for fragment analysis, followed by sequencing. The 40-bp single-end sequencing was performed using the Illumina HiSeq2000 platform according to standard operating procedures.

### Quality control, consensus peak set generation, and read counting

For peak calling, the AQUAS ChIP-Seq workflow^1^ was used. To perform quality control, the two technical replicates for each sample were individually input to the AQUAS workflow to compute standard ENCODE quality metrics (Landt et al., 2012) such as NSC, RSC, NRF, PBC1, PBC2, FRiP, replicate consistency etc. All samples that did not meet quality standards of (NSC > 1.01, RSC > 0.4, PBC1 > 0.5) were discarded at this point. A full table of quality metrics and retained/filtered samples is provided in Supplementary Table 2. The workflow uses Burrows-Wheeler alignment (Li and Durbin, 2009), Samtools (Li et al., 2009) for processing alignments, MACS2 (Zhang et al., 2008) for peak calling, and PICARD^2^ for removing PCR duplicates. Peak reproducibility is assessed by overlapping peaks across groups of sample replicates and pseudoreplicates using a method similar to irreproducible discovery rate (IDR) (Li et al., 2011) analysis. All analysis was performed on the hg19 reference genome.

To call a consensus peak set, we aimed to filter out noisy peaks associated with individual samples while still retaining peaks associated with individual brain regions, cell types and Aβ pathology. To achieve this, we called 12 sets of reproducible peaks on different subsets of samples defined by these variables. More explicitly, reproducible peaks were called for each of the following subsets of samples using the AQUAS workflow:

a. Hippocampus, no Aβ, neuron
b. Hippocampus, no Aβ, microglia
c. Hippocampus, no Aβ, OEG
d. Hippocampus, w/Aβ, neuron
e. Hippocampus, w/Aβ, microglia
f. Hippocampus, w/Aβ, OEG
g. dlPFC, no Aβ, neuron
h. dlPFC, no Aβ, microglia
i. dlPFC, no Aβ, OEG
j. dlPFC, w/Aβ, neuron
k. dlPFC, w/Aβ, microglia
l. dlPFC, w/Aβ, OEG

Then, these 12 sets of peaks were merged using the bedtools merge (Quinlan and Hall, 2010) utility to construct a single consensus peak set. To account for local depletions in chromatin intensity profiles (“dips”) (Ernst et al., 2011), peaks that were less than 200 bp apart were merged during this step. We propose this merged consensus peak set comprising 352,012 peaks as a reference set of peaks active in the three profiled brain cell types of the dlPFC and hippocampus of AD and non-AD subjects and use it in downstream analyses. To confirm brain enrichment, we downloaded all 98 H3K27ac profiles in Roadmap Epigenomics and computed intersections with our consensus peak set using bedtools jaccard (Quinlan and Hall, 2010). We then ranked the 98 intersections based on the resulting jaccard index to see if brain tissues/cell types ranked highest. The featureCounts (Liao et al., 2014) package was used to count the read signal at these peaks for every ChIP-Seq experiment that passed quality control. This read count matrix was then used in downstream analysis for validation of sorting, for PCA analysis, and for identifying differentially acetylated regions using DESeq2 (Love et al., 2014). We also used this count matrix for variance partition analysis using the variancePartition R package (Hoffman and Schadt, 2016; Hoffman and Roussos, 2021) using a mixed effects model design that included brain region, cell type, age, amyloid pathology indication as fixed effects and subject ID as a random effect.

### Cell type-specific hyperacetylated peak sets

For each of the three cell type populations, we used the negative binomial model of DESeq2 (Love et al., 2014) inputting the full consensus peak set and contrasting the focal cell type against the other two cell types to identify 3 subsets of cell type-specific differentially hyperacetylated peaks. Peaks were defined as differentially hyperacetylated if they displayed a positive log2 fold change and passed FDR control (q < 0.05) across each of the 352,012 peaks that passed independent filtering criteria in DESeq2. This set of peaks was used in heritability enrichment analyses using permutation testing (Consortium et al., 2015; Gjoneska et al., 2015) and stratified LD-score regression (Bulik-Sullivan et al., 2015; Finucane et al., 2015, 2018). For these analyses, a background peak set was constructed by creating a union of these peaks. Further, the log2-fold change values from DESeq2 were used to assess cell type-specificity of individual peaks at AD GWAS loci. A full set of fold changes representing cell type-specificity is also reported in Supplementary Table 3.

### Sorting validation and identification of cell types by comparison to single nucleus ribonucleic acid-sequencing clusters

The consensus set of merged peaks were annotated to their nearest genes using the annotatePeaks tool in HOMER (Heinz et al., 2010). Marker gene sets for 15 cell type clusters were downloaded from the Habib et al. (2017) study which profiled frozen human hippocampus and PFC samples from five recently deceased, non-diseased male donors aged 40–65 (three samples from PFC and four samples from hippocampus). According to the study, the average post-mortem ischemic interval for tissues was 12.4 h. For each single nucleus RNA-seq cluster, H3K27ac peaks annotated to the marker gene set were obtained. The cell type-specificity log2fc values (see **Cell type-specific hyperacetylated peak sets**) for these 15 marker gene annotated peak sets were then extracted and a one-sided *t*-test was used to test whether the mean of these log2fc values was significantly greater than 0.5 (∼1.4 fold change). A significant result from this test indicated the enrichment of a cell type identified in Habib et al. (2017) in our ChIP-Seq profiled population. The test was conducted for every pair of focal ChIP-Seq population and single nucleus RNA-Seq cluster. *P*-values were adjusted for multiple hypothesis testing using Benjamini Hochberg FDR correction across all 45 tests (15 snRNA-seq cell types * 3 ChIP-seq populations). Adjusted *p*-values are reported in the results section.

To test whether TSS distal peaks confound these results, the above analyses were also conducted on peaks that are near promoters of the marker genes by only selecting the peaks that are less than 5 kilobases away from transcription start sites (TSS) of the 15 gene sets. Multiple hypothesis correction was done similarly using Benjamini Hochberg FDR correction across all 45 tests.

In addition, we verified sorting efficacy in each of the 26 tissue samples individually. First, we input the full count matrix to DESeq2, collapsed technical replicates, and then computed variance stabilized (vst) counts. Then, we extracted the vst counts at each peak annotated to marker genes for the 15 cell type clusters defined in Habib et al. (2017) At each peak, we computed cell type-specificity log2fc values for individual tissue samples by dividing the vst count for the focal cell type by the mean vst count of the two non-focal cell types. Then, we computed the mean of these cell type-specificity log2fc values for all 15 peak sets. These values were z-score normalized and plotted in the form of a heatmap. Further, to check whether sorting efficacy is different between Aβ and no Aβ samples, male and female samples, or dlPFC and hippocampus samples, we averaged these log2fc values across samples in these individual groups, z-score normalized them, and plotted them in a separate heatmap. To test whether distal peaks confound these results, we also repeated this entire analysis by restricting to only TSS proximal putative promoter peaks (<5 kb from TSS).

### Sorting validation using single nucleus ATAC-seq

We download narrowPeak files for all 24 snATAC-seq cell type clusters profiled in Corces et al. (2020). Then we intersected all 24 peak sets to our 3 cell type-specific hyperacetylated peak sets using the bedtools jaccard (Quinlan and Hall, 2010) utility. For each of the 3 cell types, we then sorted the resulting intersections based on the computed jaccard index to identify cell types enriched in our H3K27ac dataset.

### Stratified LD-score regression analysis

GWAS summary statistics from two studies, Kunkle et al. (2019) and Jansen et al. (2019) were downloaded and stratified LD-score regression (S-LDSC) (Bulik-Sullivan et al., 2015; Finucane et al., 2015, 2018) was used to compute AD SNP heritability enrichment in cell type-specific differential peaks against the merged background set. The standard workflow described by the authors was used and LD scores were computed based on custom annotations derived from hyperacetylated peaks called on each cell type and compared against custom annotations derived from the merged background set constructed from the three cell type-specific peak sets. A baseline model representing annotations from 53 different tissues was also included to compute the enrichment coefficients as recommended by the LDSC authors. The regression coefficients for each population were extracted and plotted. A significant result from this test indicates an enrichment of genetic risk for LOAD in regions that are actively regulating gene expression in the cell type, suggesting a role for that cell type in influencing predisposition toward LOAD. Benjamini Hochberg FDR correction was applied across the three tests. To estimate heritability, we conducted a separate analysis using a different baseline model based on 82 annotations as recommended by the S-LDSC authors. We report observed scale heritability estimates from this analysis. Similar analyses were conducted with Schizophrenia GWAS study. S-LDSC analysis was also conducted for cell type reproducible peaks (see **Cell type reproducible peak sets**) in similar fashion.

### Enrichment test for colocalization of Alzheimer’s disease-associated variants with cell type-specific peaks

To test whether choice of computational method may alter conclusions of the S-LDSC analysis, we used another approach that utilizes a permutation test. LD-pruning was applied (LD > 0.5) on both GWAS datasets based on the 1,000 genomes reference (Gibbs et al., 2015). SNPs overlapping protein coding sequence (Zerbino et al., 2018) were filtered out along with SNPs in tight linkage disequilibrium (LD > 0.5). SNPs with *p*-values less than 1e-3 were selected and overlap annotations were created for each set of differential cell type-specific peaks (see **Cell type-specific hyperacetylated peak sets**). A permutation test was used to compute heritability enrichment of AD-associated SNPs in a focal foreground set of differential peaks for a cell type against the merged background set. SNPs were permuted 1,000,000 times preserving distance to gene, minor allele frequency and the number of variants that are in LD. Benjamini Hochberg FDR correction was applied across the three tests.

### Cell type reproducible peak sets

To robustly identify all peaks active in a cell type (not just differentially hyperacetylated cell type-specific peaks), we also generated reproducible peak sets for each cell type. For each cell type, pooled alignments of all dlPFC and all hippocampus samples from subjects without Aβ load were input to the AQUAS workflow. Only subjects without Aβ load were used so as not to include peaks that may be associated with AD. This peak set was used to generate the browser visualization tracks at the loci containing the *INPP5D*, *BIN1* and *PICALM* genes (Figures 2D–F). Browser tracks for *INPP5D*, *BIN1*, and *PICALM* were generated using the integrative genomics viewer (IGV) (Robinson et al., 2011) and pygenometracks (Ramírez et al., 2018), and edited later. Using these three cell type reproducible peak sets, we also did S-LDSC analysis. For this S-LDSC analysis, a new background peak set was used, which was created by merging the 3 cell type reproducible peak sets. Peaks less than 200 bp apart were merged to account for H3K27ac dips. Benjamini Hochberg FDR correction was applied across the three tests.

### Differentially acetylated regions associated with Aβ load

Differentially acetylated regions were identified using the negative binomial model of DESeq2. For each differential acetylation model setting (see Supplementary Table 7 for details), a subsetted count matrix was generated that includes only the subset of samples corresponding to the brain region, sex, or cell type included. This matrix was input to DESeq2 and DARs were identified by contrasting high Aβ samples against no Aβ samples. For each contrasts, DARs were called at a FDR *q*-value cutoff of 0.05, correcting for multiple hypothesis across each of the 352,012 peaks that passed independent filtering criteria in DESeq2. No covariates were included in the initial linear models. However, *post hoc* analysis was conducted for the two OEG DAR sets described in detail in the results, by fitting individual models to the OEG female hippocampus samples and the OEG dlPFC samples for age at death, years of education, pmi, and RSC. Additionally, we covaried Aβ and RSC (design **Aβ + RSC**) and tested whether effect sizes for Aβ remained correlated with the original models used to identify the DARs. Log2fc values from this analysis are provided in Supplementary Table 8. We tested whether the identified DARs were robust to a larger multiple hypothesis correction conducted across all 13,394,888 tests from each contrast. 6 of the 1,962 hippocampus female OEG hypoacetylated peaks and 5 of the 1,029 dlPFC OEG hyperacetylated peaks were robust to this correction (q < 0.05). Further, for the two OEG DAR sets, we conducted replication analysis in the other brain region to test whether the changes were directionally consistent (Supplementary Figure 22). For the OEG hippocampus female analysis, we compared the log2fc values for all peaks with those from the analysis of OEG dlPFC female samples. We found that the log2fc values displayed low Pearson correlation (r = 0.06), although the correlation was significant (*p* = 7.55e-25). For the 1,962 OEG female hippocampus hypoacetylated peaks, directionality of acetylation change was not consistent between the two brain regions. 1,804 of 1,962 peaks displayed increased acetylation in dlPFC female OEG Aβ samples (log2fc > 0) and the remaining 158 were directionally consistent (log2fc < 0). For the analysis in OEG dlPFC samples, we compared the log2fc values with the analysis of OEG hippocampus samples of both sexes. We observed similar results, the log2fc values displayed low Pearson correlation (*r* = 0.06), although the correlation was significant (8.08e-14). Direction of acetylation change was again not consistent across the two brain regions. 815 of 1029 dlPFC OEG hyperacetylated peaks displayed decreased acetylation in OEG hippocampus Aβ samples (log2fc < 0) samples and the remaining 214 were directionally consistent (log2fc > 0).

We also provide plots of normalized read counts at DARs against these variables to look for increasing or decreasing trends. For this, variance stabilized (vst) read counts (Love et al., 2014) were computed and z-score normalized for each peak, and box plots were plotted against age, years of education, pmi, and RSC to look for relationships (Supplementary Figures 12, 13). Vst transformed read counts across all peaks were used for heatmap visualizations. DAR sets were annotated to their nearest genes using the annotatePeaks tool in HOMER (Heinz et al., 2010) and the distribution of distance to TSS output from HOMER was plotted for the two OEG Aβ associated DAR sets as well as the remaining DARs. A hypergeometric test was used to test for promoter enrichment in the OEG DAR sets by treating peaks < 5 kb TSS as successes and peaks > 5 kb from TSS as failures. The background for the hypergeometric test was the full set of 352,012 peaks.

For OEG hippocampus samples of both sexes, we conducted an additional DESeq2 analysis with the following design: **sex + binary Aβ load status (high or none) + sex:binary Aβ load status interaction**. Peaks significant for the interaction term of **sex:binary Aβ load status** were then extracted to assess sex-specificity of OEG DARs detected in female hippocampus samples. An FDR *q* < 0.05 was used as the cutoff to correct for multiple hypothesis across all 352,012 tests that passed independent filtering criteria in DESeq2. Only 3 peaks were called differential (FDR q < 0.05) for the interaction term. Furthermore, only 1 of the 3 peaks were members of the set of 1962 DARs detected in OEG female hippocampus samples, suggesting that the differential acetylation at almost all (1961/1962) of these peaks is unlikely to be sex-specific.

Genome browser visualizations were created for the two OEG DAR sets at EOAD and LOAD risk loci, as well as highly differentially acetylated loci using pygenometracks (Ramírez et al., 2018). Custom UNIX commands and the UCSC bigWigMerge (Kent et al., 2010) tool were used to create average H3K27ac signal tracks in OEG samples from subjects with and without Aβ load. Tracks for DESeq2 log2fc and UCSC gene annotations (Karolchik et al., 2004) were included. A UCSC genome browser track hub containing bigwig signal tracks and peak annotations is made available at: https://genome.ucsc.edu/cgi-bin/hgHubConnect.

S-LDSC was used to test for AD SNP heritability enrichment from both AD GWAS studies in the two OEG specific DAR sets. The full consensus peak set of 352,012 peaks was used as background and the 53-annotation baseline was used to estimate enrichment coefficients and *p*-values. Since these were independent and individual tests, nominal *p*-values from the analysis were reported. Heritability estimates were computed in a similar way, but with the 82-annotation baseline according to author recommendations (see **Stratified LD-score regression analysis**).

### Gene ontology enrichment analysis for oligodendrocyte-enriched glial differentially acetylated regions sets

The GREAT (McLean et al., 2010) web tool was used for computing enrichments for ontological annotations associated with genes proximal to DAR sets. GREAT analysis was performed separately on the two biggest OEG DAR sets as well as the remaining DARs not in those sets. In addition, we used GREAT to compute pathway enrichments for neuron, microglia and OEG cell type-specific peaks. The consensus brain peak set of 352,012 peaks (see **Quality Control, consensus peak set generation, and Read Counting**) was used as the background for each of the aforementioned GREAT analyses. A heatmap of the fold enrichment returned by GREAT was plotted for any GO Biological Process that passed a q-value cutoff of 0.05 and was associated with a minimum of 5 genes in any of the GREAT analyses. In addition, fold enrichment for the KEGG Alzheimer’s Disease Pathway was plotted in the heatmap. To confirm that we were not enriching for oligodendrocyte signal in GREAT analysis for the OEG DARs, we re-ran GREAT using a custom background created from cell type-specific hyperacetylated peaks for OEG (see **Cell type-specific hyperacetylated peaks**) and extracted only the enrichment *p*-values for processes of interest such as central nervous system myelination, oligodendrocyte differentiation etc. We report nominal *p*-values since we only test two or three individual processes. Since GREAT requires every foreground peak to be part of the background set, foreground peaks were added into the background set for this analysis. Furthermore, an additional GREAT analysis was run on an OEG cell type-specific background peak set that was specific to the brain region in which the foreground DAR set was detected. More specifically, for the hippocampus hypoacetylated OEG DARs, this analysis included a background that was derived from cell type-specific hyperacetylated peaks only in the hippocampus. To generate this background, DESeq2 was run on the full consensus peak set but inputting only the subsetted matrix containing the hippocampus samples, contrasting OEG against the other two cell types to identify hippocampus OEG-specific peaks (log2fc > 0, FDR q < 0.05). For the hippocampus hypoacetylated DAR set, enrichment for central nervous system myelination (hippocampus background *p* = 2.3e-4, region fold enrichment = 2.55; both background *p* = 5.89e-5, region fold enrichment = 2.84), oligodendrocyte development (hippocampus background *p* = 6.13e-3, region fold enrichment = 1.72; both background *p* = 1.83e-3, region fold enrichment = 1.89), and oligodendrocyte differentiation (hippocampus background *p* = 2.5e-2, region fold enrichment = 1.4; both background *p* = 5.99e-3, region fold enrichment = 1.54) were still detectable. For the OEG dlPFC hyperacetylated DAR set, a similar OEG cell type-specific background was created, this time by inputting only the subsetted matrix containing the dlPFC samples to DESeq2. For the OEG dlPFC DAR set, enrichments for central nervous system myelination (dlPFC background *p* = 4.46e-5, region fold enrichment = 3.86; both background *p* = 6.86e-5, region fold enrichment = 3.7), and oligodendrocyte differentiation (dlPFC background *p* = 2.75e-3, region fold enrichment = 1.89; both background *p* = 4.6e-3, region fold enrichment = 1.81) was still detectable.

### Protein-protein interaction network analysis for oligodendrocyte-enriched glial differentially acetylated regions sets

We used the STRING (Szklarczyk et al., 2021) plugin in the Cytoscape (Shannon et al., 2003; Cline et al., 2007; Saito et al., 2012) application to construct PPI networks for genes annotated to the two OEG DAR sets. Since the output network contained a lot of edges, it was largely not interpretable. Therefore, we clustered the nodes in the network using the in-built Markov chain (MCL) clustering algorithm (Enright et al., 2002). We found that setting the MCL granularity parameter to 2.5 for both gene sets led to well separated and interpretable clusters. Most genes/proteins clustered into small groups (1–9 genes per cluster) and there were only a few clusters with > 10 genes which could be interpreted in further analysis. Therefore, we only visualized and focused downstream analyses on these top clusters based on the number of genes present in them. For visualization, we set the size of the nodes and their labels using a continuous mapping on the “betweenness centrality” of the nodes which we obtained by running Tools - > Analyze Network within Cytoscape. We then conducted Gene Ontology enrichment analysis using GREAT for genes in each of these top clusters using DAVID (Sherman et al., 2022) and annotated the network visualization with selected GO BP terms passing FDR q < 0.05. Our full cytoscape session file is available in our data upload and can be directly imported into Cytoscape: http://daphne.compbio.cs.cmu.edu/files/eramamur/ad_h3k27ac_3ct_data_resource/oeg_dars_cytoscape_session.cys.

### Motif enrichment analysis on oligodendrocyte-enriched glial differentially acetylated regions sets

We used the findMotifsGenome package within HOMER (Heinz et al., 2010) to identify motifs enriched in the OEG DAR sets. We conducted this analysis in a differential motif discovery setting using a background set of peaks derived from the full set of 352,012 peaks. Since the 1,962 DARs identified in female hippocampus OEG samples displayed high promoter enrichment, HOMER would enrich only promoter motifs when using the full peak set as the background. To overcome this bias associated with promoters, we ran the analysis with a background peak set selected from the full set of peaks that matches the distribution of distance to TSS with the foreground DAR set. For each peak in the foreground DAR set, we randomly selected a single peak that had a distance to TSS which was within a 100 bp of the distance to TSS value of the foreground peak. If the peak was already present in the sampled background set, we repeated the process until we found a peak which wasn’t already present in the sample. We constructed 10 such background peak sets and ran 10 motif enrichment analyses with these different backgrounds. We visually compared the 10 different motif enrichment analyses and identified motifs enriched reproducibly across these analyses. For motif enrichment analysis on the 1,029 OEG dlPFC DARs, we used the full set of 352,012 peaks as the background set since there was no specific enrichment for promoters in this DAR set. Results of our motif enrichment analyses are provided in our data upload: http://daphne.compbio.cs.cmu.edu/files/eramamur/ad_h3k27ac_3ct_data_resource/motif_enrichment.

### Ribonucleic acid extraction, reverse transcription and quantitative polymerase chain reaction in postmortem hippocampus

An independent set of hippocampal samples from the ROSMAP cohort were used for RT-qPCR validation. Samples were prepared for FANS as described previously. To isolate oligodendrocyte, microglia, astrocyte, and neuronal nuclei, samples were stained overnight at 4°C with anti-Olig2 antibody conjugated to Alexa Fluor 488 (cat no. MABN50A4, EMD Millipore, Burlington MA), anti-PU.1 antibody conjugated to Alexa Fluor 647 (cat no. 2240S, Cell Signaling Technology, Danvers MA), anti-GFAP conjugated to Alexa Fluor 555 (cat no. 3656, Cell Signaling Technology, Danvers MA), and stained for 1 h with anti-NeuN conjugated to biotin (cat no. MAB377B, EMD Millipore, Burlington MA), and for 1 h with Brilliant Violet 711 Streptavadin (cat no. 405241, BioLegend, San Diego, CA). Fluorescence activated nuclei was performed until at least 100,000 Olig2-positive nuclei, NeuN-positive nuclei, GFAP-positive nuclei, and PU.1-positive nuclei were collected for each sample.

Following sorting, nuclei were treated for 15 min with Proteinase K at 50°C and then for 13 min at 80°C. RNA was extracted using Direct-zol RNA MicroPrep kit (Zymo Research) according to manufacturer’s instructions. Reverse transcription of RNA was carried out using Invitrogen SuperScript IV First Strand Synthesis System (Oligo dT) according to manufacturer’s protocol. qPCR was performed using a Bio-Rad CFX-96 quantitative thermocycler and SsoFast EvaGreen Supermix (Bio-Rad). Relative changes in gene expression were determined using the 2^–Δ^ ^Δ^ ^Ct^ method. The geometric mean of cycle numbers from RPL13, CYC1, and GADPH were used for housekeeping Ct values. Fold change in gene expression for high-Aβ samples was computed relative to the combined average gene expression of low and mid-Aβ load samples. Mid-Aβ was defined as Aβ load scores between 1 and 7.71. High-Aβ was defined as Aβ load scores higher than 7.71. These cutoffs were chosen based on the observation that the female hippocampal OEG ChIP-seq samples with Aβ load equal to or higher than 7.71 displayed normalized read counts below the mean levels when Aβ was treated as a continuous variable (Supplementary Figure 12B). Effect sizes for gene expression changes were calculated using Hedge’s g. Primer sequences used for RT-qPCR can be found in Supplementary Table 12.

### Comparison with single nucleus gene expression from postmortem dorsolateral prefrontal cortex

Hyperacetylated DARs in dlPFC OEG were assessed for nearby transcriptional differences in the snRNA-seq study from Mathys et al. (2019). The nearest genes of the hyperacetylated DARs were obtained using annotatePeaks in HOMER. Only genes where the closest peak was < 5 kb from the TSS were retained to reduce false positives. This resulted in a filtered list of 500 genes. Oligodendrocyte-specific log2FC values were obtained from the snRNA-seq study for two different contrasts (i). no pathology vs. early pathology and (ii). no pathology vs. pathology). Then, a one-sample one-sided *t*-test was used to test whether there is an average increase in transcription at these genes in AD (null hypothesis avg. log2fc = 0, alternative hypothesis avg. log2fc > 0). Violin plots of these log2FC values were plotted with *p*-values indicating the results of the aforementioned *t*-test (*p* < 0.05). Benjamini Hochberg FDR corrected *q*-values were reported for these tests. Transcription log2fc of specific AD risk genes and genes near highly hyperacetylated peaks were also plotted as bar plots (for both contrasts). In these bar plots, the differential expression FDR q-values from the snRNA-seq study were also reported. We also computed general enrichment of genes annotated to the dlPFC DARs in differentially expressed genes (FDR q < 0.05) using separate hypergeometric tests for both oligodendrocyte specific contrasts. Since these are single tests, raw *p*-values are reported.

### Differentially acetylated regions associated with age

Age-associated changes in H3K27ac levels were identified using DESeq2 on the full consensus peak set (see **Quality Control, consensus peak set generation, and read counting**). For each cell type, a subsetted count matrix was created that included all 26 samples (both cases and controls) for that cell type from both brain regions. H3K27ac level was modeled as a linear function of **sex + binary Aβ load status (high or none) + brain region (hippocampus or dlPFC) + age at death** in DESeq2, to control for the effects of sex, AD pathology and brain region. Log2FC and FDR q values were extracted for the age term. In addition, another analysis (“total”) that included all samples from every cell type was run to test for cell type agnostic effects of age on H3K27 acetylation. Histograms of the log2FC values were plotted for each cell type to determine which cell types displayed the strongest age-associated differential H3K27 acetylation. Differential age-associated peaks were identified for each cell type at FDR q < 0.05 correcting for multiple tests across all of the 352,012 peaks passing independent filtering criteria in DESeq2. Hypoacetylated and hyperacetylated peaks were then defined based on the sign of log2fc (log2fc > 0 for age-associated hyperacetylated peaks; log2fc < 0 for age-associated hypoacetylated peaks). Microglia age-associated peaks were then put into GREAT with default parameters to associate nearby genes and assess biological functions. For heatmap visualization, variance stabilizing transformation (vst) was applied on the full matrix and only the differential peaks were extracted.

### Comparison with microglia RNA-seq study

To test whether age-associated H3K27ac differences are associated with differences in transcription, we compared our dataset to an RNA-seq dataset of human dlPFC microglia published in Olah et al. (2018). Microglia differential gene expression log2fc values and *p*-values between middle aged (mean age = 53, s.d. = 5.29) and aged (mean age = 94.07, s.d. = 0.95) individuals were downloaded from Supplementary Data. Age-associated hypoacetylated and age-associated hyperacetylated peaks in microglia were then separately annotated to nearby genes using the annotatePeaks tool in HOMER. For the 391 hypoacetylated peaks, this was able to retrieve 307 associated genes, and for the 53 hyperacetylated peaks, this was able to retrieve 50 associated genes. The distribution of transcription log2fc values was plotted for these genes. To look for agreement between the direction of H3K27 acetylation differences and differences in transcription of associated genes, we used one sided *t*-tests. More specifically, for genes annotated to age-associated hyperacetylated peaks, we tested whether they displayed an increase in expression (null hypothesis: avg. log2fc = 0; alternate hypothesis: avg. log2fc > 0). For genes annotated to age-associated hypoacetylated peaks, we tested for the opposite effect (null hypothesis: avg. log2fc = 0; alternate hypothesis: avg. log2fc < 0). We also tested whether mean log2fc values differed significantly between genes associated with hyperacetylated peaks and genes associated with hypoacetylated peaks using a *t*-test (null hypothesis: avg. log2fc age-associated hypoacetylated = avg. log2fc age-associated hyperacetylated; alternate hypothesis: avg. log2fc age-associated hyperacetylated ≠ avg. log2fc age-associated hyperacetylated). Since these are individual tests, nominal *p*-values were reported.

## Data availability statement

Raw ChIP-sequencing data, processed data files, and accompanying metadata presented in the study are deposited in the AD Knowledge Portal (https://adknowledgeportal.org) under accession number syn38294603. The AD Knowledge Portal is a platform for accessing data, analyses, and tools generated by the Accelerating Medicines Partnership (AMP-AD) Target Discovery Program and other National Institute on Aging (NIA)-supported programs to enable open-science practices and accelerate translational learning. The data, analyses, and tools are shared early in the research cycle without a publication embargo on secondary use. Data are available for general research use according to the following requirements for data access and data attribution (https://adknowledgeportal.synapse.org/DataAccess/Instructions). For access to the data in this manuscript, see https://doi.org/10.7303/syn38294603.1. A data use agreement will be needed to access the data. Additional processed data files are available at: http://daphne.compbio.cs.cmu.edu/files/eramamur/ad_h3k27ac_3ct_data_resource/. Analysis code is available at: https://github.com/pfenninglab/ad_h3k27ac_3ct_release.

## Ethics statement

The studies involving human participants were reviewed and approved by the Rush University Medical Center. The patients/participants provided their written informed consent to participate in this study.

## Author contributions

JC, GW, ER, DB, AP, and L-HT contributed to the study design. AP and L-HT coordinated and directed the study. JC performed FANS and ChIP-seq for dorsolateral prefrontal cortex samples. GW performed FANS, ChIP-seq, and qRT-PCR for hippocampus samples. ER performed ChIP-seq processing and led the computational analyses. YY did the aging analysis supervised by AP and ER. LG performed the initial genetics integration analysis supervised by AP. ER, GW, JC, YY, DB, L-HT, and AP wrote and edited the manuscript. ER, GW, L-HT, and AP contributed to revisions. All authors contributed to the article and approved the submitted version.

## Footnotes

1
https://github.com/kundajelab/chipseq_pipeline

2
http://broadinstitute.github.io/picard/
