# Supplementary material for: Cell type-specific histone acetylation profiling of Alzheimer’s disease subjects and integration with genetics
Source: Front Mol Neurosci. 2023 Jan 6;15:948456. doi: 10.3389/fnmol.2022.948456 (PMC9853565; doi:10.3389/fnmol.2022.948456)
Supplement: Supplementary file 14 [file Presentation_1.zip › figure_captions.docx]

**Figure 1:** FANS sorting captures neurons, microglia and oligodendrocyte enriched populations from postmortem brain tissue. (A) Workflow for sorting nuclei and performing H3K27ac ChIP-seq from postmortem human brain tissue: nuclei were isolated from fresh frozen hippocampus or prefrontal cortex and FANS was performed to collect NeuN+, Pu.1+, and NeuN-/Pu.1- populations. H3K27ac ChIP-seq was performed on each population (B). Genome browser visualization of H3K27ac signal over background (Input) averaged across all profiled samples for the three populations. Loci containing the genes RBFOX3 (NeuN), SPI1 (Pu.1) and OLIG2 (an oligodendrocyte marker) are visualized (C). Top. heatmap displaying average H3K27ac enrichment at the promoters of marker genes (<5kb from TSS) from 15 cell type clusters profiled in (Habib et al., 2017). Rows represent individual tissue samples. Columns represent the 15 different cell type clusters and are repeated three times to display NeuN+ specificity, Pu.1+ specificity and NeuN-/Pu.1- specificity. bottom. collapsed version of the top heatmap created by averaging the log2fc values for groups of samples defined by Aβ load, sex and brain region. (Habib et al., 2017) cell type cluster abbreviations are defined here: exPFC, glutamatergic neurons from the PFC; GABA, GABAergic interneurons; exCA1/3, pyramidal neurons from the hippocampus CA region; exDG, granule neurons from the hippocampus dentate gyrus region; ASC, astrocytes; MICROGLIA, microglia; OLIGO, oligodendrocytes; OPC, oligodendrocyte precursor cells; NSC, neuronal stem cells; END, endothelial cells.

**Figure 2:** AD associated SNPs derived from GWAS prefer to colocalize with peaks enriched in the microglial population relative to peaks enriched in the OEG and neuronal populations (A,B). Results of stratified LD score regression from two AD GWAS studies(Jansen et al., 2019; Kunkle et al., 2019) and cell type-specific H3K27ac peaks. Plots show the estimated LD score regression coefficient for the three peak sets. Benjamini Hochberg FDR corrected q-values across the three tests for enrichment are indicated above each bar (C). Cell type enrichment of peaks annotated to sentinel SNPs at AD risk loci identified by (Jansen et al., 2019) and (Kunkle et al., 2019). Plots show fold change (log2-transformed) of H3K27ac signal for each population against the other two populations for (i) in black: peaks closest to the sentinel SNP at each locus associated with AD from GWAS, and (ii) in red: promoter peaks of early onset AD risk genes (*APP*, *PSEN1*, *PSEN2*). ∗Indicates DeSeq2 FDR q < 0.05 for the cell type-specific contrast. Sentinel SNPs that introduce missense mutations in proteins or SNPs where the closest H3K27ac peak is annotated > 1kb away are not included. This restriction was to ensure the analysis comprised only of SNPs that likely have functional effects on promoters or enhancer activity (D–F) top: Genome browser tracks of (i) reproducible peaks in each cell type for subjects without Aβ load, (ii) average H3K27ac signal in subjects without Aβ load for each cell type, and (iii) Manhattan plots of (Jansen et al., 2019) and (Kunkle et al., 2019) genetic variants. Plots are focused at loci where sentinel non-coding SNPs overlap with peaks enriched in non-neuronal cell types (d. *INPP5D*, e. *BIN1*, f. *PICALM*); bottom: zoomed in versions of the genome browser tracks displayed on top. *INPP5D* locus: the sentinel SNP rs10933431 overlaps with a peak that is enriched only in the microglial population; *BIN1* locus: the top two AD-associated SNPs based on GWAS *p*-value (rs4663105 and rs6733839) overlap with peaks enriched in both the microglial and OEG populations; *PICALM* locus: the top two SNPs (rs10792832 and rs3851179) also overlap with non-neuronal peaks. Regions of overlap are highlighted with a yellow box.

**Figure 3:** OEG display the strongest acetylation differences associated with Aβ pathology, including peaks annotated to genes associated with EOAD and LOAD risk (A). Heatmap displaying number of significantly hyperacetylated (log2fc > 0, FDR q < 0.05) and significantly hypoacetylated peaks (log2fc < 0, FDR q < 0.05) from each brain region, sex, and cell type-specific contrast (B) left: Heatmap of normalized acetylation levels at 1962 H3K27ac peaks that were significantly hypoacetylated in AD female hippocampus OEG samples. Rows represent the 1,962 DARs and columns represent hippocampal OEG samples. Aβ load for each sample is indicated at the top of the heatmap. right: A heatmap of the 1,962 peaks in male hippocampal OEG samples is included for comparison. DARs annotated to EOAD and LOAD risk genes are labeled in red and black, respectively. Peaks near *STMN4* and *MYRF* are annotated in green (C). Heatmap of the 1,029 H3K27ac peaks that were significantly hyperacetylated in AD dlPFC OEG samples. Peaks annotated to EOAD and LOAD risk genes are labeled in red and black, respectively. The *ADAMTS18* promoter-proximal peak is annotated in green (D). Distance to TSS distribution of (i) 1,962 OEG female hippocampus hypoacetylated DARs, (ii) 1,029 OEG dlPFC hyperacetylated DARs and (iii) the full consensus set of peaks (E). Enrichment heatmap of top gene ontology terms for 6 peak sets (1) 1,962 OEG female hippocampus hypoacetylated DARs (2) 1,029 OEG dlPFC hyperacetylated DARs (3) all other Aβ associated DARs (4) neuron, (5) microglia, and (6) OEG cell type-specific hyperacetylated peaks. Color intensity represents hypergeometric fold enrichment in number of peaks over background (full consensus peak set), ∗ indicates FDR *q* < 0.05, ∗∗ indicates FDR *q* < 0.01.

**Figure 4:** EOAD and LOAD risk genes exhibit epigenomic and transcriptomic perturbations in oligodendrocytes (A–I). Genome browser tracks displaying average H3K27ac signal in OEG samples from subjects with and without Aβ load (yellow and blue tracks, respectively). Regions displayed include EOAD and LOAD risk loci, as well as differentially acetylated regions near *ADAMTS18* and *MYRF* (J). RT-qPCR of select genes annotated to DARs identified in AD OEG female hippocampus. Panel shows violin plots of gene expression measured by RT-qPCR in hippocampal Olig2+ nuclei collected from an independent cohort of AD and non-AD subjects. q-values for differential expression between high Aβ and low+mid Aβ subjects are indicated on top for each gene. Correction was applied across the 9 tests (K) left panel: comparison with existing snRNA-seq from AD dlPFC (Mathys et al., 2019) reveals an average increase in gene expression near hyperacetylated regions in OEG dlPFC. Violin plots depict log2fc values from differential expression analysis between AD and non-AD subjects in oligodendrocytes (Mathys et al., 2019). These log2fc values are derived from 500 genes annotated to the OEG dlPFC hyperacetylated DARs that reside in putative promoters (<5kb from TSS). Log2fc violin plots are shown for two different contrasts performed in Mathys, Valderrain et al.: no pathology vs. pathology and no pathology vs. early pathology. *Q*-values from *t*-tests (null hypothesis: mean log2fc = 0, alternate hypothesis: mean log2fc > 0) are reported for the two violin plots. Correction was applied across the two tests. right panel: Specific genes associated with OEG dlPFC hyperacetylated DARs display increased transcription in AD. Individual log2fc values are shown. TSS distance cutoffs were not used for this right panel. FDR *q*-values from the differential expression analysis for each gene are also provided for both contrasts.

**SUPPLEMENTARY FIGURE 1:** Heatmap of subject information and standard ENCODE quality metrics for all ChIP-Seq samples. Sample quality information is also summarized in Supplementary Table 2.

**SUPPLEMENTARY FIGURE 2:** Jitter plots of overall amyloid load, tangles, global cognition score (cogn_global_lv), consensus cognitive diagnosis (cogdx), Braak stage (braaksc), CERAD score (ceradsc), age at death, years of education (educ), postmortem interval (pmi), brain weight (brainwt), brain pH (ph), and sample RSCs for dlPFC subjects.

**SUPPLEMENTARY FIGURE 3:** Jitter plots of overall amyloid load, tangles, global cognition score (cogn_global_lv), consensus cognitive diagnosis (cogdx), Braak stage (braaksc), CERAD score (ceradsc), age at death, years of education (educ), postmortem interval (pmi), brain weight (brainwt), brain pH (ph), and sample RSCs for hippocampal subjects.

**SUPPLEMENTARY FIGURE 4**: Gating strategy for nuclei sorting.

**SUPPLEMENTARY FIGURE 5:** Sorting of neuronal, microglial, and OEG nuclei from dlPFC of subjects with and without AD.

**SUPPLEMENTARY FIGURE 6:** Sorting of neuronal, microglial, and OEG nuclei from hippocampus of subjects with and without AD.

**SUPPLEMENTARY FIGURE 7:** Principal Components Analysis (PCA) analysis plots show all H3K27ac samples projected onto the top two principal components based on explained variance. Samples are colored by (A) brain region (B) sex (C) cell type, and (D) Aβ load. PC1 separates NeuN+ samples from Pu.1+ and NeuN-/Pu.1- samples and PC2 separates Pu.1+ samples from NeuN-/Pu.1- samples.

**SUPPLEMENTARY FIGURE 8:** Violin plot showing variance explained by each covariate included in variance partition analysis.

**SUPPLEMENTARY FIGURE 9:** Cell type composition of sorted H3K27ac ChIP-seq populations. H3K27ac signal at peaks near 15 cell type-specific gene lists annotated in (Habib et al., 2017) were used to assess cell type enrichment (A). t-statistic showing enrichment of a given cell type cluster in one sorted population over the other two non-focal sorted populations. Mean log2FC of signal was computed at H3K27ac peaks near the promoters ( < 5 kb distance to transcription start site) of the 15 cell type-specific marker gene sets and a *t*-test was used to compute whether the mean log2FC is greater than 0.5 (∼1.4-fold change). Value inside box represents Benjamini Hochberg FDR corrected q-value (-log10-transformed) for the *t*-test. Correction was applied across all 45 tests (B). Same as panel a., but without a TSS distance filter (C). Mean log2fc (focal population/non-focal populations) for peaks near 15 cluster markers at the individual sample level and for sample aggregates (without a TSS distance filter). Abbreviated labels for the single nucleus RNA-seq clusters are defined: exPFC, glutamatergic neurons from the PFC; GABA, GABAergic interneurons; exCA1/3, pyramidal neurons from the hippocampus CA region; exDG, granule neurons from the hippocampus dentate gyrus region; ASC, astrocytes; MICROGLIA, microglia; OLIGO, oligodendrocytes; OPC, oligodendrocyte precursor cells; NSC, neuronal stem cells; END, endothelial cells.

**SUPPLEMENTARY FIGURE 10:** Independent permutation test method confirms that AD-associated 2411 variants from the two large GWAS studies (Jansen et al., 2019; Kunkle et al., 2019) colocalize with peaks enriched in microglia relative to peaks enriched in OEG and neurons, suggesting that microglial gene regulation may influence predisposition toward LOAD (A). Plot of fold change (log2 transformed) in the number of AD associated SNPs ((Kunkle et al., 2019); GWAS *p*-value < 1e-3) overlapping focal foreground cell type specific peaks vs. the number of AD associated SNPs overlapping background set of peaks in all three cell types; permutation test controls for LD and minor allele frequency, Benjamini Hochberg FDR corrected q-values across the three tests for colocalization are indicated above each bar (B). same as (A). but using the Jansen et al GWAS.

**SUPPLEMENTARY FIGURE 11**: SNPs associated with Schizophrenia from a large GWAS study (Consortium, 2014) colocalize with peaks enriched in neurons relative to peaks enriched in OEG and microglia, suggesting neuronal gene regulation may influence predisposition toward Schizophrenia. Plot shows the estimated stratified LD score regression coefficient for the three peak sets. Benjamini Hochberg FDR corrected q-values across the three tests are indicated above each bar.

**SUPPLEMENTARY FIGURE 12**: H3K27ac levels at 1962 hypoacetylated peaks in female hippocampus OEG show some correlation with RSC but not with other variables such as age at death, years of education and pmi (A,B). Read counts at 1962 hypoacetylated DARs show consistent decrease with an increase in overall Aβ (C–E). No relation can be observed between mean read count and variables such as age at death, years of education, and pmi.(F) RSC is correlated with 23.9% of peaks (FDR q < 0.05). The red X symbol represents information that couldn’t be collected in ROSMAP.

**SUPPLEMENTARY FIGURE 13:** H3K27ac levels at 1,029 hyperacetylated peaks in AD dlPFC OEG show correlations with RSC but not with other variables such as age at death, years of education, and pmi. (A,B). Read counts at 1,029 hyperacetylated DARs show consistent increase with an increase in overall Aβ (C–E). No relation can be observed between mean read count and variables such as age at death, years of education, pmi, (F). RSC is correlated with 94.3% of peaks (FDR q < 0.05).

**SUPPLEMENTARY FIGURE 14:** Multiple modules of the KEGG Alzheimer’s Disease Pathway are associated with dlPFC and hippocampal OEG DARs. This includes peaks near all three Aβ secretase complexes, *MAPT,* and all 5 modules involved in oxidative phosphorylation. Boxes colored in orange represent genes which contain an annotation to the 1,962 hypoacetylated peaks discovered in female hippocampus OEG. Boxes colored in maroon represent genes which contain an annotation to the 1,029 hyperacetylated peaks in dlPFC OEG. An average of orange and maroon is used to color boxes which represent genes present in both DAR sets.

**SUPPLEMENTARY FIGURE 15:** Network visualization of the clustered PPI network of genes annotated to peaks near the 1,962 DARs identified in OEG female hippocampus samples Only the top 8 clusters based on the greatest number of genes are shown. Clusters are annotated with their associated top gene ontology biological process terms enriched in a DAVID analysis(Sherman et al., 2022).

**SUPPLEMENTARY FIGURE 16:** Genome browser tracks depict average OEG H3K27ac signal from subjects with and without Aβ load (yellow and blue, respectively). Regions correspond to LOAD risk loci as well as differentially acetylated regions near *STMN4.* H3K27ac signal from female hippocampal OEG samples and dlPFC OEG samples are shown.

**SUPPLEMENTARY FIGURE 17:** mRNA from cell type specific nuclei are enriched for cell type specific marker genes. Olig2+ nuclei are enriched for MBP, NeuN+ nuclei are enriched for Reln, GFAP+ nuclei are enriched for GFAP, and Pu.1+ nuclei are enriched for C1qa. S.E.M. displayed.

**SUPPLEMENTARY FIGURE 18:** mRNA from Olig2+ nuclei do not display significant differences in quality between AD and non-AD subjects as measured by (A). RNA-quality number (RQN) and (B). DV200 (percentage of RNA fragments with > 200 nucleotides).

**SUPPLEMENTARY FIGURE 19:** Jitter plots of overall amyloid load, tangles, global cognition score (cogn_global_lv), consensus cognitive diagnosis (cogdx), Braak stage (braaksc), CERAD score (ceradsc), age at death, years of education (educ), postmortem interval (pmi), brain weight (brainwt), and brain pH (ph) for subject used for RT-qPCR profiling.

**SUPPLEMENTARY FIGURE 20:** Network visualization of the clustered PPI network of genes annotated to peaks near the 1,029 hyperacetylated DARs identified in OEG dlPFC samples Only the top 7 clusters based on the greatest number of genes are shown. Clusters are annotated with their associated top gene ontology biological process (GO BP) terms enriched in a DAVID analysis (Sherman et al., 2022).The last two clusters did not enrich for any GO BP term in DAVID analysis.

**SUPPLEMENTARY FIGURE 21:** Summary of peaks displaying differential H3K27ac with age in microglia (A). Distribution of age-associated log2 fold changes indicates age-associated changes in H3K27ac levels are enriched in microglia. (B) Heatmap of variance stabilized read counts representing acetylation levels for the 444 peaks that display age-associated differential H3K27ac in microglia. (C) Genome browser tracks displaying age-associated hyperacetylated peaks near *APP* and *LRRTM3.* (D) Distribution of transcription log2 fold changes for 307 genes annotated to 391 age-associated hypoacetylated peaks and 50 genes annotated to 53 age-associated hyperacetylated peaks. Gene expression data was sourced from (Olah et al., 2018). Two dashed black vertical lines represent mean transcription log2FC for genes near age-associated hypoacetylated and age-associated hyperacetylated peaks (left and right, respectively). The red vertical dashed line indicates log2FC = 0, which represents no difference in transcription. *T*-test *p*-values are indicated for three comparisons, from left to right: (blue) hypoacetylated mean vs. 0; (green) hyperacetylated vs. hypoacetylated means; (orange) hyperacetylated mean vs. 0.

**SUPPLEMENTARY FIGURE 22:** Replication analysis for OEG DARs across brain regions (A). Scatter plot of log2fc values in OEG female hippocampus samples vs. log2fc values in OEG female dlPFC samples for all peaks that pass independent filtering criteria in DESeq2 (B). same as A but for only the 1,962 hypoacetylated DARs and 8 hyperacetylated DARs in hippocampus OEG female samples. As observed, the log2fc values display little correlation and directionality of effect is also not consistent. (C) Scatter plot of log2fc values in OEG female hippocampus samples vs. log2fc values in OEG female dlPFC samples for all peaks that pass independent filtering criteria in DESeq2 (D). Same as (C) but for only the 1,029 hyperacetylated DARs in OEG dlPFC samples. As observed, the log2fc values display little correlation and directionality of effect is also not consistent.

**REFERENCES:**

Consortium, S. W. G. of the P. G. (2014). Biological insights from 108 schizophrenia-associated genetic loci. *Nature* 511, 421–427. doi: 10.1038/nature13595.

Habib, N., Avraham-Davidi, I., Basu, A., Burks, T., Shekhar, K., Hofree, M., et al. (2017). Massively parallel single-nucleus RNA-seq with DroNc-seq. *Nat. Methods* 14, 955–958. doi: 10.1038/nmeth.4407.

Jansen, I. E., Savage, J. E., Watanabe, K., Bryois, J., Williams, D. M., Steinberg, S., et al. (2019). Genome-wide meta-analysis identifies new loci and functional pathways influencing Alzheimer’s disease risk. *Nat. Genet.*, 1. doi: 10.1038/s41588-018-0311-9.

Kunkle, B. W., Grenier-Boley, B., Sims, R., Bis, J. C., Damotte, V., Naj, A. C., et al. (2019). Genetic meta-analysis of diagnosed Alzheimer’s disease identifies new risk loci and implicates Aβ, tau, immunity and lipid processing. *Nat. Genet.* 51, 414–430. doi: 10.1038/s41588-019-0358-2.

Mathys, H., Davila-Velderrain, J., Peng, Z., Gao, F., Mohammadi, S., Young, J. Z., et al. (2019). Single-cell transcriptomic analysis of Alzheimer’s disease. *Nature* 570, 332–337. doi: 10.1038/s41586-019-1195-2.

Olah, M., Patrick, E., Villani, A.-C., Xu, J., White, C. C., Ryan, K. J., et al. (2018). A transcriptomic atlas of aged human microglia. 9. doi: 10.1038/s41467-018-02926-5.

Sherman, B. T., Hao, M., Qiu, J., Jiao, X., Baseler, M. W., Lane, H. C., et al. (2022). DAVID: a web server for functional enrichment analysis and functional annotation of gene lists (2021 update). *Nucleic Acids Res.* 50. doi: 10.1093/NAR/GKAC194.
