# Supplementary figures and images for: Cell type-specific histone acetylation profiling of Alzheimer’s disease subjects and integration with genetics
Source: Front Mol Neurosci. 2023 Jan 6;15:948456. doi: 10.3389/fnmol.2022.948456 (PMC9853565; doi:10.3389/fnmol.2022.948456)

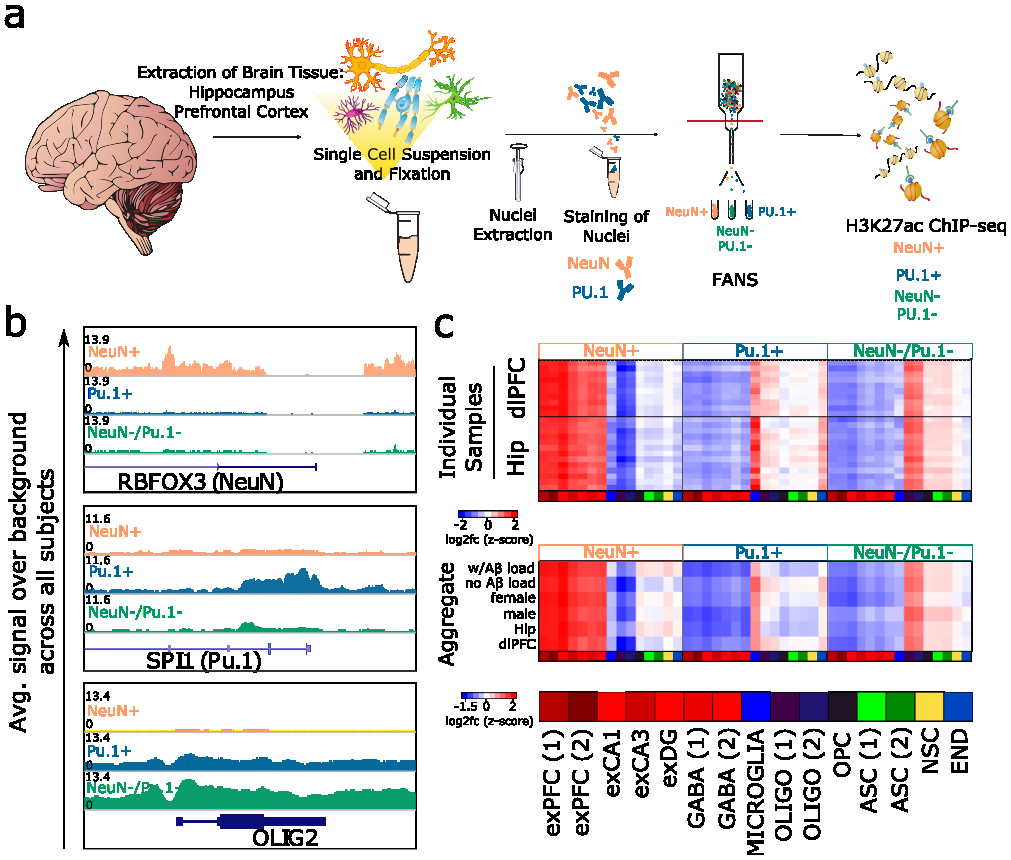

Supplement: Supplementary file 14 [file Presentation_1.zip › Figure 1.tiff]

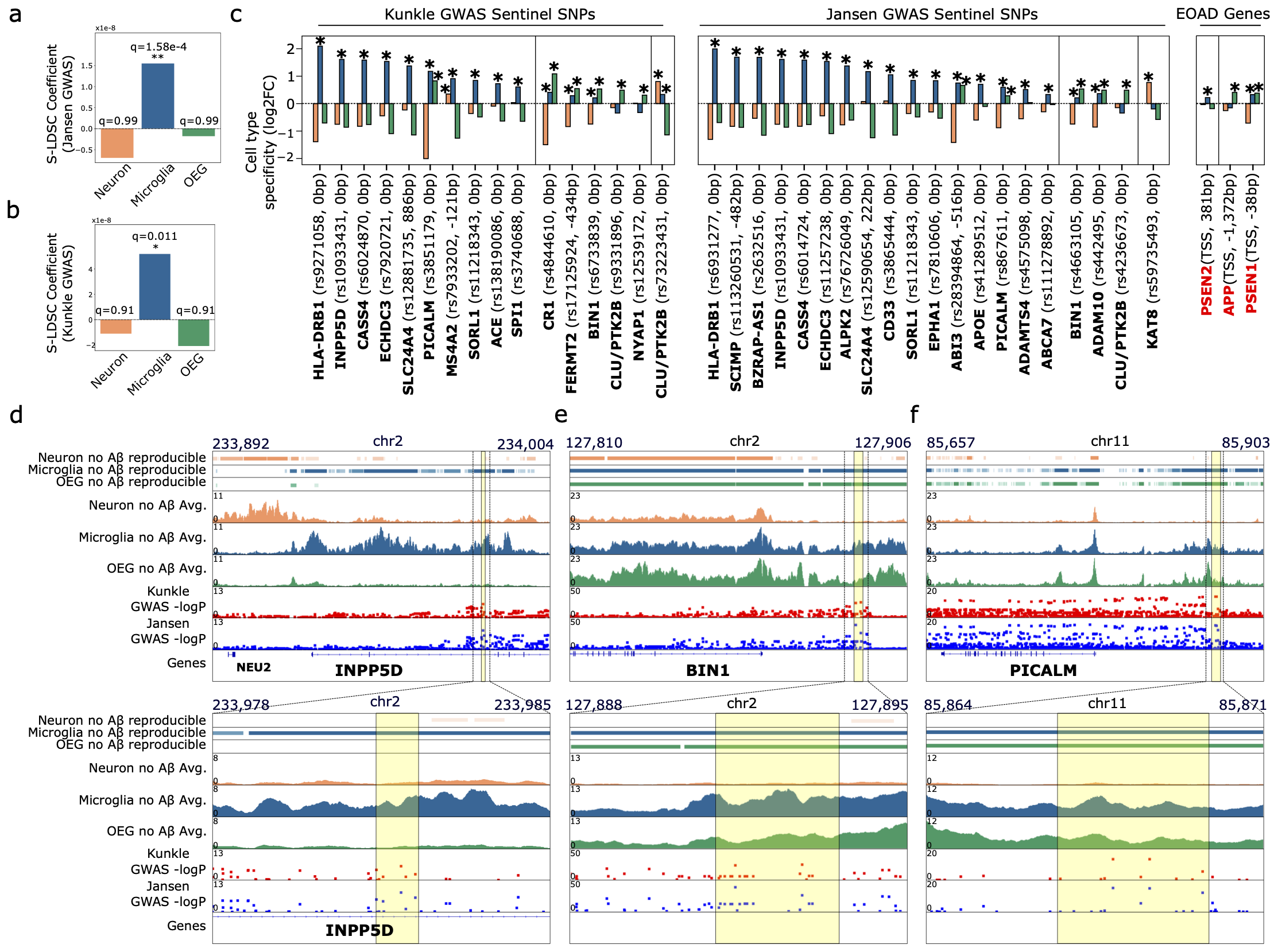

Supplement: Supplementary file 14 [file Presentation_1.zip › Figure 2.TIFF]

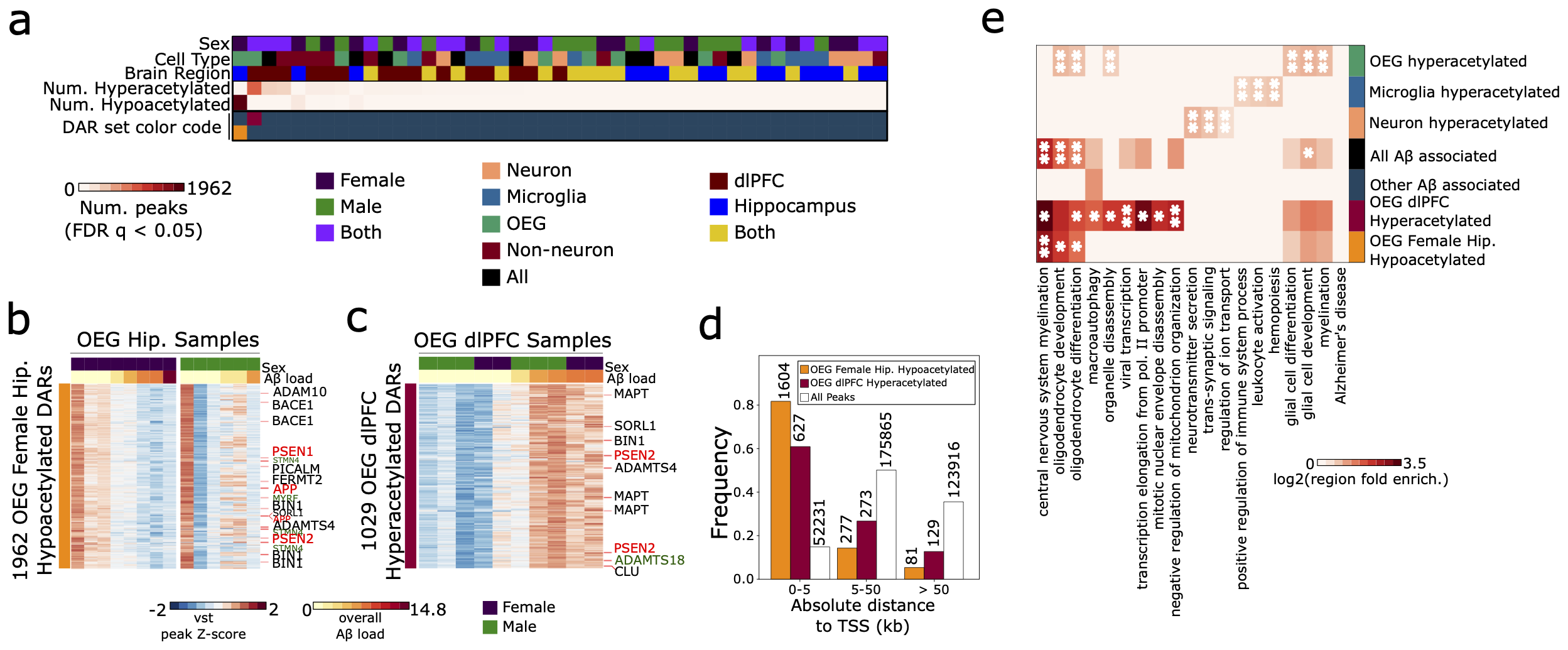

Supplement: Supplementary file 14 [file Presentation_1.zip › Figure 3.TIFF]

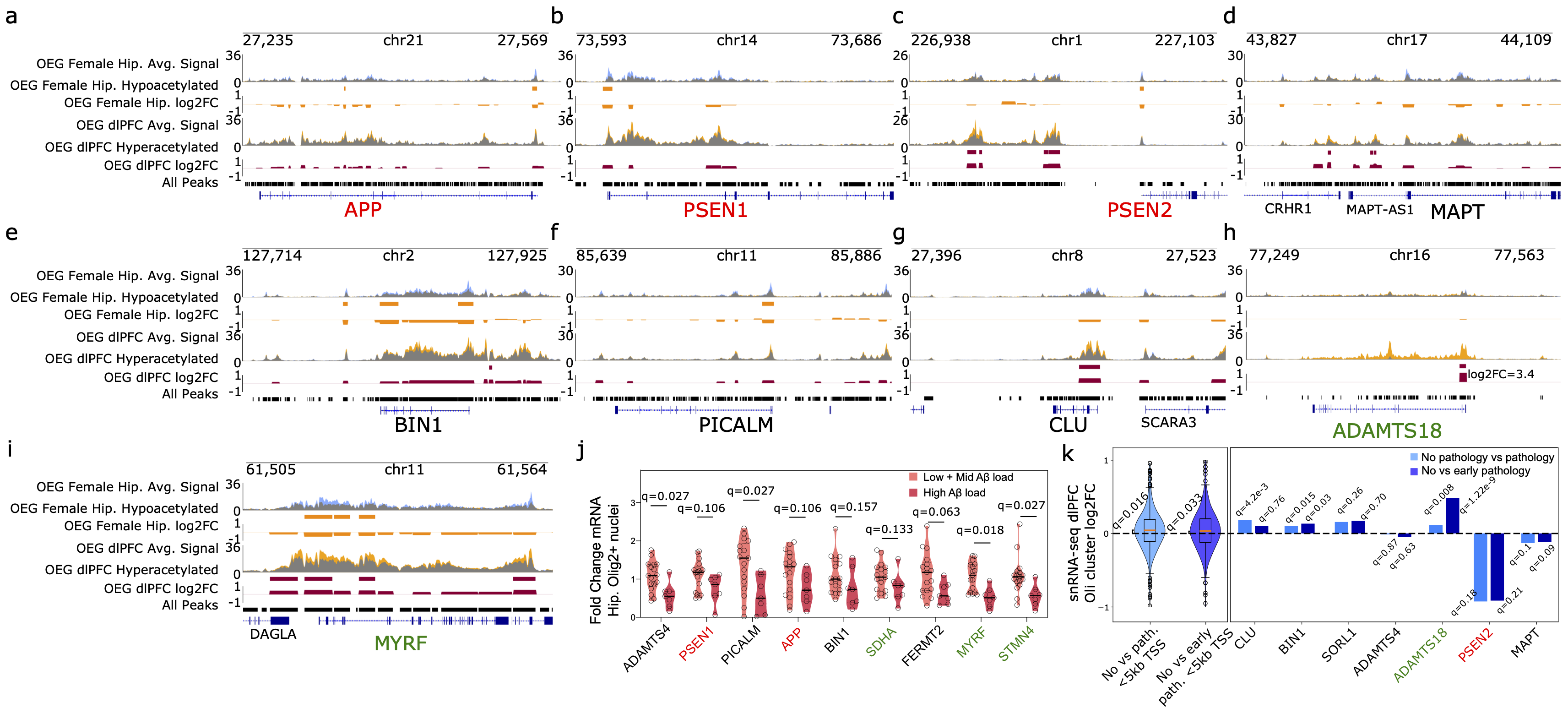

Supplement: Supplementary file 14 [file Presentation_1.zip › Figure 4.TIFF]

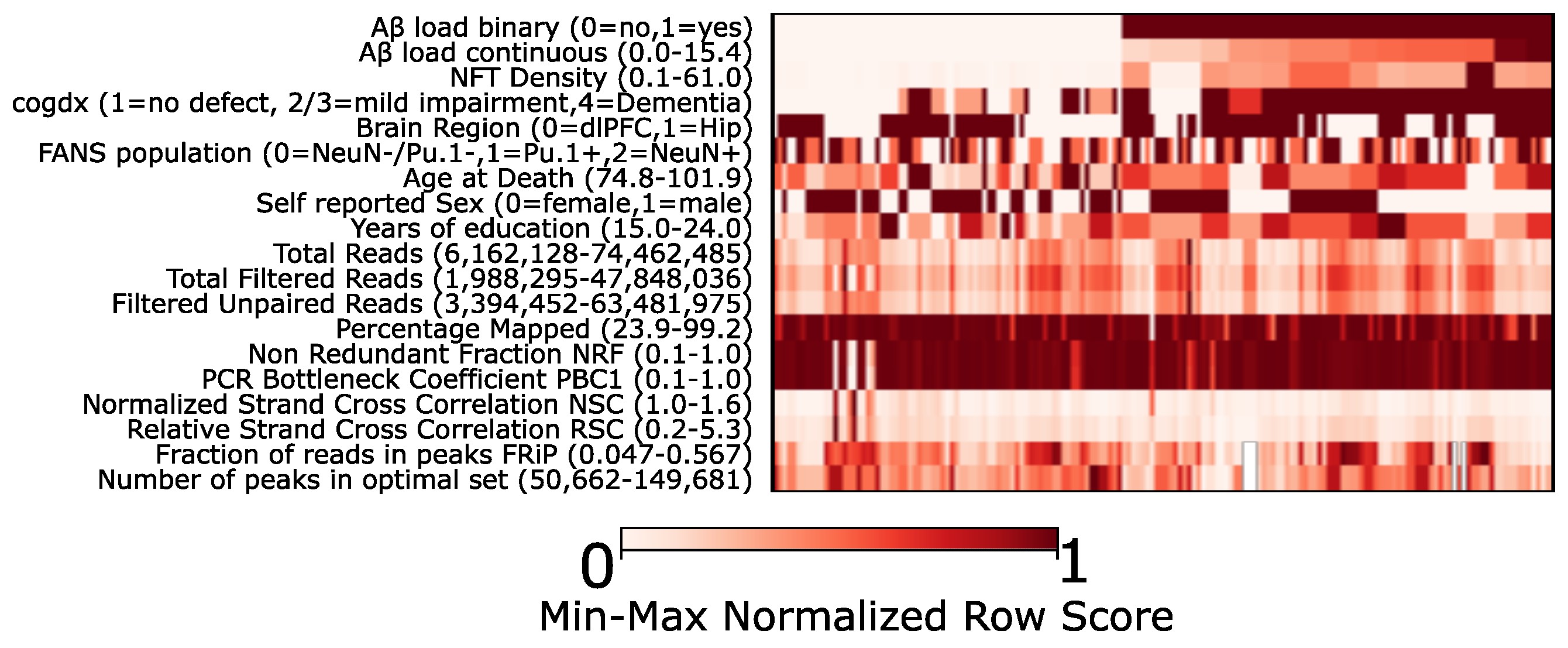

Supplement: Supplementary file 14 [file Presentation_1.zip › Supplementary Figure 1.jpg]

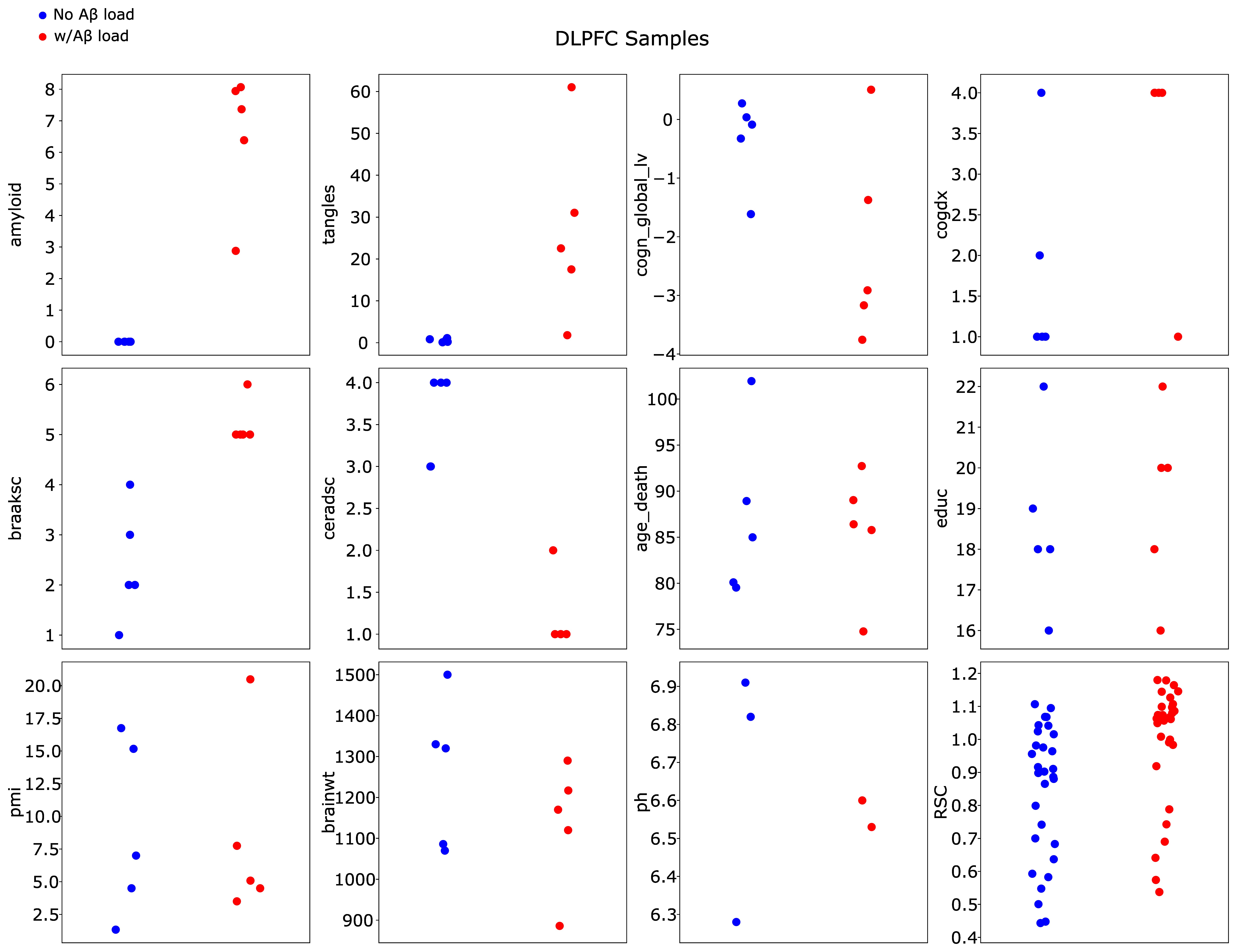

Supplement: Supplementary file 14 [file Presentation_1.zip › Supplementary Figure 2.jpg]

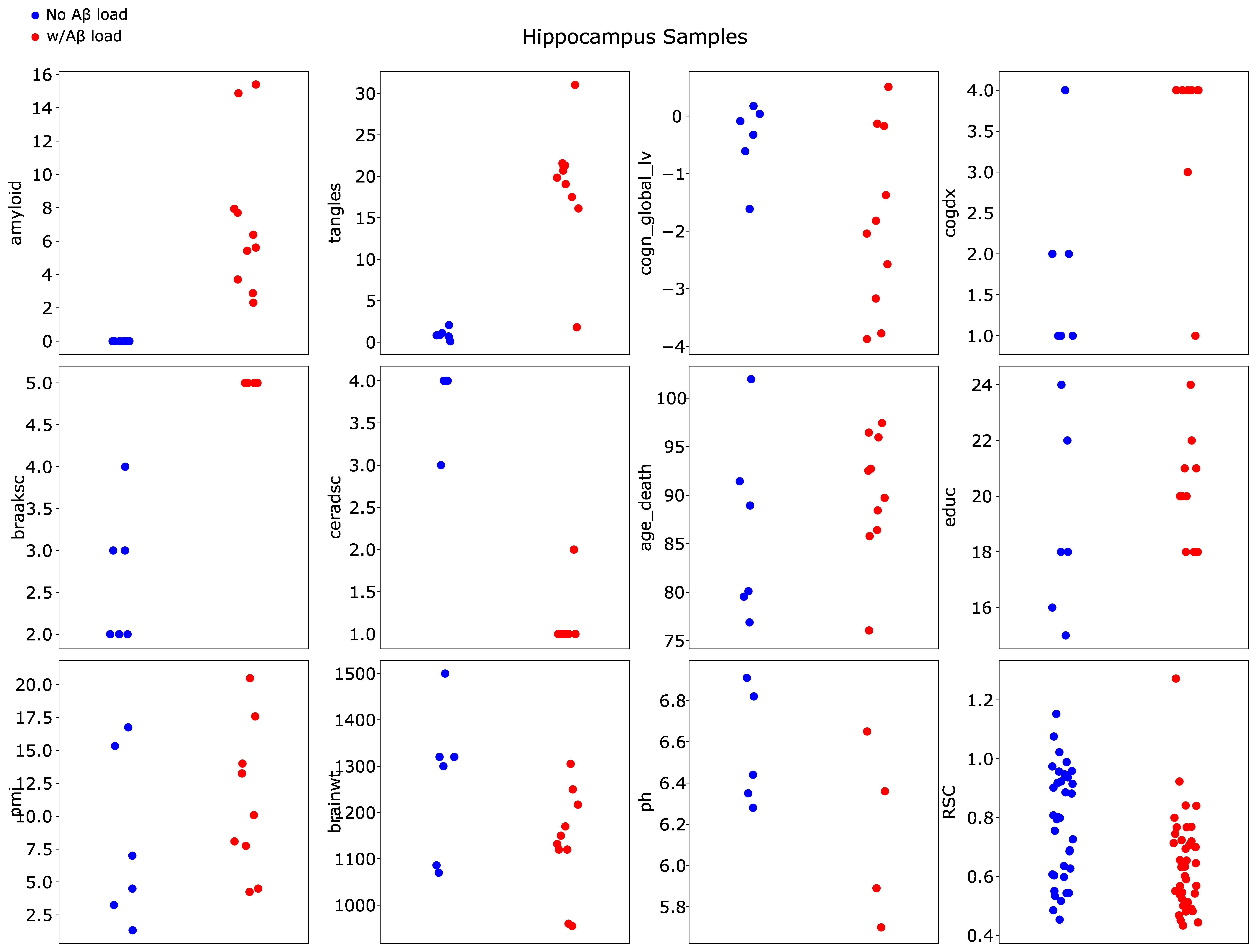

Supplement: Supplementary file 14 [file Presentation_1.zip › Supplementary Figure 3.jpg]

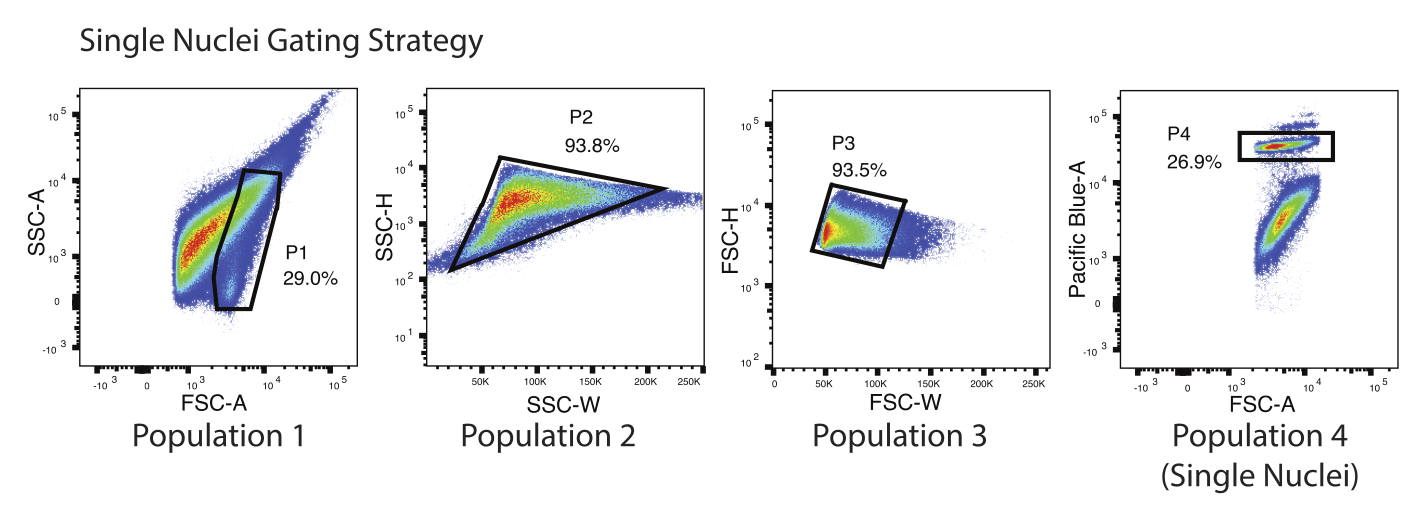

Supplement: Supplementary file 14 [file Presentation_1.zip › Supplementary Figure 4.TIFF]

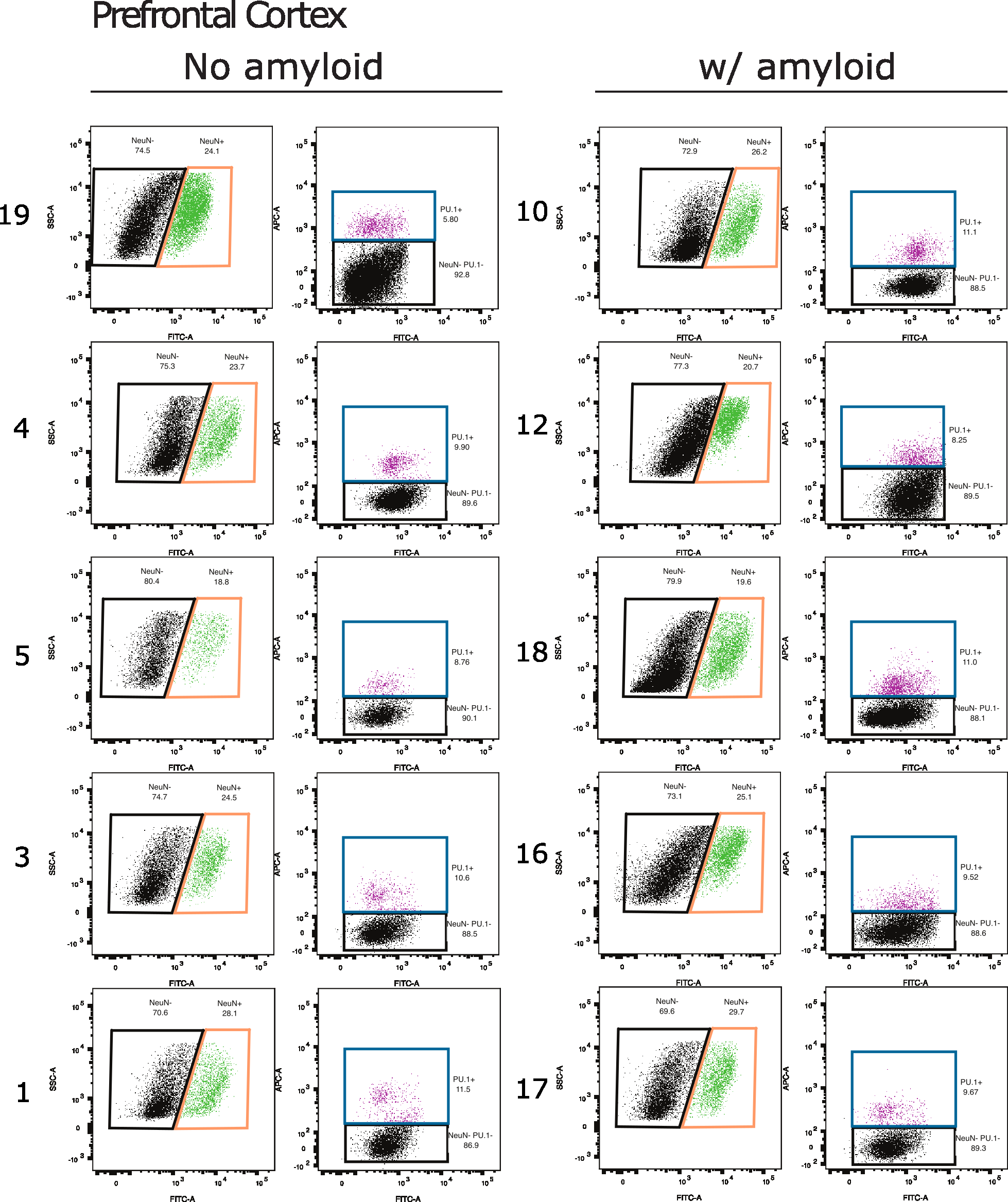

Supplement: Supplementary file 14 [file Presentation_1.zip › Supplementary Figure 5.TIFF]

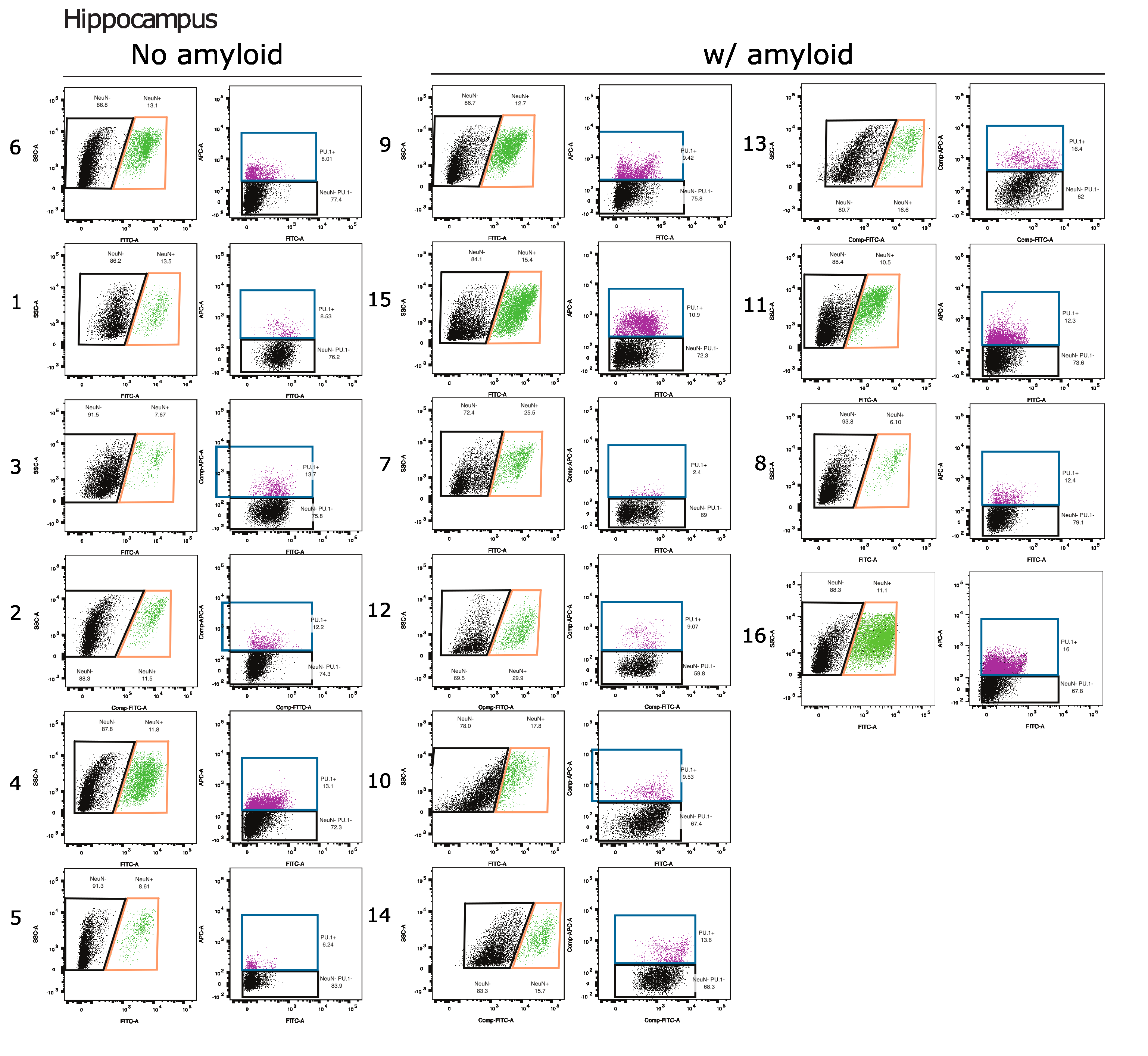

Supplement: Supplementary file 14 [file Presentation_1.zip › Supplementary Figure 6.TIFF]

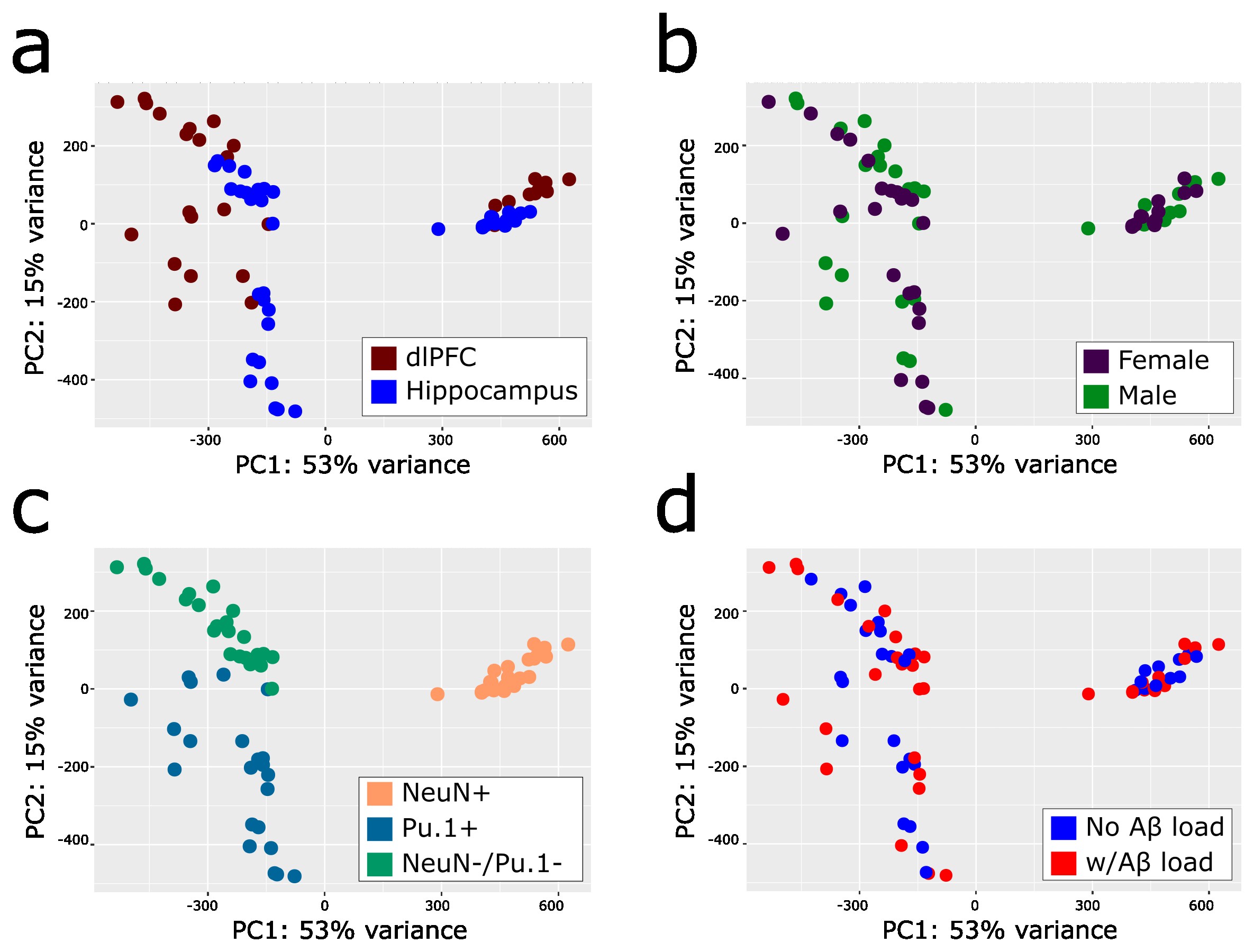

Supplement: Supplementary file 14 [file Presentation_1.zip › Supplementary Figure 7.jpg]

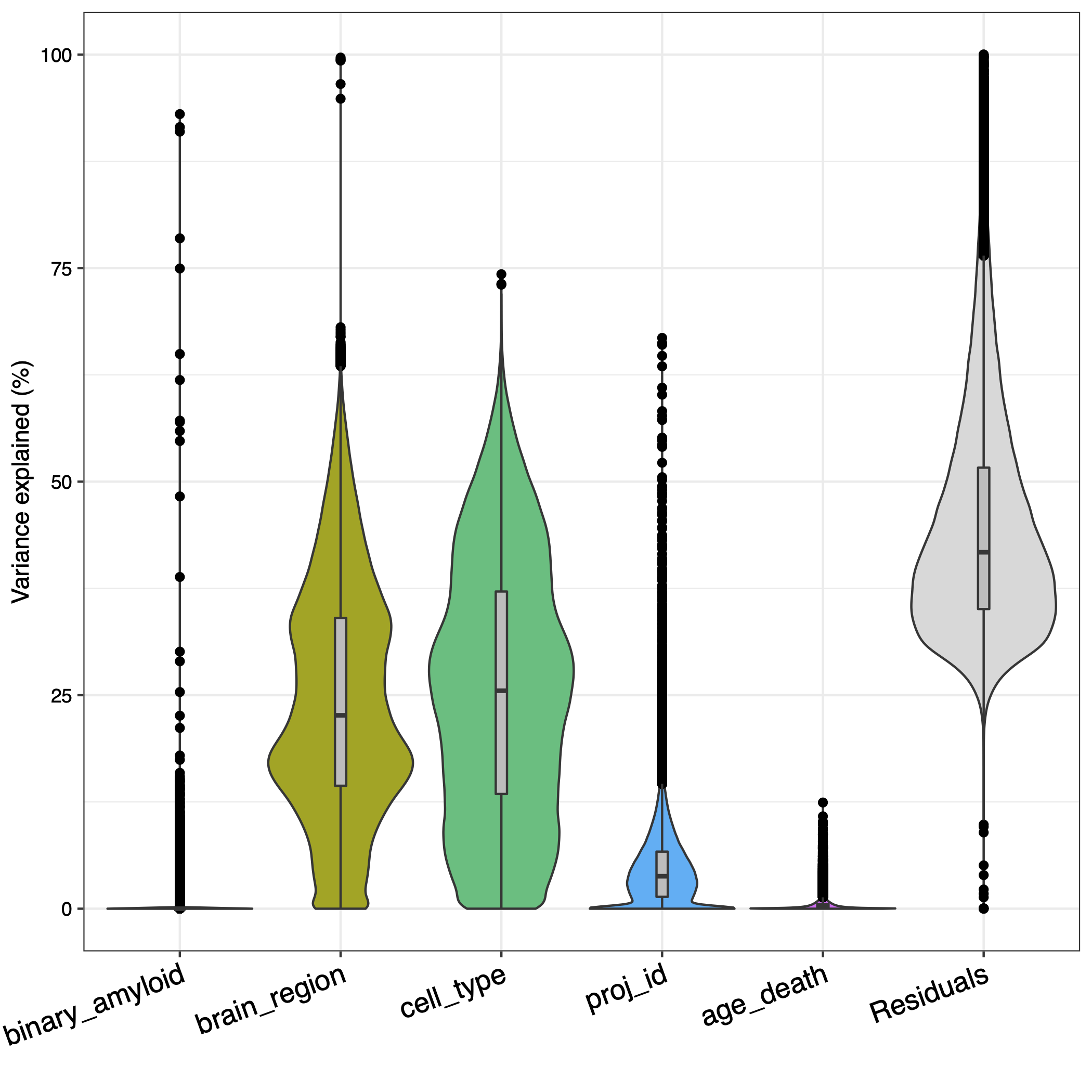

Supplement: Supplementary file 14 [file Presentation_1.zip › Supplementary Figure 8.TIFF]

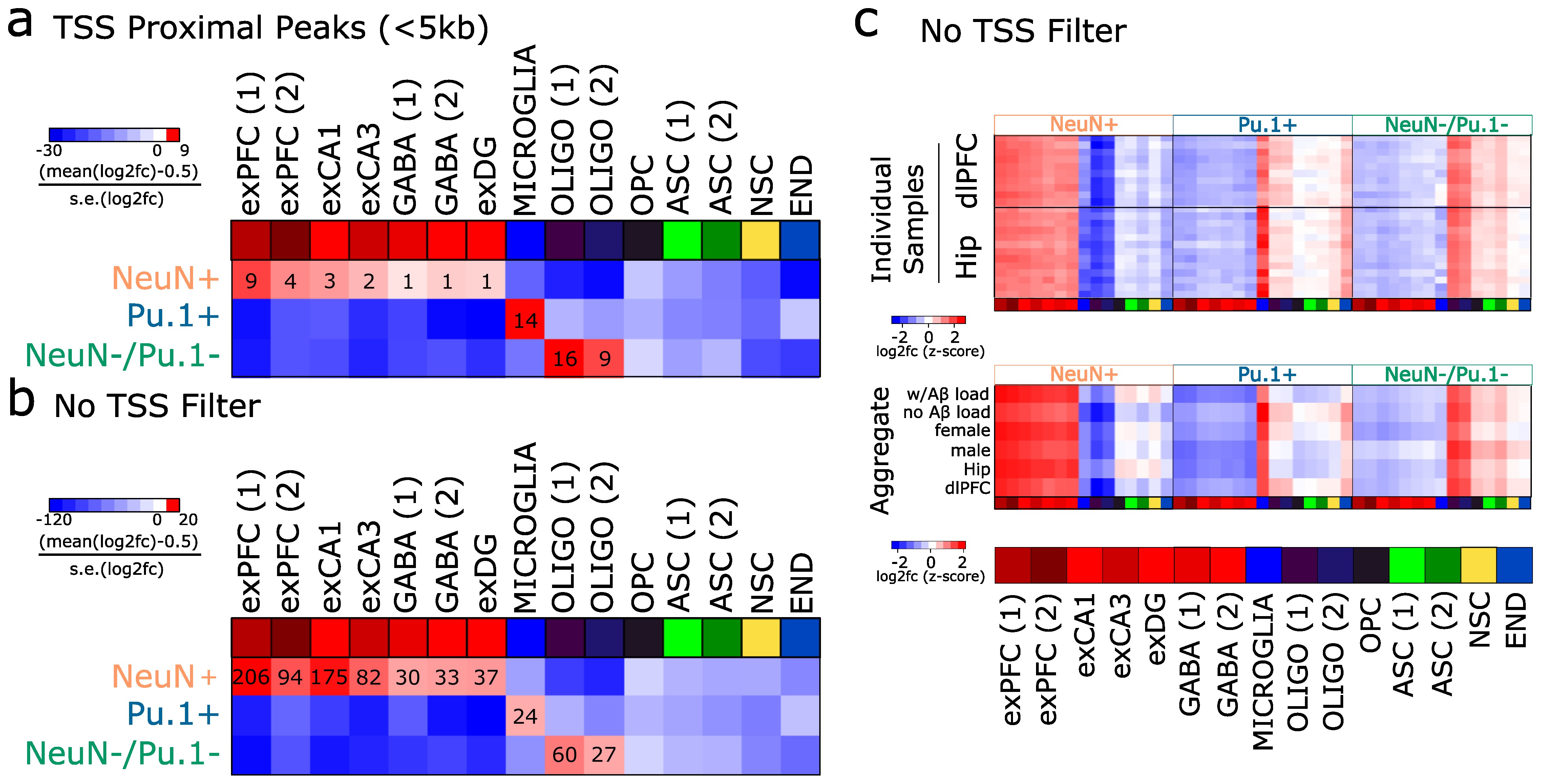

Supplement: Supplementary file 14 [file Presentation_1.zip › Supplementary Figure 9.jpg]

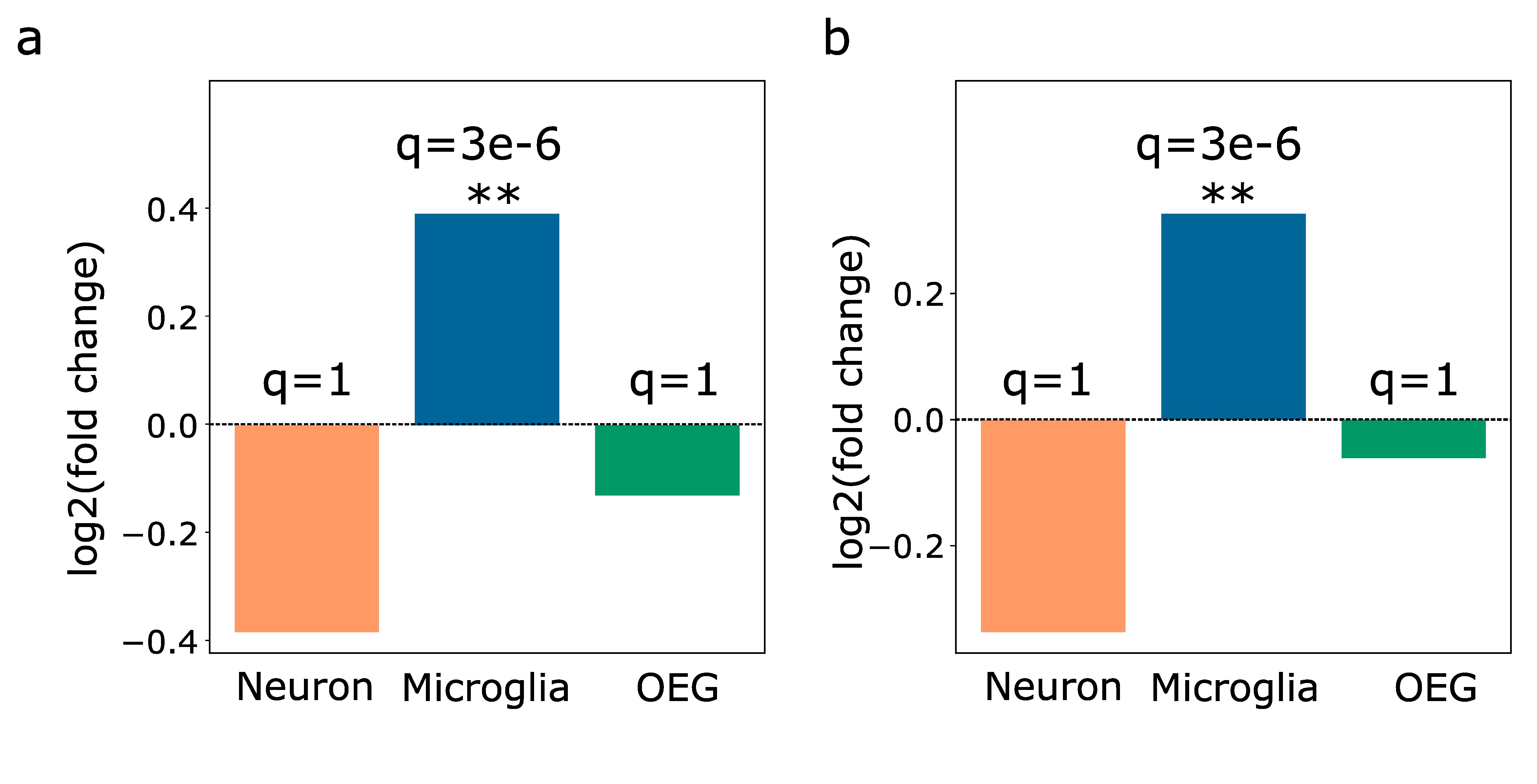

Supplement: Supplementary file 14 [file Presentation_1.zip › Supplementary Figure 10.jpg]

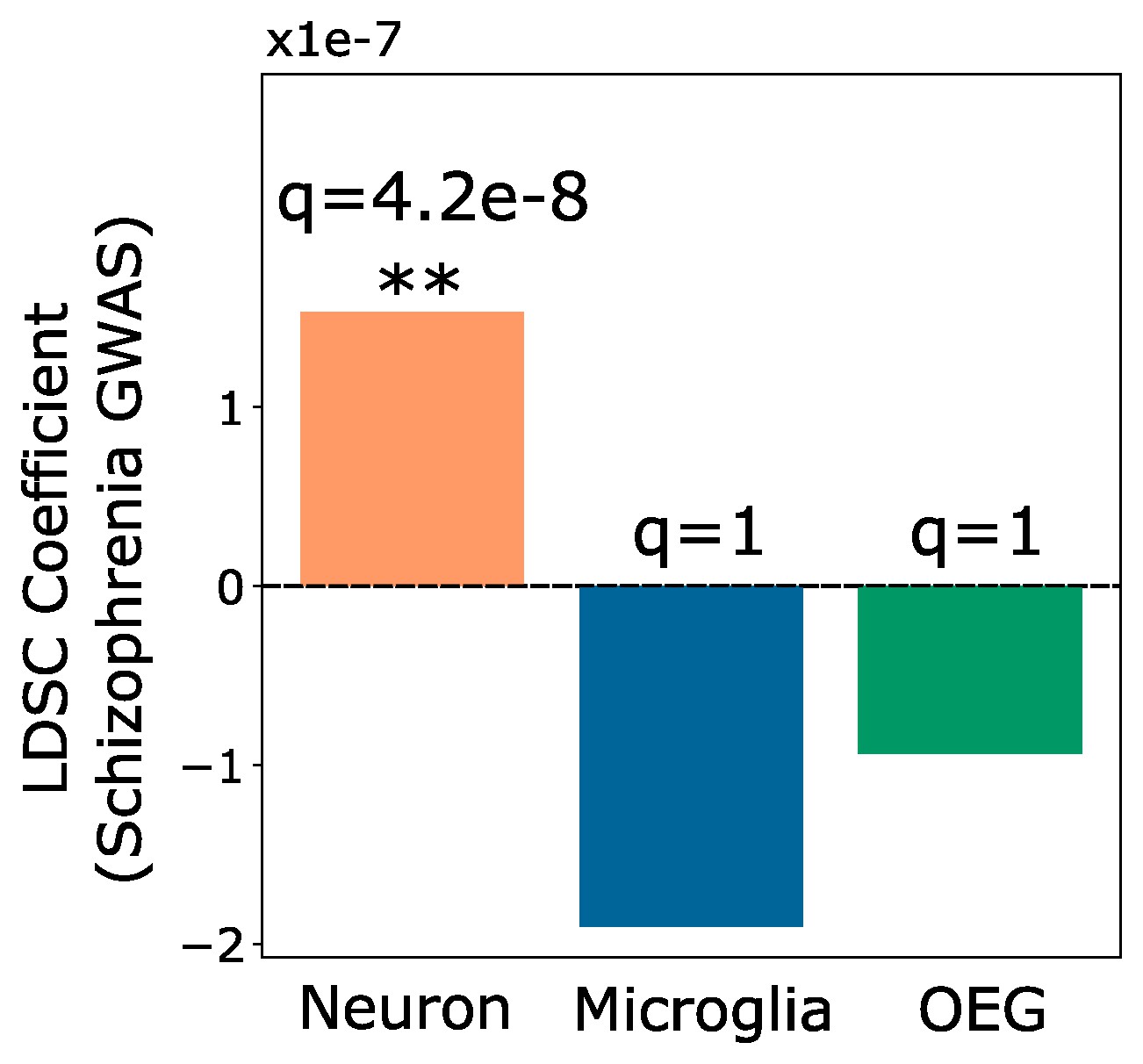

Supplement: Supplementary file 14 [file Presentation_1.zip › Supplementary Figure 11.jpg]

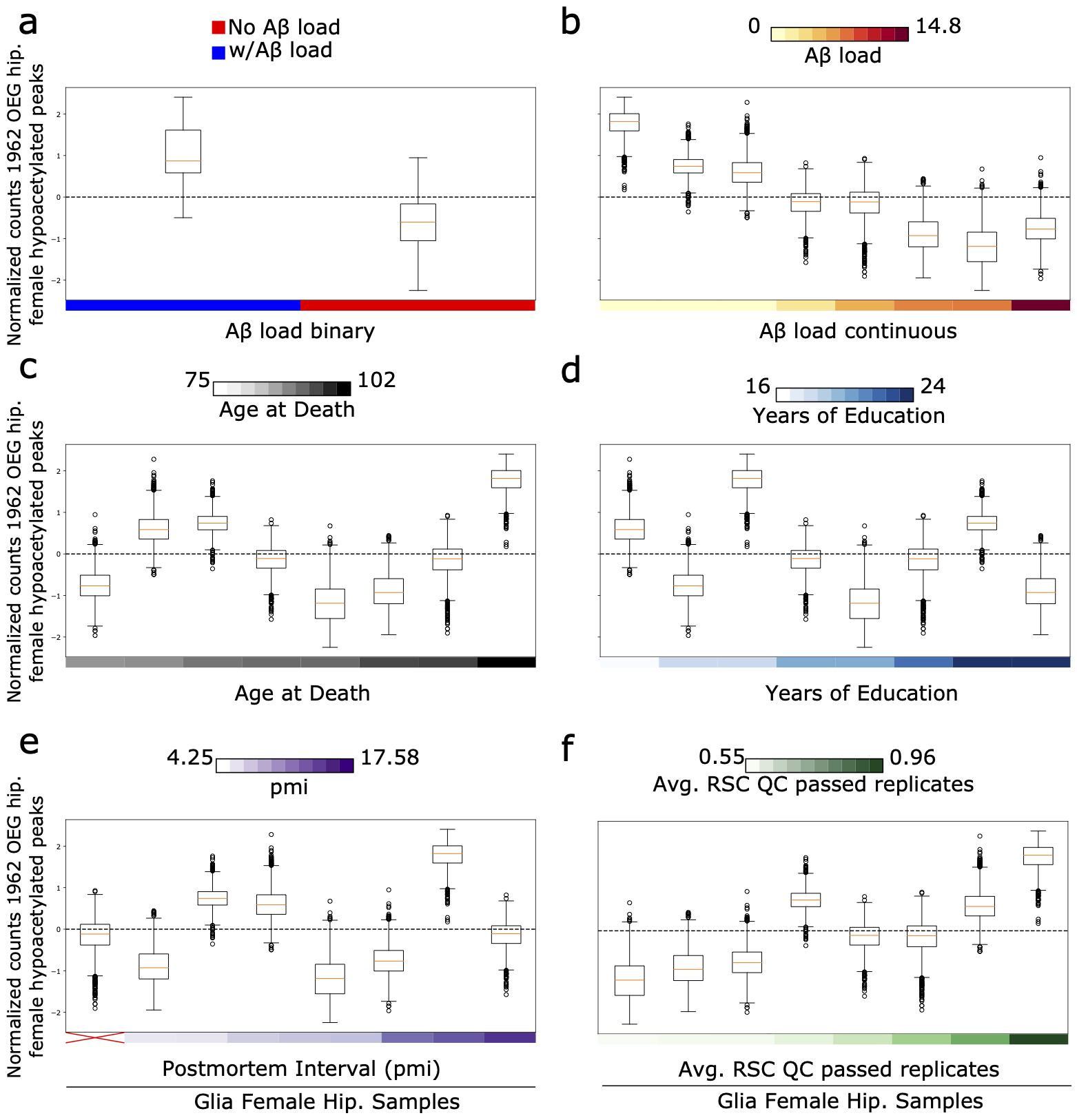

Supplement: Supplementary file 14 [file Presentation_1.zip › Supplementary Figure 12.TIFF]

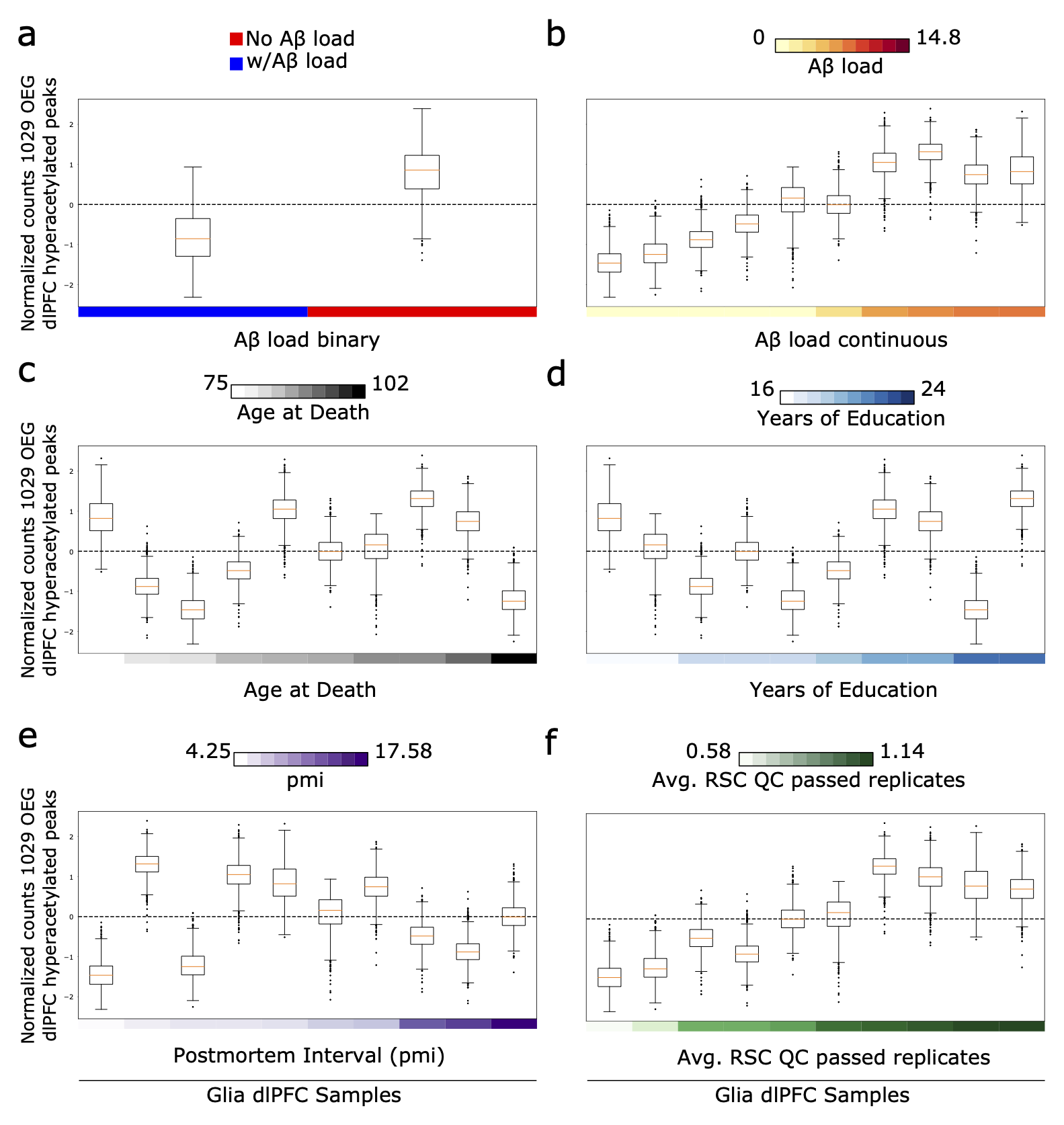

Supplement: Supplementary file 14 [file Presentation_1.zip › Supplementary Figure 13.TIFF]

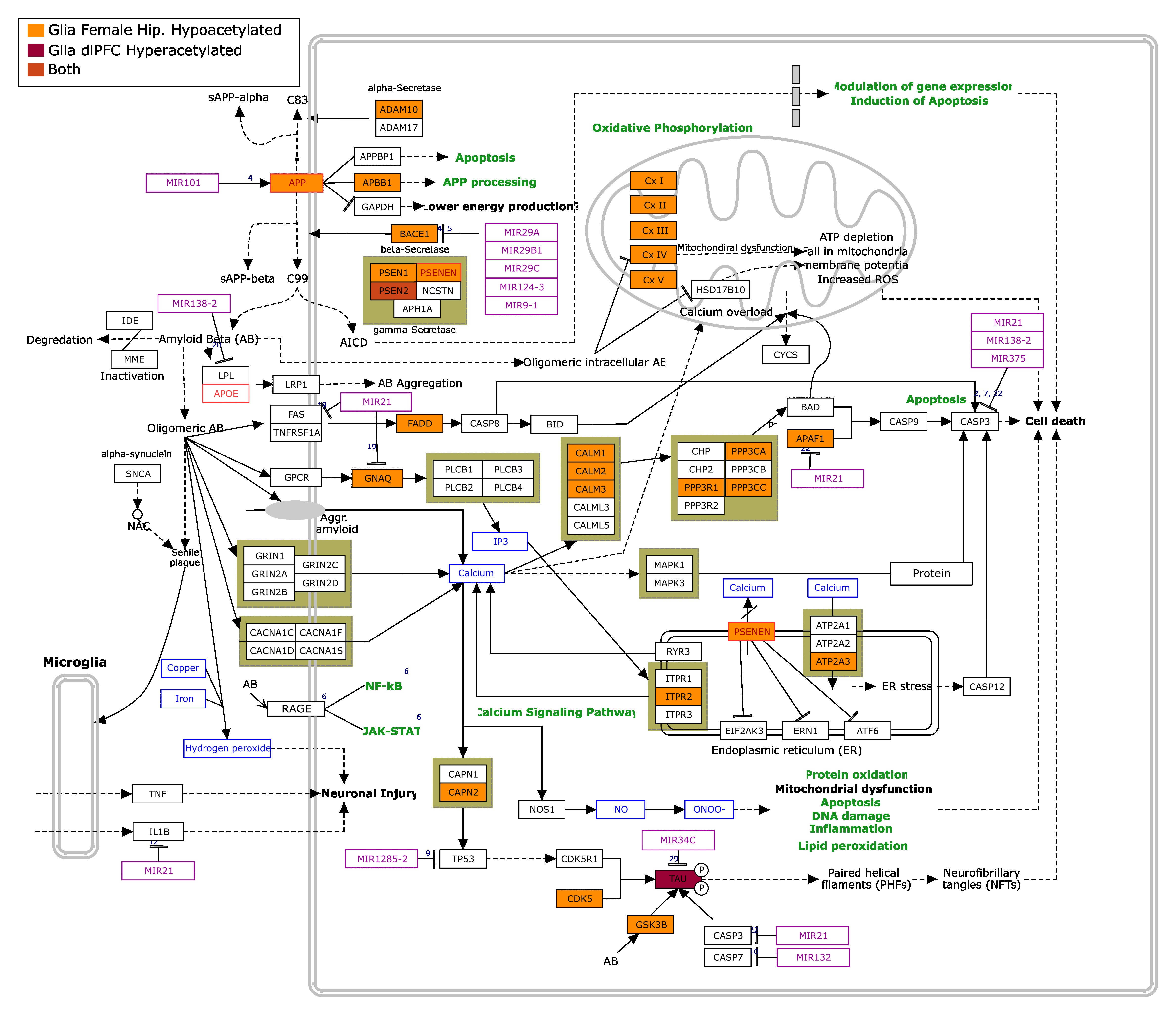

Supplement: Supplementary file 14 [file Presentation_1.zip › Supplementary Figure 14.jpg]

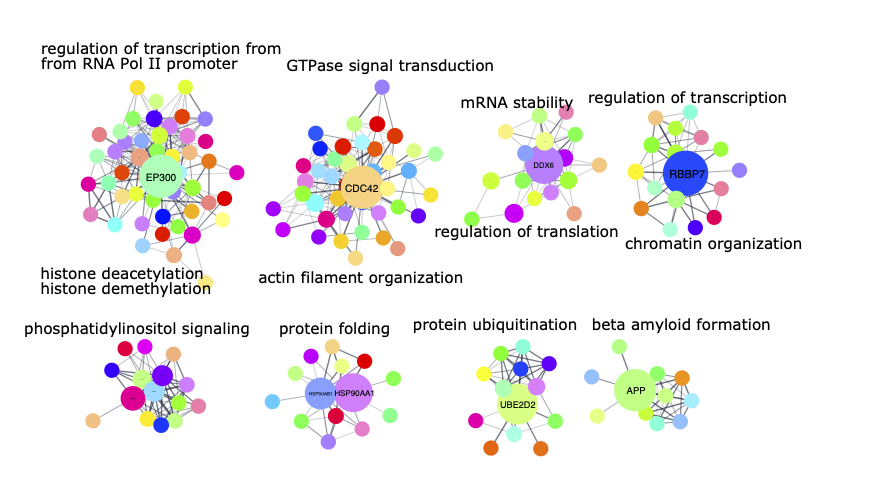

Supplement: Supplementary file 14 [file Presentation_1.zip › Supplementary Figure 15.TIFF]

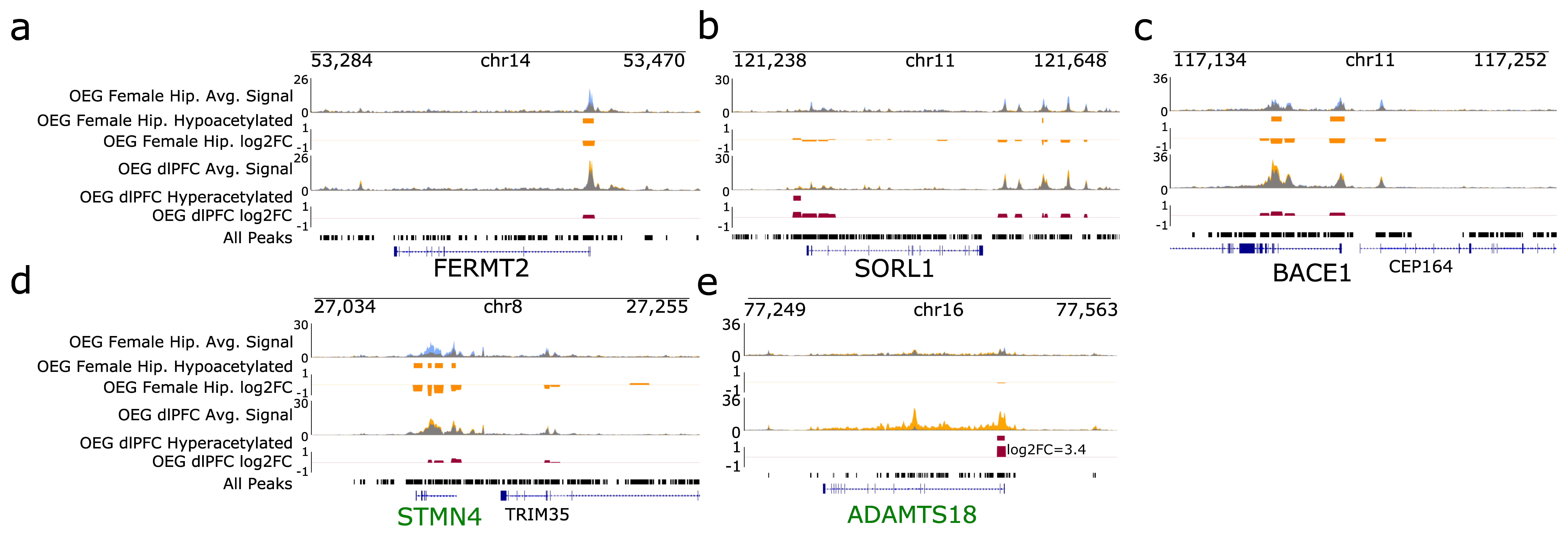

Supplement: Supplementary file 14 [file Presentation_1.zip › Supplementary Figure 16.jpg]

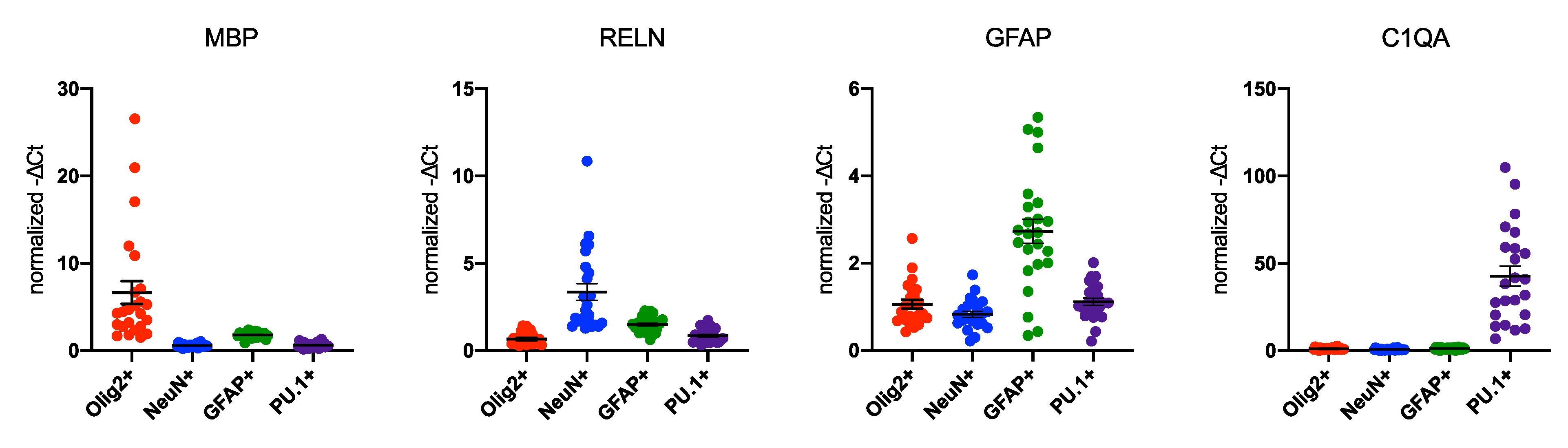

Supplement: Supplementary file 14 [file Presentation_1.zip › Supplementary Figure 17.jpg]

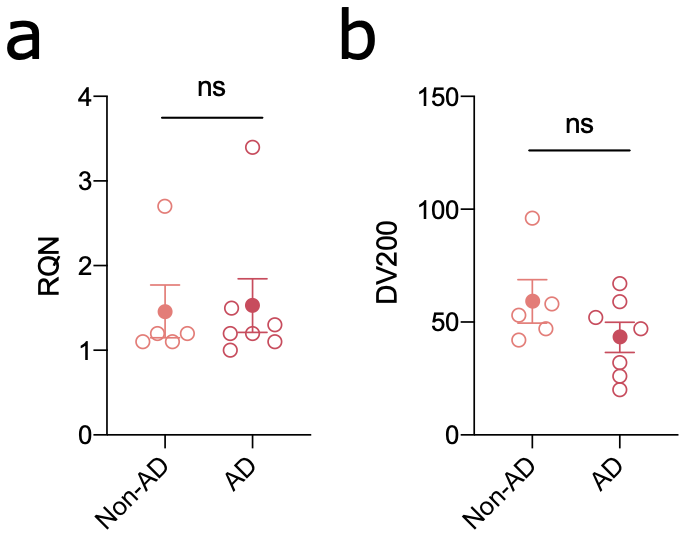

Supplement: Supplementary file 14 [file Presentation_1.zip › Supplementary Figure 18.TIFF]

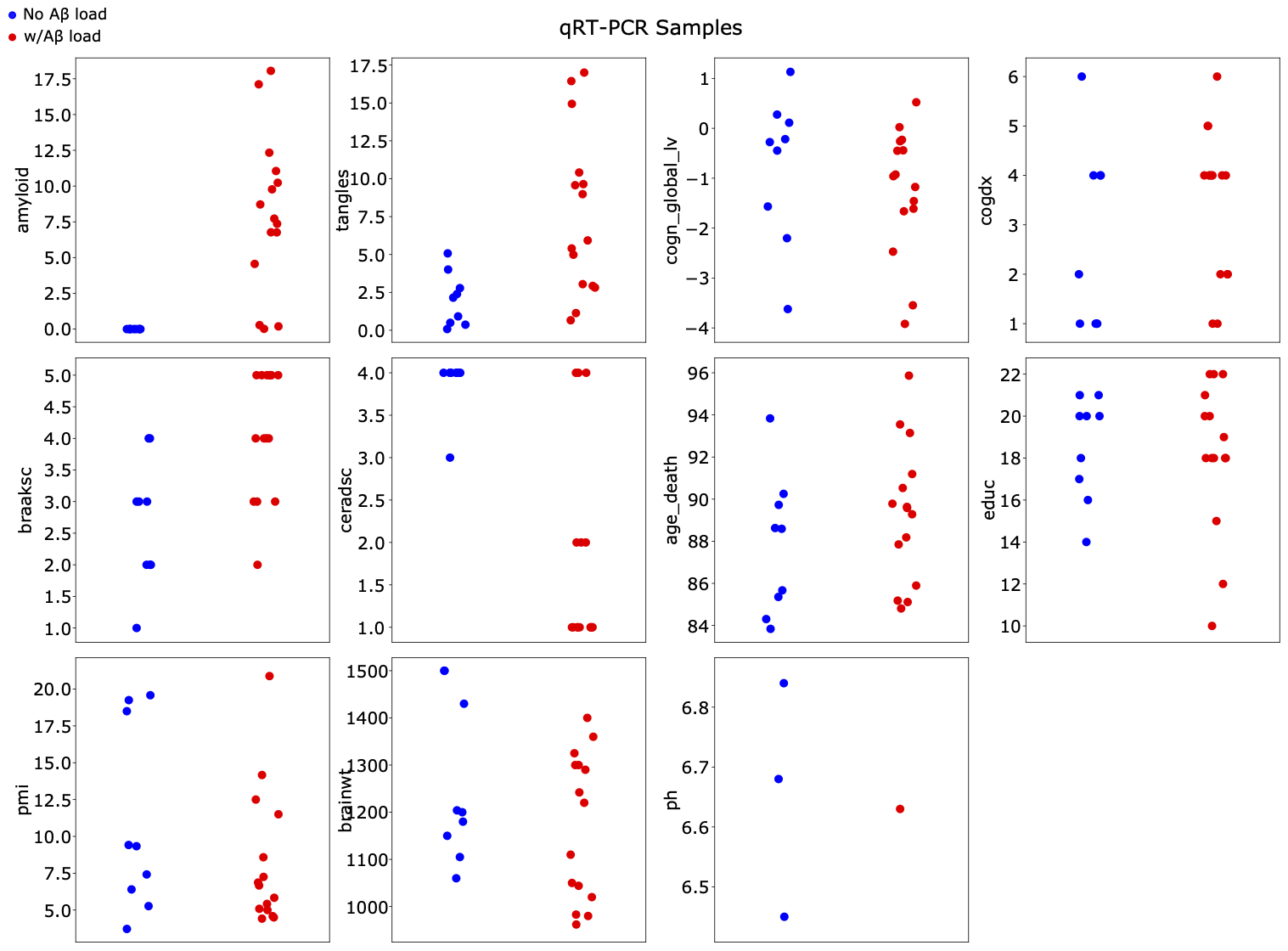

Supplement: Supplementary file 14 [file Presentation_1.zip › Supplementary Figure 19.TIFF]

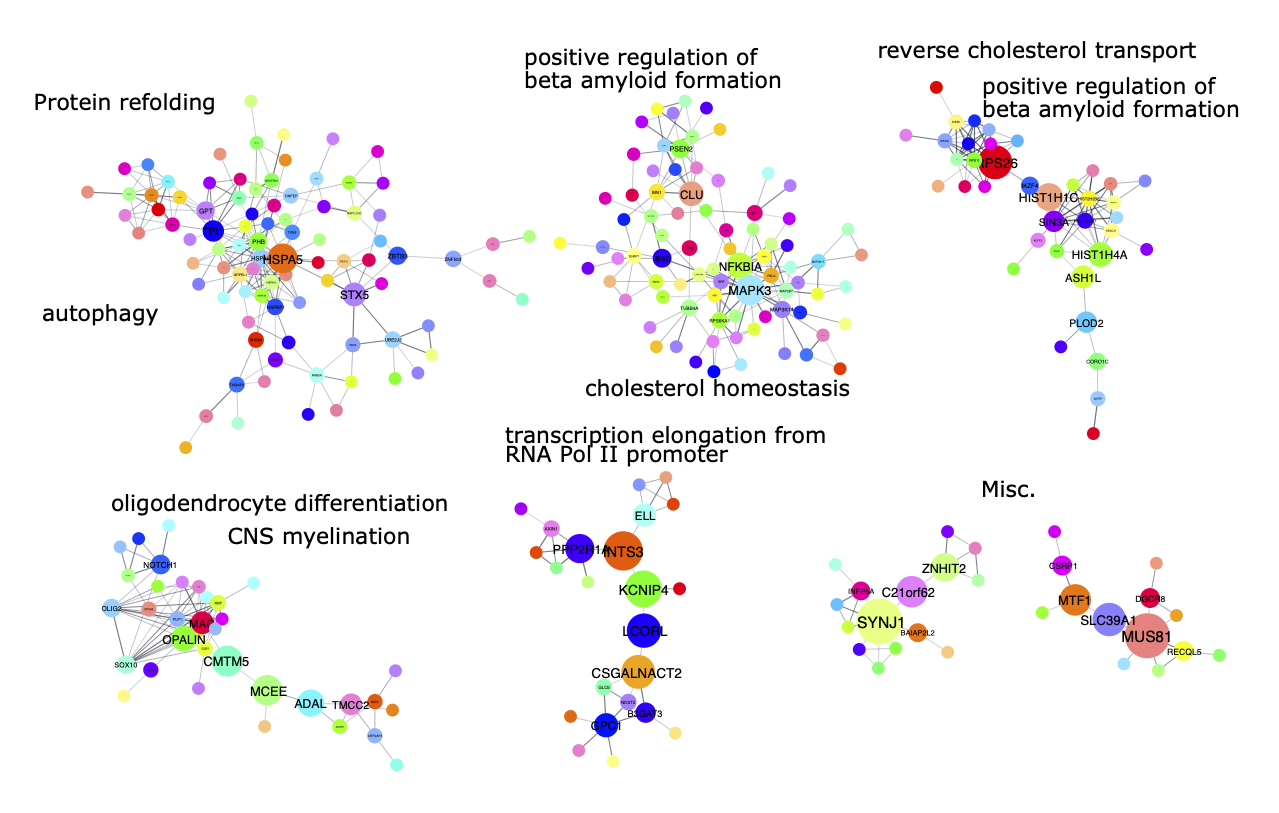

Supplement: Supplementary file 14 [file Presentation_1.zip › Supplementary Figure 20.TIFF]

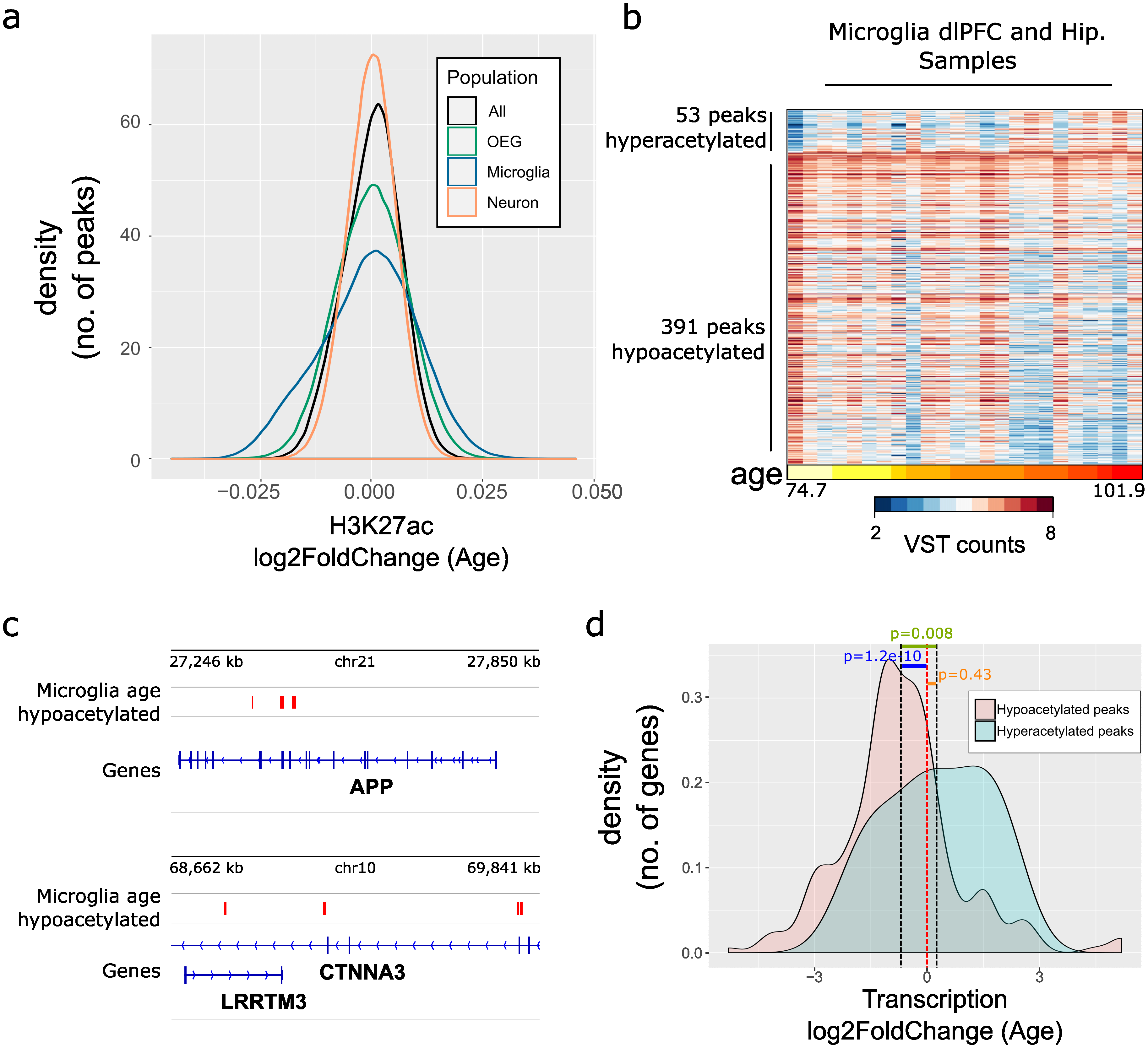

Supplement: Supplementary file 14 [file Presentation_1.zip › Supplementary Figure 21.TIFF]

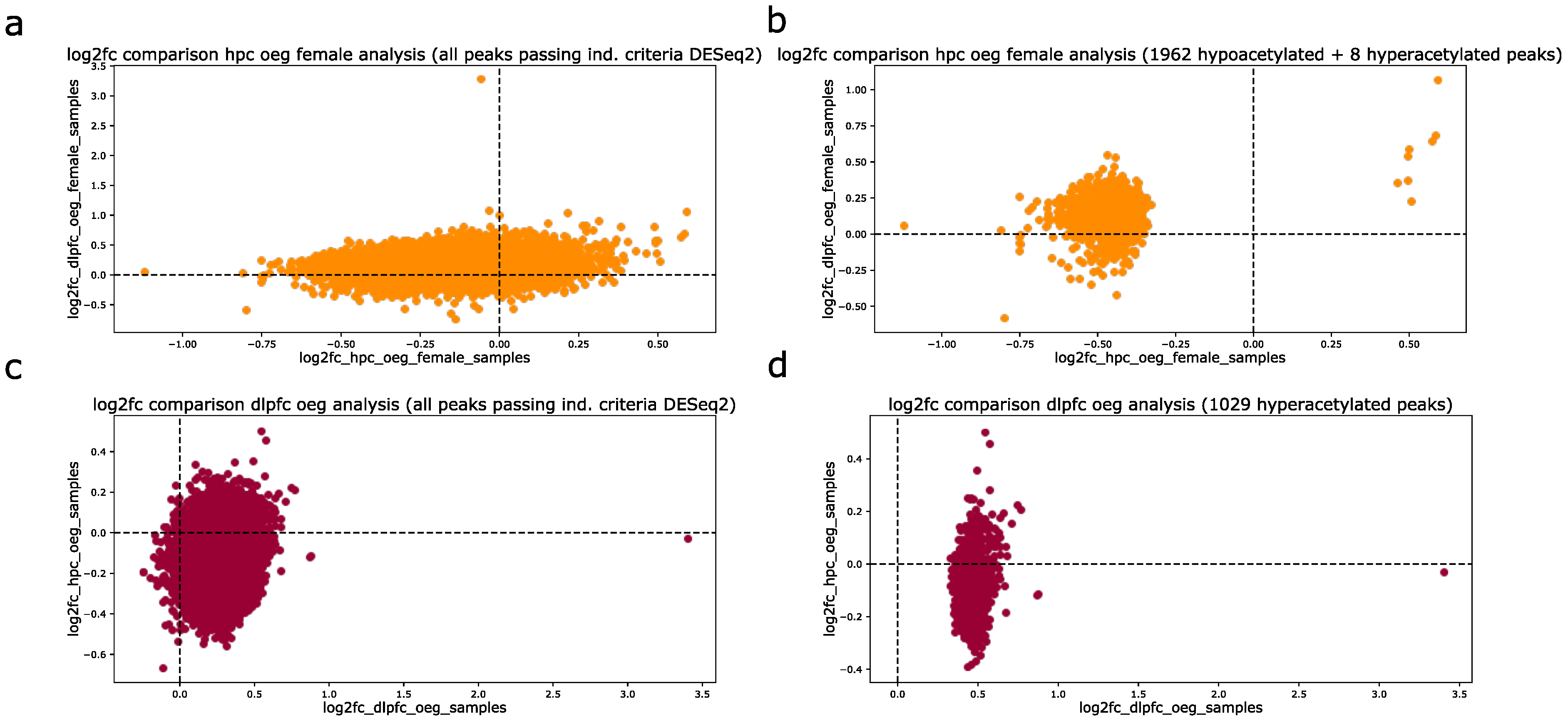

Supplement: Supplementary file 14 [file Presentation_1.zip › Supplementary Figure 22.TIFF]
